# Toxic Accumulation of LPS Pathway Intermediates Underlies the Requirement of LpxH for Growth of *Acinetobacter baumannii* ATCC 19606

**DOI:** 10.1371/journal.pone.0160918

**Published:** 2016-08-15

**Authors:** Daryl L. Richie, Kenneth T. Takeoka, Jade Bojkovic, Louis E. Metzger, Christopher M. Rath, William S. Sawyer, Jun-Rong Wei, Charles R. Dean

**Affiliations:** Novartis Institutes for BioMedical Research, Emeryville, CA, United States of America; University of Padova, Medical School, ITALY

## Abstract

The lipid A moiety of lipopolysaccharide (LPS) is the main constituent of the outer leaflet of the Gram-negative bacterial outer membrane (OM) and is essential in many Gram-negative pathogens. An exception is *Acinetobacter baumannii* ATCC 19606, where mutants lacking enzymes occurring early in lipid A biosynthesis (LpxA, LpxC or LpxD), and correspondingly lacking LPS, can grow. In contrast, we show here that LpxH, an enzyme that occurs downstream of LpxD in the lipid A biosynthetic pathway, is essential for growth in this strain. Multiple attempts to disrupt *lpxH* on the genome were unsuccessful, and when LpxH expression was controlled by an isopropyl β-d-1-thiogalactopyranoside (IPTG) inducible promoter, cell growth under typical laboratory conditions required IPTG induction. Mass spectrometry analysis of cells shifted from LpxH-induced to uninduced (and whose growth was correspondingly slowing as LpxH was depleted) showed a large cellular accumulation of UDP-2,3-diacyl-GlcN (substrate of LpxH), a C14:0(3-OH) acyl variant of the LpxD substrate (UDP-3-*O*-[(*R*)-3-OH-C_14_]-GlcN), and disaccharide 1-monophosphate (DSMP). Furthermore, the viable cell counts of the LpxH depleted cultures dropped modestly, and electron microscopy revealed clear defects at the cell (inner) membrane, suggesting lipid A intermediate accumulation was toxic. Consistent with this, blocking the synthesis of these intermediates by inhibition of the upstream LpxC enzyme using CHIR-090 abrogated the requirement for IPTG induction of LpxH. Taken together, these data indicate that LpxH is essential for growth in *A*. *baumannii* ATCC19606, because, unlike earlier pathway steps like LpxA or LpxC, blockage of LpxH causes accumulation of detergent-like pathway intermediates that prevents cell growth.

## Introduction

**Fig 1:**
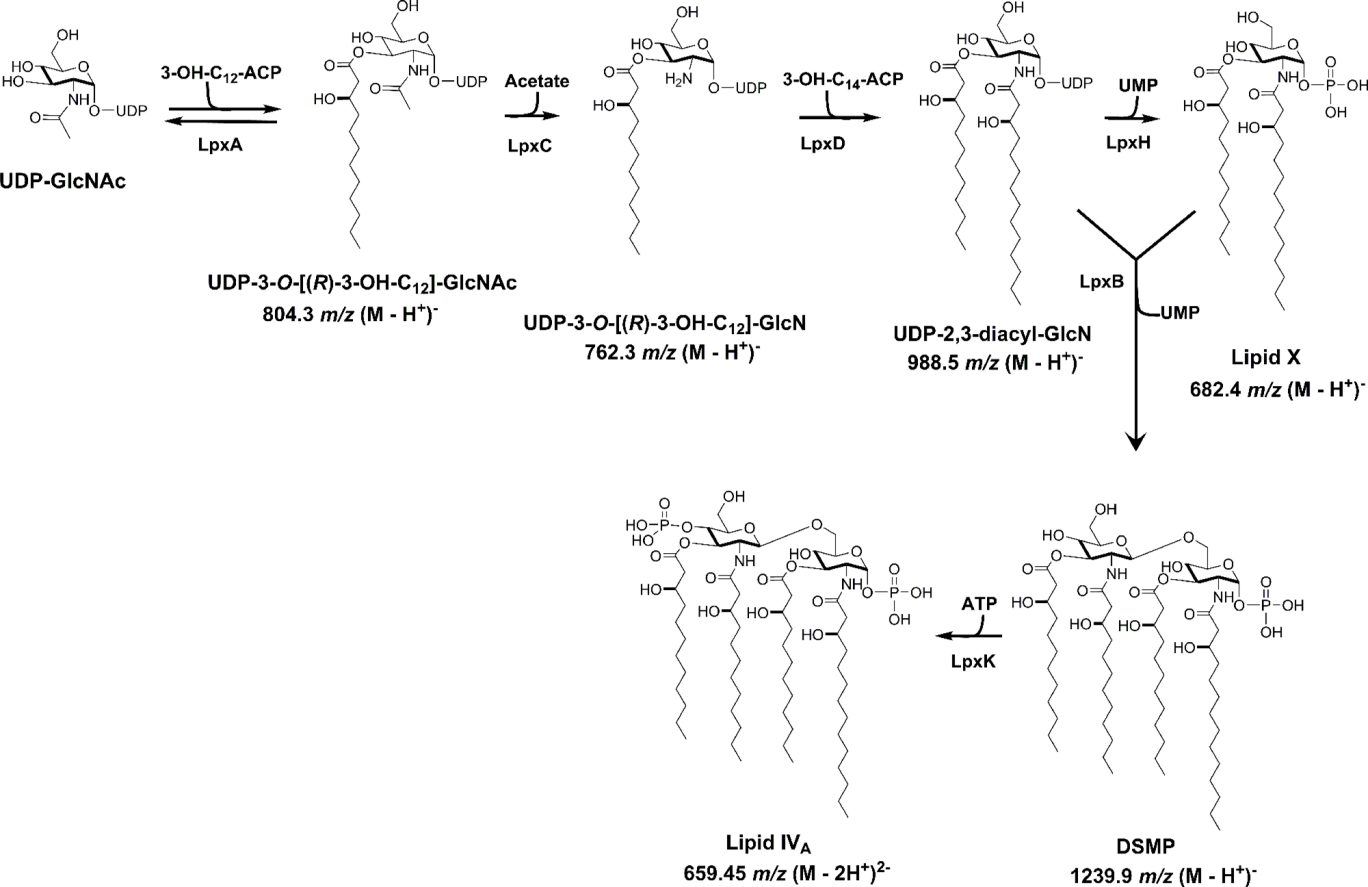
Predicted Lipid A biosynethic pathway in *A*. *baumannii* ATCC 19606.

In addition to the cytoplasmic phospholipid membrane, Gram-negatives are defined by the presence of an outer membrane (OM). The OM is asymmetrical having a phospholipid inner leaflet and an outer leaflet comprised largely of lipopolysaccharide (LPS). LPS consists of a hydrophobic lipid A anchor which forms the outer leaflet of the OM, decorated with core and O-antigen polysaccharides that extend out from the cell surface [1, 2]. Many of the genes encoding enzymes involved in lipid A biosynthesis are essential for growth and conserved across several important Gram-negative pathogens, including the first six enzymes of the Raetz Pathway of lipid A biosynthesis (Fig 1) [2]. The first step, catalyzed by LpxA, is the acylation of UDP-GlcNAc to form UDP-3-*O*-[(*R*)-3-OH-C_12_]-GlcNAc, with the length of the incorporated β-hydroxyacyl chain varying among species [3–5]. The committed step in the pathway is the deacetylation of UDP-3-*O*-[(*R*)-3-OH-C_12_]-GlcNAc, catalyzed by LpxC, to produce UDP-3-*O*-[(*R*)-3-OH-C_12_]-GlcN [6]. LpxD then catalyzes the addition of a second β-hydroxyacyl chain from acyl-ACP to the LpxC product, generating UDP-2,3-diacyl-GlcN [7]. Again, the acyl chain length incorporated varies among different bacterial species [8]. The membrane associated phosphodiesterase LpxH then catalyzes the hydrolysis of UDP-2,3-diacyl-GlcN to generate lipid X, and the inverting glycosyltransferase LpxB catalyzes the condensation of UDP-2,3-diacyl-GlcN and lipid X to form a tetracylated disaccharide 1-monophosphate (DSMP) [9]. DSMP is then phosphorylated at the 4'- position by the integral membrane kinase LpxK [10] yielding lipid IV_A_ (Fig 1). While additional aclytransferases, glycosyltransferases, kinases, and other modifying enzymes catalyze the further assembly of mature lipid A species [11], lipid IV_A_ is the minimal lipid A structure required for viability of *E*. *coli*, where this pathway has been most extensively studied [1, 12]. The minimal LPS structure required for growth of other Gram-negatives differs however, for example LPS having the phosphorylated core region is required for growth of *P*. *aeruginosa* [13–15] and as described below, certain Gram-negatives such as *A*. *baumannii* ATCC 19606 can survive in the absence of any LPS [16–18].

*A*. *baumannii* is emerging as an important multidrug resistant Gram-negative pathogen in hospital infections [19–21]. Clinical resistance to colistin, often the last line of effective treatment, is primarily associated with mutations in *pmrAB*, encoding a two-component regulator controlling phosphoethanolamine modification of the lipid A located in the outer leaflet of the protective outer membrane [16, 18, 22, 23]. This modification reduces the negative charge status of the lipid A, reducing the ability of the cationic colistin molecule to interact with the outer membrane which is a necessary step for its antibacterial activity. Resistance to colistin in some strains of *A*. *baumannii* can also result from complete loss of LPS caused by mutations in genes encoding enzymes early in the lipid A biosynthetic pathway (*lpxA*, *lpxC*, and *lpxD*) [16–18, 24]. This has only recently been uncovered and may be mainly an *in vitro* phenomenon since it is unclear under what circumstances cells lacking LPS would survive and cause infection in a human host [16, 25, 26], but this expands the list of Gram-negatives (including *Neisseria meningitidis* and *Moraxella catarrhalis*) that are now known to be able to survive without LPS [24, 27–30]. Nonetheless, since LPS is clearly essential in many important Gram-negative pathogens, including *Klebsiella pneumoniae*, *P*. *aeruginosa*, *and E*. *coli*, LPS biosynthesis and correspondingly, the transport and assemble of LPS into the OM, remain the focus of intense interest as targets for the development of novel antibacterials [31–37].

Given the detergent-like nature of some of the intermediates of the LPS pathway, it has been proposed that inhibition of certain biosynthetic or transport/assembly steps could be particularly growth inhibitory, and by extension might be excellent targets for the discovery of novel antibacterial agents for Gram-negatives, due to toxic accumulation of these pathway intermediates [7, 38, 39]. Supporting this notion, we showed that disrupting LPS translocation to the outer membrane by deleting *lptD* impaired cell envelope integrity, caused accumulation and/or mislocalization of lipid IV_A_ and reduced growth rate in *A*. *baumannii* ATCC 19606 (which tolerates loss of LPS) [40]. The growth defect was reversed by inhibition of the early lipid A biosynthetic step at LpxC, presumably by reducing synthesis of these intermediates, thereby preventing toxic accumulation. Intriguingly, no mutations in genes occurring later than *lpxD* in the lipid A biosynthetic pathway (e.g. *lpxH*, *lpxB*, *lpxK*) were reported in *A*. *baumannii* ATCC19606 colistin resistance selection experiments [15]. This suggested the possibility that such mutations are not tolerated (i.e. these genes are essential for growth) or such mutations do not confer colistin resistance [16]. Based on this, the aim of this study was to determine if LpxH is essential for growth in *A*. *baumannii* ATCC 19606. Utilizing an isopropyl β-d-1-thiogalactopyranoside (IPTG)-regulated expression system we showed that *A*. *baumannii* ATCC 19606 cells depleted for LpxH were unable to grow. Cells depleted of LpxH strongly accumulated the detergent-like LpxH substrate molecule UDP-2,3-diacyl-GlcN and electron microscopy revealed clear defects at the cell (inner) membrane, consistent with toxic accumulation of a detergent like molecule at the membrane [39]. Consistent with this, the requirement for LpxH for growth was abrogated when synthesis of these intermediates was prevented by chemical inhibition of LpxC, upstream of LpxH in the pathway (Fig 1). Overall this shows that LpxH is essential for growth in *A*. *baumannii*, despite the fact that lipid A (LPS) itself is not essential and this stems from the toxic accumulation of detergent like pathway intermediates upon loss of LpxH function.

## Materials and Methods

### Bacterial strains, plasmids and growth conditions

The bacterial strains used in this study were *A*. *baumannii* ATCC 19606 from the American Type Culture Collection and an IPTG-regulated *lpxH* strain described below (NB48062-JWK0133). The multicopy plasmid pNOV108 was generated to provide increased LacI repression of *lpxH* (described below) and oligonucleotide primers used in this study are shown in S1 Table. Cells were routinely grown in Mueller-Hinton II (MHIIB) Broth (Cation-Adjusted) (3.0 g/L beef extract, 17.5 g/L acid hydrolysate of casein, 1.5 g/L starch, 20–25 mg/L calcium, 10–12.5 mg/L magnesium) or agar. Lysogeny Broth (LB) was used for transformations (10 g/L tryptone, 5 g/L yeast extract, and 10 g/L NaCl).

### Construction of an *A*. *baumannii* IPTG inducible LpxH strain NB48062-JWK0133

**Fig 2:**
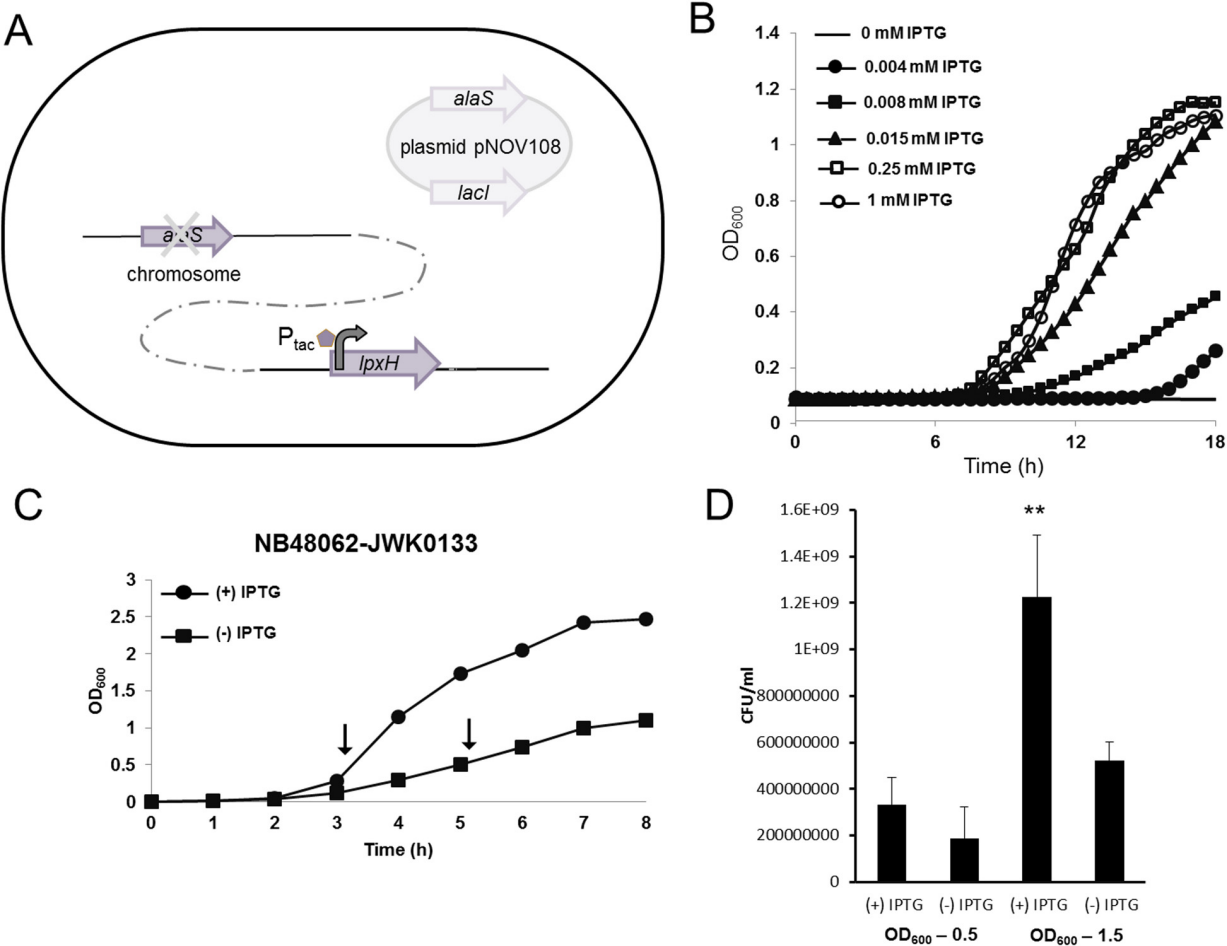
Schematic illustration of the *lpxH* regulated expression strain NB46082-JWK0133 and its dependence on IPTG induction for cell growth. (A) The chromosomal allele of *lpxH* is regulated by the P_tac_ promoter (inducible by IPTG). Plasmid pNOV108 provided extra copies of *lacI* to enhance repression of *lpxH* in the absence of IPTG. pNOV108 also contained *alaS* so that it could be maintained by complementation of an *alaS* deletion on the chromosome, eliminating the need for antibiotic selection. (B) Growth of NB48062-JWK0133 was IPTG dependent. C) Sub-culture growth curve of NB48062-JWK0133 plus or minus IPTG. Arrows indicate time points of sample collection for LCMS-MRM analysis, CFU determination, TEM images, RT-qPCR, and CHIR-090 rescue. D) A significant loss in viability (*, *P* ≤ 0.01) was observed at 1.5 OD_600_ under LpxH depletion conditions (-IPTG) compared to inducing conditions (+IPTG).

For IPTG-controlled expression of LpxH, the P_tac_ promoter and *lacI* were inserted in front of the *lpxH* gene on the chromosome of *A*. *baumannii* ATCC 19606. The linear DNA construct used to insert the P_tac_ promoter in front of *lpxH* was made by linking fragments encompassing the coding region of *lpxH* and 500 bp upstream of *lpxH* from *A*. *baumannii* ATCC 19606 with a kanamycin resistance cassette (*aph*), and *lacI*-P_tac_ promoter fragment (S1 Fig). This DNA fragment (8 μg) was then electroporated into *A*. *baumannii* ATCC 19606. To make electrocompetent *A*. *baumannii*, cells were grown overnight in Lysogeny broth (LB) at 37°C, diluted 1:100 in fresh LB and grown to around OD_600_ = 0.6. The cells were then put on ice for 2 min and washed with 10% glycerol 3 times before resuspending in 70 μL 10% ice cold glycerol for electroporation. After electroporation, clones containing the insert on the genome were selected on LB agar containing 50 μg/ml kanamycin and 1 mM IPTG. The insert was then verified by sequencing. However, expression of LpxH in this strain was not regulated tightly enough to prevent growth under non-inducing conditions. To increase repression, extra copies of the repressor LacI were provided via a *lacI*-containing multicopy plasmid (pNOV108). Since regulation of LpxH would be expected to impact the cell envelope (membrane) we anticipated difficulties using traditional antibiotic selection to maintain the plasmid in this strain. To address this, the essential gene *alaS* (alanyl-tRNA synthetase) was incorporated into plasmid pNOV108, which was then transformed into the *A*. *baumannii* strain having the P_*tac*_ regulated *lpxH*, and then *alaS* was inactivated on the chromosome. Therefore pNOV108, which provided extra copies of the LacI repressor, had to be maintained in these cells in order for them to grow. Plasmid pNOV108 was generated by circular polymerase extension cloning (CPEC, [41]). A map of pNOV108 is shown as S2 Fig and the sequence can be found under NCBI accession number KX580601. The DNA fragment used to inactivate *alaS* on the genome was generated by overlap extension PCR [42] as described in S3 Fig. Briefly, upstream and downstream regions of *alaS* were amplified from *A*. *baumannii* ATCC 19606 genomic DNA and joined with a gentamicin resistance cassette (*accC1*) using overlap PCR. The final product was gel purified and then introduced into electrocompetent *A*. *baumannii* prepared as described above. Clones with *alaS* replaced by *aacC*1 on the genome were selected on LB agar containing 100 μg/ml gentamicin and verified by sequencing and one clone NB48062-JWK0133 (Fig 2A) was used for further study. IPTG regulation of *lpxH* was confirmed by RT-qPCR to have a circa 25-fold change in transcript levels between induced and uninduced growth conditions (S4 Fig).

### Determination of LpxH essentiality for growth and growth rescue by the LpxC inhibitor CHIR-090

To determine if LpxH was essential for growth under standard laboratory conditions, the *lpxH* regulated strain (NB48062-JWK0133) was cultured in the presence or absence of IPTG (0–1 mM). Cells from a frozen stock were streaked on MHIIB plates supplemented with 1 mM IPTG and incubated overnight at 37°C. The following day, cell suspensions were prepared at an OD_600_ of 0.1 and diluted 1:10,000. Next 100 μL was added to the wells of a 96-well plate and incubated at 37°C and the OD_600_ was measured every 30 min using a Spectramax with Softmax^®^ Pro software v 5.4.1.

To determine if inhibiting LpxC could rescue growth during LpxH depletion, NB48062-JWK0133 was grown overnight at 37°C on Mueller-Hinton Agar (MHA) supplemented with 1 mM IPTG. The following day, cells were suspended in 1 mL of MHIIB (1.5 mL microcentrifuge tube), collected by centrifugation and resuspended in fresh MHIIB for a total of 3 washes to remove trace amounts of IPTG. After the final wash, cells were suspended in 5 mL of MHIIB and the OD_600_ was adjusted to 0.01. Next, 100 μL of the cell suspension was spread on a fresh MHIIB plate and allowed to dry. Sterile paper disks (Remel R55054) were added to the center of the plates and inoculated with 10 μL of DMSO (Sigma), IPTG (1 mM, Calbiochem) or CHIR-090 (LpxC inhibitor) (12.8 mg/mL, purchased from Axon 2000). The plates were incubated at 37°C for 24 hours before images were taken using a BIO-RAD ChemiDoc^Tm^ XRS+ with Image Lab^TM^ 3.0 software.

To establish the absolute concentrations of CHIR-090 required for the rescue effect, a liquid broth-based assay was used. CHIR-090 was dissolved in DMSO at 6.4 mg/ml (100-fold higher than the final assay concentration of 64 μg/mL). In a 96-well plate, sequential 2-fold serial dilutions were made in DMSO from wells 3 to 11 (corresponding final assay concentration of 0.25–64 μg/ml) leaving well 1 as the media only control, well 2 as the DMSO vehicle control and well 12 for the IPTG (1 mM final concentration) growth control. Using a 12-channel electronic pipette, 1 μL of each 100× drug concentration, including the DMSO only control, were transferred into a new 96-well U-bottom plate (Greiner bio-one, 650162). Next, using an 8-channel electronic pipette, the drug dilutions were then diluted 100-fold with the overnight inoculum described below and plates were then incubated for six hours at 37°C before fluorescence reading (545ex (nm)– 590em (nm)) on the SpectraMax with Softmax^®^ Pro software v 5.4.1. To generate the overnight culture, NB48062-JWK0133 was grown overnight in MHIIB with 1 mM IPTG for induction of LpxH expression. The following day, the cells were diluted to 0.1 OD_600_ and further 100-fold in fresh MHIIB media with 10% alamar blue (Thermo Scientific). The experiment was done in triplicate.

To determine the effect of LpxH depletion conditions (- IPTG) on viable cell counts, *A*. *baumannii* NB48062-JWK0133 was grown overnight in MHIIB and 1 mM IPTG for induction of LpxH expression. The following day the cells were diluted to an OD_600_ of 0.05 in 50 mL of MHIIB + 1 mM IPTG and grown at 37°C with shaking until an OD_600_ of 0.5 was reached. Cells were then collected by centrifugation, washed twice with MHIIB, and suspended in MHIIB at the original volume of 50 mL. The cell suspension was then diluted 1:100 in 50 mL of fresh MHIIB plus or minus IPTG in 250 mL flasks. The cultures were grown at 37°C with shaking and samples were collected every hour for OD_600_ measurement. When the cultures reached an OD_600_ of approximately 0.5 and 1.5, 5 mL were removed the cultures were adjusted to 0.5 or 1.5 OD_600_ and 100 μL was removed followed by 2-fold serial dilution performed in MHIIB. Next 100 μL of the serial dilutions was plated in duplicate on MHIIB plates supplemented with 1 mM IPTG. Plates were incubated for 24 hours at 37°C before CFU determination. The experiment was done in triplicate and results are of two independent experiments.

### Liquid Chromatography-Mass Spectrometry (LCMS) detection of lipid A precursors

*A*. *baumannii* NB48062-JWK0133 was grown overnight in MHIIB supplemented with 1 mM IPTG to induce LpxH expression. The following day the cells were diluted to an OD_600_ of 0.05 in 50 mL of MHIIB + 1 mM IPTG and grown at 37°C with shaking until an OD_600_ of 0.5 was reached. Cells were then collected by centrifugation, washed twice with MHIIB, and suspended in MHIIB at the original volume of 50 mL. The cell suspension was then diluted 1:100 in 50 mL of fresh MHIIB plus or minus IPTG in 250 mL flasks. The cultures were grown at 37°C with shaking and samples were collected every hour for OD_600_ measurement. When the cultures reached an OD_600_ of 0.5, 5 mL were removed, and the cells were pelleted and lysed with SoluLyse™ Protein Extraction Reagent (GenLantis) as previously described [40]. For CHIR-090 treatment, the LpxH depletion conditions were repeated (in triplicate) and the minus IPTG cells were incubated in the presence or absence of CHIR-090 at 8 and 16 μg/ml CHIR-090 until 0.5 OD_600_ was reached. Multiple-reaction monitoring (MRM) relative quantification of LPS intermediates was performed as previously described using a reversed-phase Agilent 1290 liquid chromatography system and an AB Sciex 4000 triple quadrupole mass spectrometer in the negative ionization mode [40]. MRM pairs, and compound dependent parameters are outlined in S2 Table and as previously described [40]. Two precursor ion scan (PIS) experiments were run (S5 Fig) to identify the abundant acyl-chain variants associated with each LPS intermediate in both wild-type and LpxH depletion conditions. The first PIS monitored the precursors of the common UDP-derived product ion at 385 *m/z* (UDP-3-*O*-acyl]-GlcNAc, UDP-3-*O*-acyl]-GlcN, UDP-2,3-diacyl-GlcN, Metlin MID: 41549), and the second PIS monitored precursors of the phosphate ion at 79 m/z (Lipid X, DSMP, lipid IV_A_). Retention times were used as an additional constraint in this experiment as noted in S3 Table. The transitions for abundant acyl-chain variants were then added to the transition S2 Table and quantitated across our entire dataset in addition to those previously reported [40]. Data were collected with three biological replicates, and each experiment was performed at least three times. Two-tailed student t-test was used for statistical analysis. A comprehensive overview outlining the process for identification of LPS intermediates is provided as supplementary material (S1 File, S6–S18 Figs, S2–S4 Tables).

### Thin section transmission electron microscopy (TEM)

LpxH depleted cell cultures were prepared according to the method described above for LCMS detection of lipid A precursors, and one milliliter of the +IPTG and–IPTG cultures at 0.5 OD_600_ were pelleted by centrifugation at 4000 × g for 10 min at room temperature. Cells were resuspended and fixed with Tousimis Fixative (1.5% glutaraldehyde/1% formic acid in 0.12M Sorensen’s Buffer) from Tousimis Research Corporation and submitted to the University of California, Davis, Center for Biophotonics Science & Technology (Sacramento, CA, USA) for embedding and thin section preparations.

## Results and Discussion

### LpxH is required for growth under standard laboratory conditions in *A*. *baumannii* ATCC 19606

Although *A*. *baummannii* ATCC19606 can grow without LPS [16], loss-of-function mutations preventing LPS synthesis in *A*. *baumannii* ATCC 19606 have so far been reported only for genes encoding enzymes occurring very early in the lipid A biosynthetic pathway (*lpxA*, *lpxC* and *lpxD*, Fig 1) but not in genes encoding later steps (e.g. *lpxH*, *lpxB*, *lpxK*) [16–18]. This suggested that disruption of the more distal biosynthetic LPS pathway genes may not be tolerated, since the earlier steps could conceivably be generating pathway intermediates that accumulate to toxic levels if later biosynthetic steps were blocked [38]. Indeed, even for the previously described mutants, an *A*. *baumannii* ATCC 19606 *ΔlpxD* mutant (LpxD mediates addition of the second *R*-3-hydroxymyristate chain to produce UDP-2,3-diacyl-GlcN) had a greater growth defect than did strains lacking *lpxA* or *lpxC*, suggesting accumulation of the UDP-3-*O*-[(*R*)-3-OH-C_14_]-GlcN (LpxD substrate) may somewhat impair growth due to its suspected detergent-like properties [7, 18]. To determine if an LPS biosynthetic enzyme downstream of LpxD was essential for growth in *A*. *baumannii* ATCC 19606, we first attempted to delete *lpxH*, which encodes the fourth step in lipid A biosynthesis which is immediately downstream of LpxD (Fig 1) [9, 43, 44]. Multiple attempts to delete *lpxH* were unsuccessful, suggesting that LpxH was in fact essential for growth. To explore this further, we constructed a strain with LpxH expression under the control of the P_tac_ promoter (NB48062–JWK0133, Fig 2A). Growth of NB48062–JWK0133 was dependent on IPTG in the standard laboratory medium used here, establishing that *LpxH* was essential for growth in *A*. *baumannii* ATCC19606, despite the fact that LPS itself or other biosynthetic enzymes such as LpxC are not (Fig 2B). Furthermore, cultures shifted from LpxH-induced to non-induced culture conditions, to allow depletion of LpxH and a corresponding progressive slowing of growth, had a modest but statistically valid decrease in viable cell counts (Fig 2C and 2D). This suggests that interference with LpxH could also be cidal, however, this is difficult to characterize conclusively since some *lpxH* transcript was still detectable under non-inducing conditions (S4 Fig).

### Depletion of LpxH causes accumulation of LPS pathway intermediates in *A*. *baumannii* ATCC 19606

**Fig 3:**
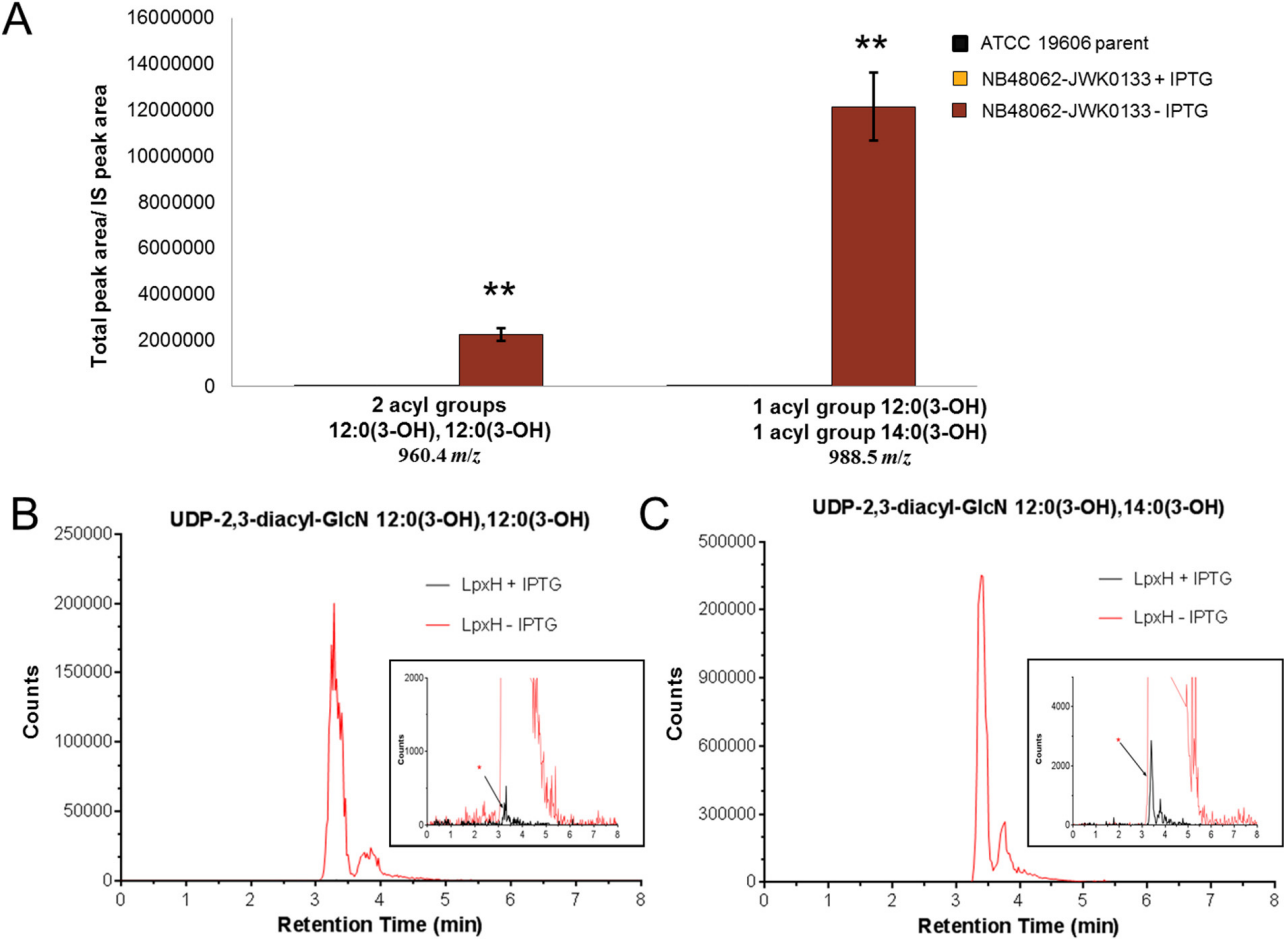
Depletion of LpxH causes accumulation of UDP-Diacyl-GlcN (LpxH substrate). A) The LCMS-MRM quantification of lipid A precursor UDP-Diacyl-GlcN is shown for *A*. *baumannii* ATCC 19606 parent and NB48062-JWK0133 under inducing and non-inducing conditions. The *m/z* [M–H^+^]^-^ for UDP-Diacyl-GlcN containing 2 acyl groups, 12:0(3-OH), 12:0(3-OH) is 960.5 and for UDP-Diacyl-GlcN with 1 acyl group 12:0(3-OH) and 1 acyl group 14:0(3-OH) the *m/z* [M–H^+^]^-^ is 988.5. Experiments were performed in triplicate and bars show the mean value and SD (two-tailed student t-test, **, P<0.01) between NB46082-JWK0133 in the presence or absence of IPTG. Data shown were normalized to an internal standard (IS) as previously described [40]. B) Extracted ion chromatogram (EIC) of NB48062-JWK0133 in the presence or absence of IPTG for 2 acyl groups, 12:0(3-OH), 12:0(3-OH). C) Extracted ion chromatogram of NB48062-JWK0133 in the presence or absence of IPTG for 1 acyl group 12:0(3-OH) and 1 acyl group 14:0(3-OH).

**Fig 4:**
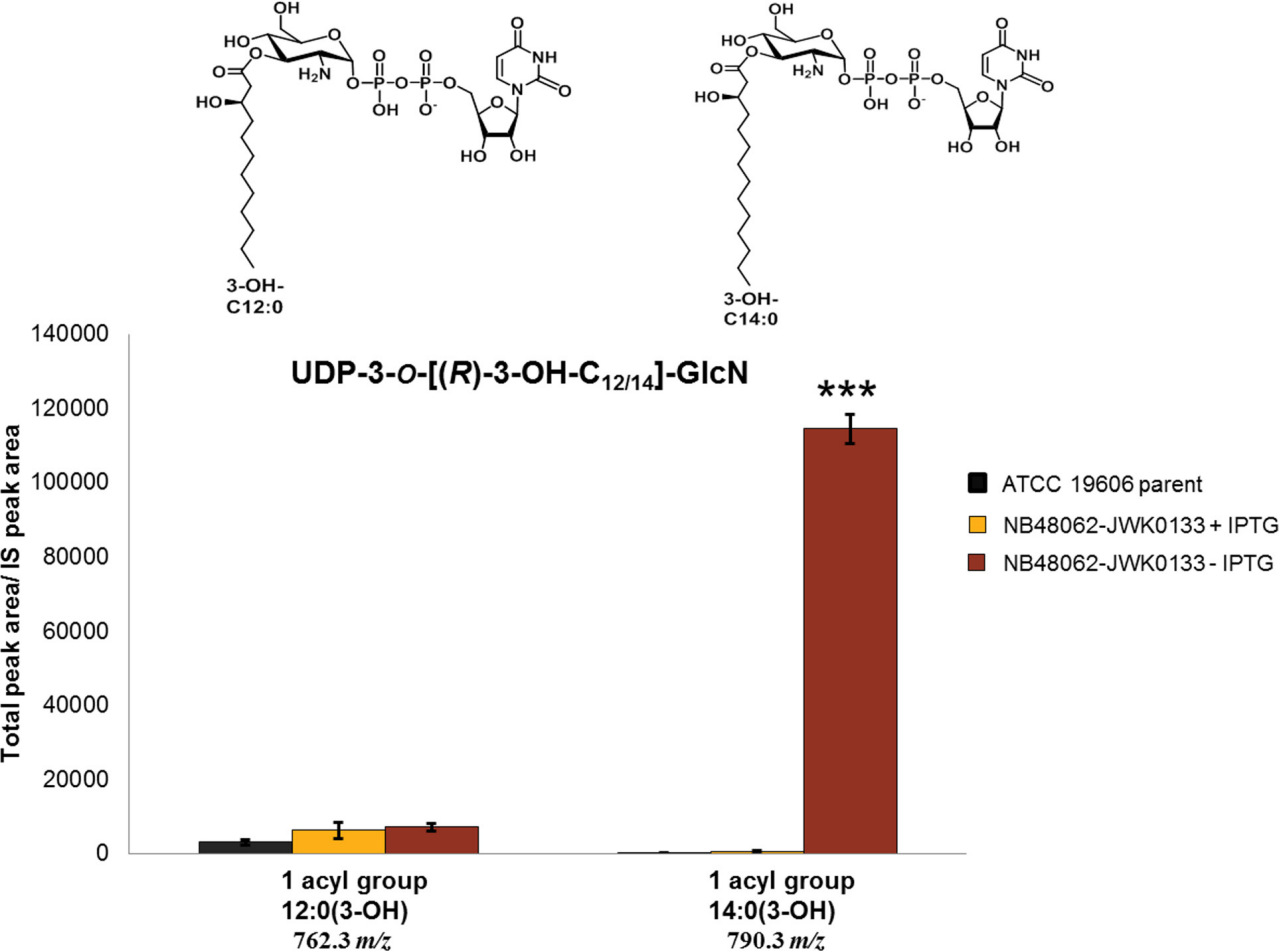
Depletion of LpxH causes accumulation of an alternative LpxC product containing a C14:0(3-OH) acyl chain. The LCMS-MRM quantification of UDP-3-*O*-[(*R*)-3-OH-C_12/14_]-GlcN for acyl group 12:0(3-OH) and for acyl group 14:0(3-OH) is shown for *A*. *baumannii* ATCC 19606 parent and NB48062-JWK0133 under inducing and non-inducing conditions. The experiment was performed in triplicate and bars show the mean value and SD (two-tailed student t-test ***, P<0.001) between NB46082-JWK0133 in the presence or absence of IPTG. Data shown was normalized to an internal standard (IS) as previously described [40].

**Fig 5:**
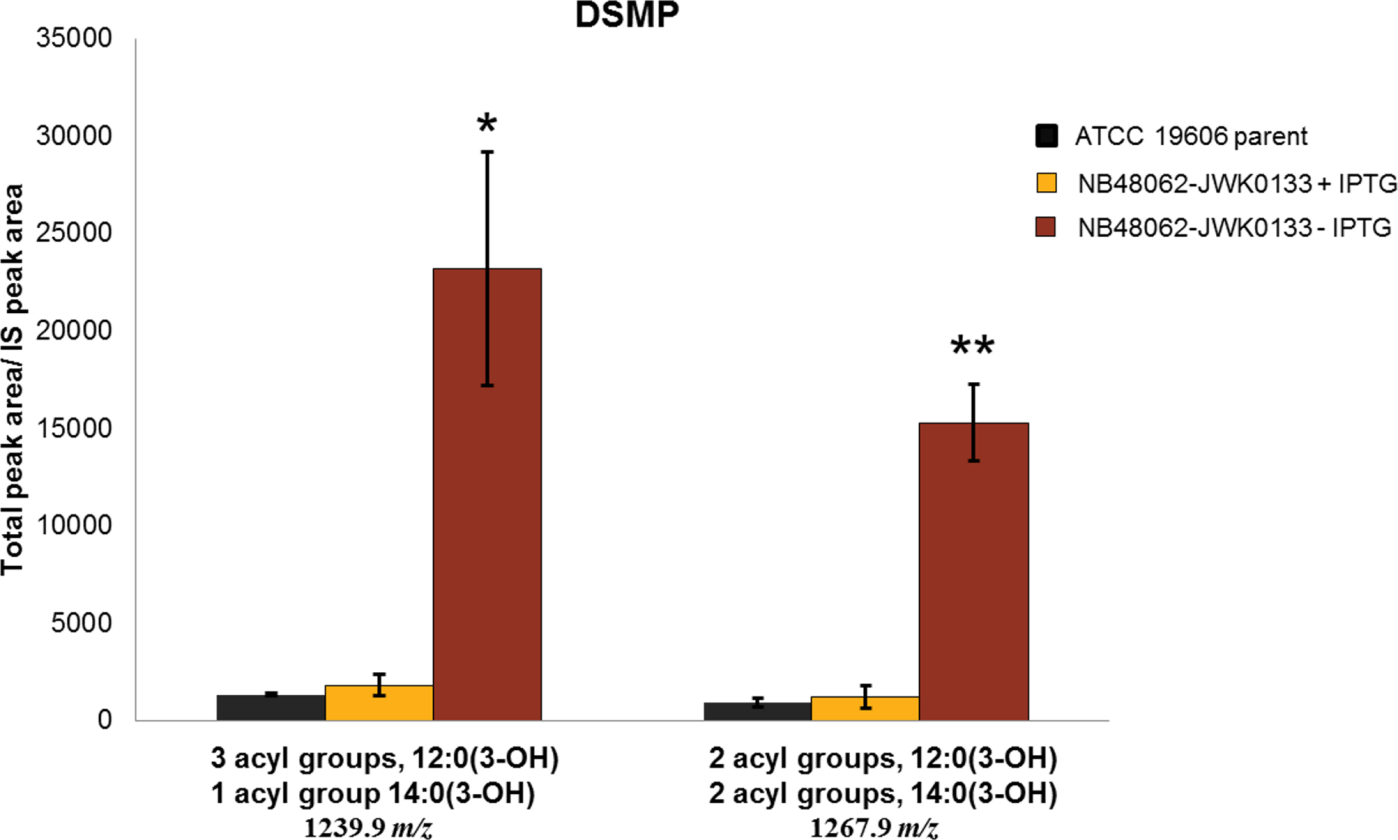
Depletion of LpxH causes accumulation of DSMP. (A) LCMS-MRM quantification of DSMP with three 12:0(3-OH) acyl groups and one 14:0(3-OH) acyl group and with two 12:0(3-OH) acyl groups and two 14:0(3-OH) acyl groups for *A*. *baumannii* ATCC 19606 parent and NB48062-JWK0133 under inducing and non-inducing conditions. The experiment was performed in triplicate and bars show the mean value and SD (two-tailed student t-test *, P<0.05 and **, P<0.01) between NB46082-JWK0133 in the presence or absence of IPTG). Data shown was normalized to an internal standard (IS) as previously described [40].

The observation that growth of *A*. *baumannii* ATCC19606 depended on LpxH, even though LPS itself, or genes encoding steps occurring early in the lipid A biosynthestic pathway, were not essential for growth, strongly suggested that toxic accumulation of a lipid A pathway intermediate(s) was a factor determining the dependence on LpxH for growth. Therefore, to examine if significant intermediate accumulation was occurring, we used LCMS to directly monitor lipid IV_A_ intermediate levels in LpxH-depleted cells and non-depleted cells. For this, we identified an appropriate inoculum of NB48062-JWK0133 to subculture into non-inducing (-IPTG) medium whereby progressive depletion of LpxH would reduce growth over time in liquid culture. This was designed to have growth curves level off at a point providing an adequate amount of cells for quantification of lipid IV_A_ intermediates using mass spectrometry. Under non-inducing conditions (-IPTG), LCMS analysis of pathway intermediates from UDP-3-*O*-[(*R*)-3-OH-C_12_]-GlcNAc through lipid IV_A_ showed a profound accumulation of UDP-2,3-diacyl-GlcN (LpxH substrate) comprised of acyl chain lengths of two C12:0(3-OH) fatty acids or one C14:0(3-OH) and one C12:0(3-OH) fatty acid at the 5 hour time point (0.5 OD_600_) along the depletion growth curve compared to induced conditions (+IPTG) or the wild type parent cells (Fig 2C and Fig 3A–3C). The accumulation of the pathway intermediate that is the substrate of LpxH provided a strong indication that these cells were in fact depleted of LpxH. This result is also reminiscent of previous studies where *E*. *coli* cells lacking the LpxH gene accumulated UDP-2,3-diacyl-GlcN to circa 10% of total cellular phospholipids as estimated by radiographic thin-layer chromatography [43]. Such an extreme increase of this detergent-like intermediate in both cases may imply a role for this intermediate in cellular toxicity. Depletion of LpxH did not cause a significant accumulation of UDP-3-*O*-[(*R*)-3-OH-C_12_]-GlcNAc (LpxA product) or the predicted LpxC product containing the C12:0(3-OH) fatty acid (UDP-3-*O*-[(*R*)-3-OH-C_12_]-GlcN) [22, 40], however, a significant accumulation of an alternative C14:0(3-OH) acyl chain variant (UDP-3-*O*-[(*R*)-3-OH-C_14_]-GlcN) was observed (Fig 4 and S19 Fig). This may also contribute to the cellular toxicity engendered by LpxH depletion. Finally, there was a substantial accumulation of disaccharide 1-monophosphate (DSMP) with varying acyl chain lengths upon LpxH depletion (Fig 5). This was intriguing since this intermediate is generated by enzymes downstream of LpxH in the lipid A pathway. Although it is possible that downstream accumulation of DSMP occurs due to hydrolysis of the accumulated UDP-2,3-diacyl-GlcN and subsequent *in vitro* catalysis to form DSMP, this seems unlikely since prepared samples showed no variation in lipid IV_A_ intermediate levels over 48 hours at room temp (data not shown). Alternative enzymes that hydrolyze UDP-2,3-diacyl-GlcN have been found in other Gram-negatives including Cdh (*E*. *coli*), LpxI (*Caulobacter crescentus*) and LpxG (*Chlamydia trachomatis*) [43–47] but BLAST searches did not uncover any obvious homologs of these in *A*. *baumannii* ATCC 19606. Since we harvested our cells at a point of strong growth inhibition, but not complete cessation (Fig 2C), the cells were presumably not completely depleted of LpxH (S4 Fig), and therefore some flux through the downstream lipid A pathway beyond LpxH may have still been occurring. We speculate that the accumulation of DSMP reflects either a regulatory response of certain downstream steps, or that the accumulations of LpxH and/or LpxD substrates caused by reduced LpxH interfered with LPS and/or phospholipid synthesis or transport, or inner membrane function generally. This may be consistent with recent computational analyses of LPS biosynthesis in *E*. *coli* suggesting complex interrelationships within, and between, the LPS and phospholipid pathways [48, 49].

### Depletion of LpxH causes clear morphological defects in *A*. *baumannii* ATCC 19606

**Fig 6:**
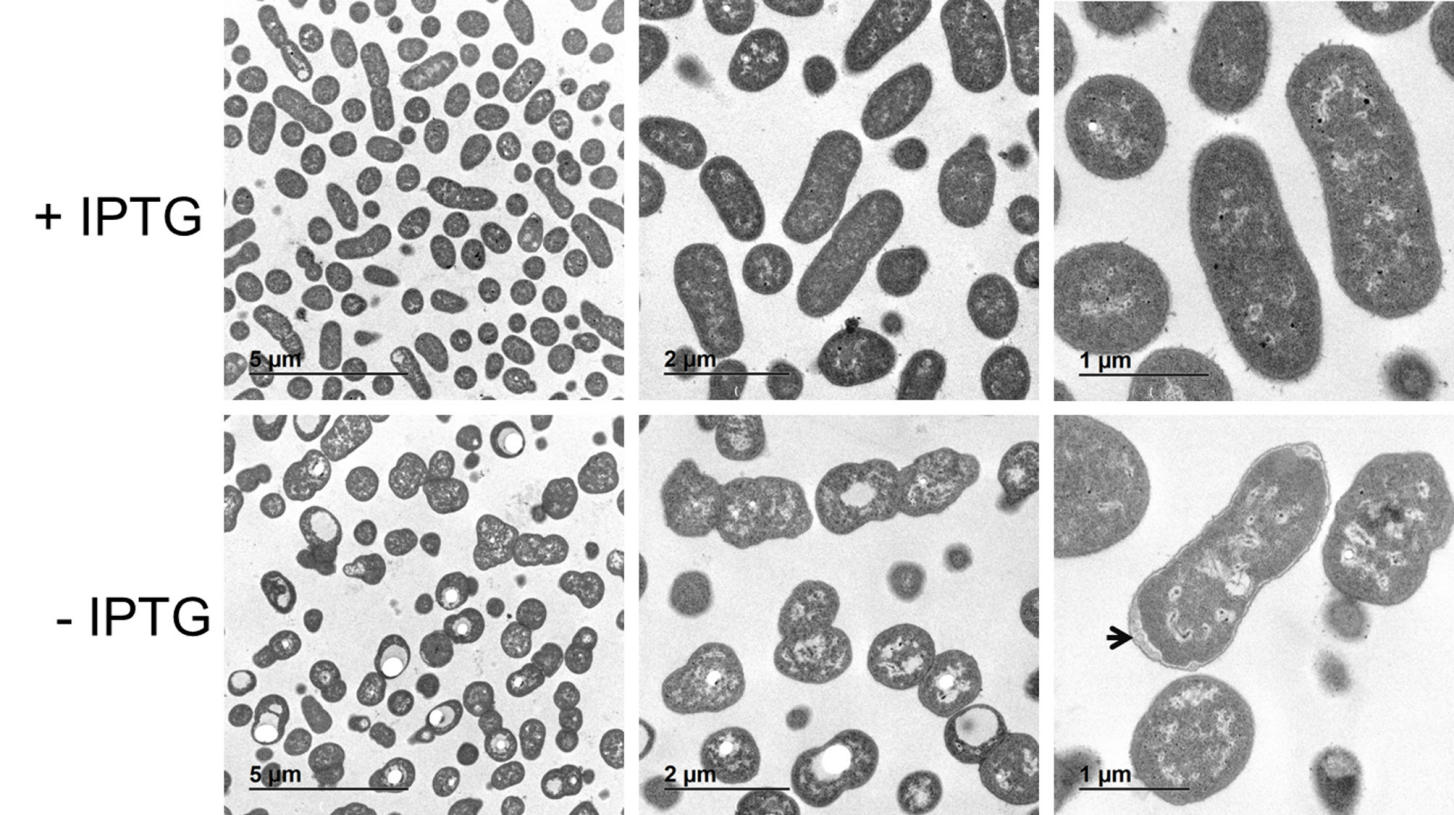
Transmission EM images of NB48062-JWK0133 plus or minus IPTG. LpxH depleted cells (-IPTG) display gross morphology changes. The experiment was repeated twice with similar results.

An *A*. *baumannii* mutant lacking LPS (and therefore the typical asymmetric LPS based OM), perhaps surprisingly did not exhibit any gross morphological defects when examined by electron microscopy [16]. In contrast to the case with loss of LpxA, *A*. *baumannii* ATCC 19606 requires LpxH to grow and as described above, cells depleted of LpxH accumulate the detergent-like molecule UDP-diacyl-GlcN (and other pathway intermediates). If the accumulation of these intermediates is toxic and occurs at the cell membrane we surmised that, unlike the case for cells deficient in LpxA, cells deficient in LpxH would exhibit severe morphological defects. Consistent with this, electron microscopy of LpxH depleted cells revealed profound morphological changes, particularly at the cell envelope/inner membrane (Fig 6) which would be expected for general interference with the inner membrane function by accumulation of a detergent like molecule [13, 33, 39].

### Inhibition of LpxC abrogates the dependence on LpxH expression for growth of strain NB48062–JWK0133

**Fig 7:**
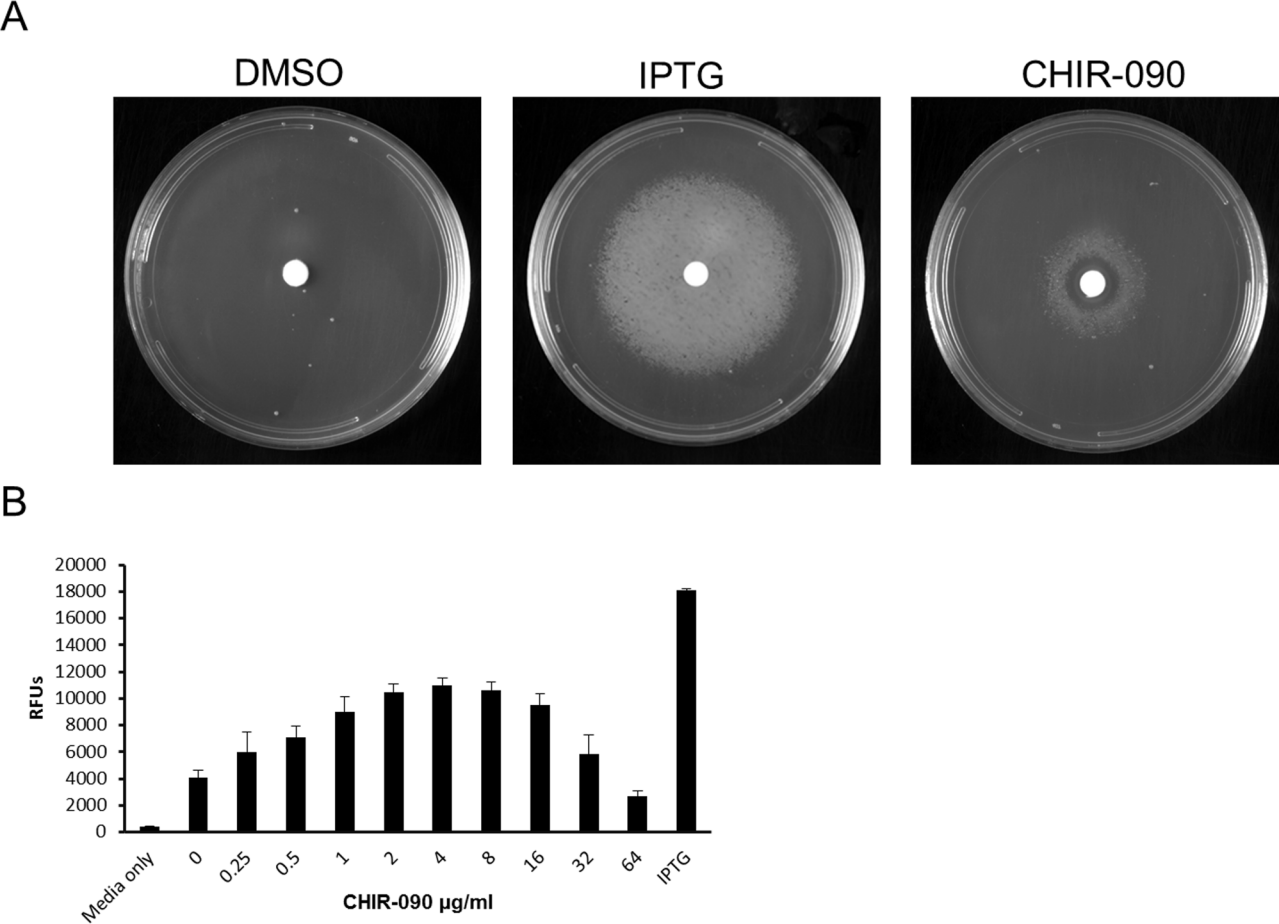
The dependence of LpxH for growth is abrogated by inhibition of LpxC under standard laboratory conditions. (A) NB48062-JWK0133 was streaked on MHIIB agar supplemented with 1 mM IPTG and grown overnight at 37°C to induce *lpxH* expression. The following day, cells were washed repeatedly and resuspended to an OD_600_ of 0.01, and 100 μL was plated on MHIIB plates without IPTG. Sterile filter discs containing IPTG, DMSO, or CHIR-090 were placed on the plates which were then incubated 37°C for 24 hours. Left panel; growth of NB48062-JWK0133 was not observed under non-inducing conditions (minus IPTG, DMSO). Center panel; growth of NB48062-JWK0133 is restored in the presence of IPTG. Right panel; NB48062-JWK0133 grew under non-inducing conditions in the presence of the LpxC inhibitor CHIR-090. (B) An overnight culture of NB48062-JWK0133 under inducing conditions (+ IPTG) was diluted to an OD_600_ of 0.1 and then was diluted 100-fold into MHIIB containing 10% Alamar Blue. Next, 100 μL of the inoculum was added to the wells of a 96-well plate containing CHIR-090 to a final assay concentrations ranging from 0.25–64 μg/ml. The plate was incubated for 6 hours at 37°C before fluorescence reading (545ex (nm)– 590em (nm)) on the SpectraMax and analyzed with Softmax® Pro software v 5.4.1.

**Fig 8:**
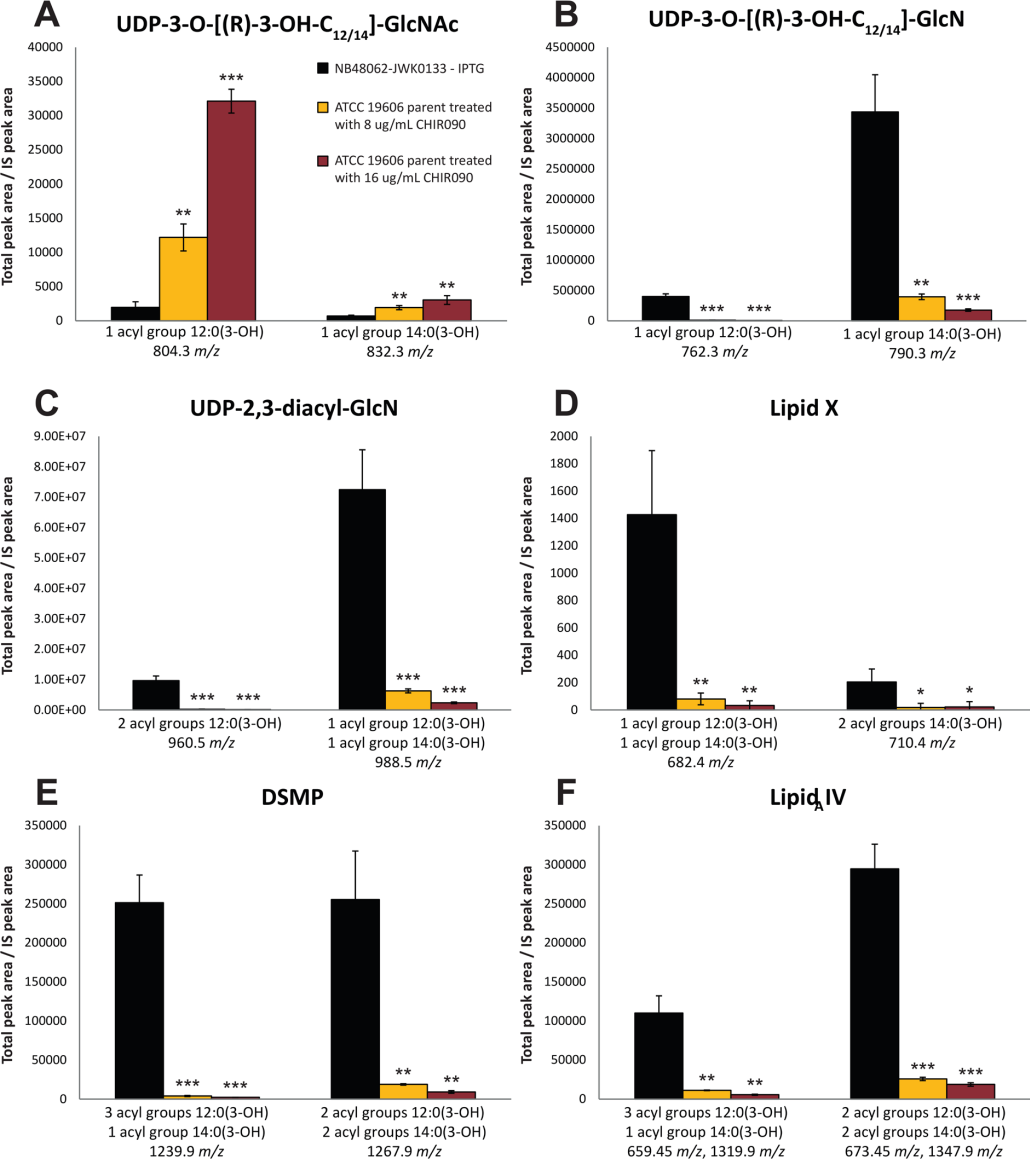
The LpxC inhibitor CHIR-090 reduces the accumulation of lipid A intermediates in NB48062-JKW0133 depletion of LpxH. The LCMS-MRM quantification of lipid A precursors from UDP-3-*O*-[(*R*)-3-OH-C_12/14_]-GlcNAc through lipid IV_A_ is shown for NB48062-JWK0133 under non-inducing conditions (-IPTG) and in the presence or absence of CHIR-090 at 8–16 μg/ml. Experiments were performed in triplicate and bars show the mean value and SD (two-tailed student t-test *, P<0.05, ** P<0.01, and ***, P<0.001 between the CHIR-090 treated cells and the uninduced cells). Data shown was normalized to an internal standard (IS) as previously described [40].

As described above, LpxH was essential for growth of *A*. *baumannii* ATCC 19606, and depletion of LpxH caused profound accumulation of detergent-like lipid A biosynthetic pathway intermediates and severe morphological changes to cells. If the failure of NB48062–JWK0133 to grow upon depletion of LpxH was mediated fully or partly by toxic accumulation of lipid A pathway intermediates, blocking the production of these intermediates by inhibiting LpxC (which can be genetically inactivated in this strain) should offset the dependence on LpxH for growth. Strongly supporting this notion, growth of LpxH depleted cells (-IPTG) was rescued by exposure to the well-characterized LpxC inhibitor, CHIR-090 [50–53] in a dose-dependent fashion (Fig 7A and 7B). The concentration of CHIR-090 used here (8–16 μg/ml) was sufficient to block LpxC since this level of exposure dramatically reduces or eliminates LPS production in *A*. *baumannii* ATCC19606 (S20 Fig). A similar result was recently reported using the LpxC inhibitor PF-508109 at 32 μg/ml [54]. Furthermore, cells under LpxH depletion conditions in the presence of CHIR-090 had much lower accumulation these pathway intermediates, indicating that the synthesis of these intermediates was blocked by CHIR-090 under these growth conditions (Fig 8A–8F). These data strongly indicate that toxic accumulation of one or more lipid A pathway intermediates underlies the dependence on LpxH for growth of *A*. *baumannii* ATCC 19606. However, the contribution of toxic accumulation upon loss of LpxH to growth impairment seen here for *A*. *baumanni* ATCC 19606 may not be universal among all Gram-negatives [26, 38]. In *E*. *coli* (where LPS *per se* is essential) depletion of LpxH (using a strain with the native *lpxH* deleted and complemented by a temperature sensitive plasmid carrying *lpxH*) caused an accumulation of the lipid A precursor UDP-diacyl-GlcN and a loss of viability under non-replicative conditions at 44°C [43]. In that case however, it is difficult to discern the direct contribution of toxic accumulation to loss of viability or inability to grow since LPS is itself essential, and early pathway genes (e.g. LpxC) cannot be inactivated or inhibited without a strong direct antibacterial effect. Conversely, in *N*. *meningitidis*, *lpxH* could be inactivated on the genome, and genomic sequencing of a LPS-deficient meningococcal isolate revealed a glycine to aspartic acid substitution (G95D) in LpxH believed to cause the loss of LPS [26]. This could mean that those cells are less sensitive to this intermediate, or alternatively that they can somehow offset accumulation, but this remains to be determined. Therefore, although our data suggests that toxic accumulation mediates, or strongly contributes to, the essentiality of LpxH for growth of *A*. *baumannii* ATCC 19606, this phenomenon remains to be fully explored for other Gram-negatives.

In conclusion, we have established that LpxH is the earliest occurring (and so far only) lipid A biosynthetic enzyme that is essential for growth of *A*. *baumannii* ATCC19606 under standard laboratory conditions. *A*. *baumannii* ATCC 19606, which can tolerate loss of LPS, provided an elegant platform to explore how toxic accumulation of LPS pathway intermediates might mediate this dependence on LpxH, in turn suggesting that other LPS biosynthetic steps downstream of LpxH could also be essential for growth. Inhibiting targets such as LpxA or LpxC, which are not essential in some *A*. *baumannii* strains, would still be expected to provide coverage in the clinic since the cells inhibited in these enzymes would lack the LPS-containing outer membrane required for virulence and drug resistance [25]. Nonetheless, this work suggests that inhibition of certain steps of the LPS pathway may have the benefit of providing outright growth prevention in strains that do not require LPS *per se*, and the contribution of toxic accumulation may extend to additional Gram-negatives making inhibition of these pathway steps particularly detrimental.

## Supporting Information

S1 Fig Insertion of *lacI*-P_tac_ upstream of *lpxH* in *A*. *baumannii* ATCC 19606. The *lacI*-P_tac_ promoter fragment was inserted in front of *lpxH* by double homologous recombination to enable IPTG controlled expression of *lpxH*.
(PDF)

S2 Fig Map of pNOV108 (NCBI (accession number: KX580601). pBR322 ori, pWH1266 P_Km_::*lacI*, *bla*, *alaS*.
(PDF)

S3 Fig Deletion of *alaS* from the chromosome in *A*. *baumannii* ATCC 19606. The *alaS* gene was replaced with a gentamicin resistance cassette (*accC1*) using double homologous recombination.
(PDF)

S4 Fig Transcription of *lpxH* is controlled by IPTG in NB48062-JWK0133. The LpxH depletion experiment was repeated as described in the manuscript. Five milliliter of culture at 0.5 OD_600_ was placed in a 15-mL conical and centrifuged at 6000 x g (4000 rpm, Sorvall) for 10 minutes to pellet cells. RNA isolation was performed using the RNeasy Mini Kit according to manufacturer’s instructions (Qiagen, 74104) and samples were diluted to a concentration of 20 ng/μL. RT-qPCR reaction were performed according to the qScriptTM XLT One-Step RT-qPCR ToughMix protocol (Quanta Biosciences, 95132–100) utilizing primer/probe set 5'-TGC TTG GAT TGG TGA TGA CTA T-3', 5'-CTC GGT TAC CCA CTT GGA AAT A-3', and 5'-/56-FAM/CGC CTT GGC/ZEN/TCG ACG AAA TTG TC/3IABkFQ/-3' (Integrated DNA Technologies). Briefly, in a 96-well PCR plate (Bio-Rad, HSP9601), 20 μL of the RT mix containing the appropriate primers and probe mixture was combined with 5 μL of the RNA sample with each reaction performed in duplicate. The plate was sealed (Bio-Rad, MSB-1001), centrifuged, and RT-qPCR reaction were monitored using the Bio-Rad CFX96 Real Time system and data analysis with Bio-Rad CFX Manager 3.1. Raw data was reformatted in Excel.
(PDF)

S5 Fig Precursor ion scan data to determine abundant acyl-chain variants for lipid IV_A_ intermediates. Precursor ion scans were run to determine the abundant acyl-chain variants for LpxC product (S5A Fig), LpxD product (S5B Fig), Lipid X (S5C Fig), DSMP (S5D Fig), and lipid IV_A_ (S5E Fig). Data are shown for both wild-type and LpxH depleted samples. The precursor ion scan mode was not sufficiently sensitive to detect LpxA product variants in this experiment, nor was data used for quantitative purposes. Mass spectra are shown for a 30 second window around the previously reported species [40]. The abundant species for LpxC and LpxD product were determined to be C12:0(3-OH) and C14:0(3-OH) based upon the specific UDP 385 *m/z* transition—consistent with previous reports [40]. Based upon these findings, all possible C12:0(3-OH) and C14:0(3-OH) variants were investigated in downstream products by monitoring the loss of phosphate using the 79 *m/z* product ion. For lipid X, the 2x C12:0(3-OH) and 1x C12:0(3-OH) / 1x C14:0(3-OH) variants were selected for analysis. For DSMP and lipid IV_A_, the 3x C12:0(3-OH) / 1x C14:0(3-OH) fatty acids were selected from previously reported species [22] and the 2x C12:0(3-OH) / 2x C14:0(3-OH) variants were selected by reason of abundance. These transitions matching the previously studied and abundant C12:0(3-OH) and C14:0(3-OH) species for all intermediates were added to the MRM transitions in S2 Table, and then quantitated across all samples [40].
(PDF)

S6 Fig QQQ LC-MRM and QQQ LC-MS/MS analysis of LpxA product`s. Chromatograms are provided for LpxA acyl chain variants from both experimental samples and authentic standards. The specific MRM transition being monitored as described in S2 Table is noted. Retention times are annotated. Peaks are labeled with (MS/MS) if product ion spectra were obtained for the specific peak. QQQ MS/MS spectra are displayed with peaks annotated. Product ion peaks are summarized in S4 Table and putative structural assignments are made in S18 Fig. In cases where a chromatographic peak is observed, a proposed structure is provided.
(PDF)

S7 Fig QTOF LC-MS and QTOF LC-MS/MS analysis of LpxA products. Chromatograms are provided for LpxA acyl chain variants from both experimental samples and authentic standards. The specific Extracted Ion Chromatogram (EIC)being monitored as described in S3 Table is noted. Retention times are annotated. Peaks are labeled with (MS/MS) if product ion spectra were obtained for the specific peak. QTOF MS/MS spectra are displayed with peaks annotated. Product ion peaks are summarized in S4 Table and putative structural assignments are made in S18 Fig. In cases where a chromatographic peak is observed, a proposed structure is provided.
(PDF)

S8 Fig QQQ LC-MRM and QQQ LC-MS/MS analysis of LpxC products. Chromatograms are provided for LpxC acyl chain variants from both experimental samples and authentic standards. The specific MRM transition being monitored as described in S2 Table is noted. Retention times are annotated. Peaks are labeled with (MS/MS) if product ion spectra were obtained for the specific peak. QQQ MS/MS spectra are displayed with peaks annotated. Product ion peaks are summarized in S4 Table and putative structural assignments are made in S18 Fig. In cases where a chromatographic peak is observed, a proposed structure is provided.
(PDF)

S9 Fig QTOF LC-MS and QTOF LC-MS/MS analysis of LpxC products. Chromatograms are provided for LpxC acyl chain variants from both experimental samples and authentic standards. The specific Extracted Ion Chromatogram (EIC) being monitored as described in S3 Table is noted. Retention times are annotated. Peaks are labeled with (MS/MS) if product ion spectra were obtained for the specific peak. QTOF MS/MS spectra are displayed with peaks annotated. Product ion peaks are summarized in S4 Table and putative structural assignments are made in S18 Fig. In cases where a chromatographic peak is observed, a proposed structure is provided.
(PDF)

S10 Fig QQQ LC-MRM and QQQ LC-MS/MS analysis of LpxD products. Chromatograms are provided for LpxD acyl chain variants from both experimental samples and authentic standards. The specific MRM transition being monitored as described in S2 Table is noted. Retention times are annotated. Peaks are labeled with (MS/MS) if product ion spectra were obtained for the specific peak. QQQ MS/MS spectra are displayed with peaks annotated. Product ion peaks are summarized in S4 Table and putative structural assignments are made in S18 Fig. In cases where a chromatographic peak is observed a proposed structure is provided. Acyl chain positions are for illustrative purposes only, based upon the final Lipid A structure. Our analysis does not clarify whether the species is C_12_ / C_14_, C_14_/ C_12_, or a mixture of these.
(PDF)

S11 Fig QTOF LC-MS and QTOF LC-MS/MS analysis of LpxD products. Chromatograms are provided for LpxD acyl chain variants from both experimental samples and authentic standards. The specific Extracted Ion Chromatogram (EIC) being monitored as described in S3 Table is noted. Retention times are annotated. Peaks are labeled with (MS/MS) if product ion spectra were obtained for the specific peak. QTOF MS/MS spectra are displayed with peaks annotated. Product ion peaks are summarized in S4 Table and putative structural assignments are made in S18 Fig. In cases where a chromatographic peak is observed a proposed structure is provided. Acyl chain positions are for illustrative purposes only, based upon the final Lipid A structure. Our analysis does not clarify whether the species is C_12_ / C_14_, C_14_/ C_12_, or a mixture of these.
(PDF)

S12 Fig QQQ LC-MRM and QQQ LC-MS/MS analysis of Lipid X. Chromatograms are provided for Lipid X acyl chain variants from both experimental samples and authentic standards. The specific MRM transition being monitored as described in S2 Table is noted. Retention times are annotated. Peaks are labeled with (MS/MS) if product ion spectra were obtained for the specific peak. QQQ MS/MS spectra are displayed with peaks annotated. Product ion peaks are summarized in S4 Table and putative structural assignments are made in S18 Fig. In cases where a chromatographic peak is observed a proposed structure is provided. Acyl chain positions are for illustrative purposes only, based upon the final Lipid A structure. Our analysis does not clarify whether the species is C_12_ / C_14_, C_14_/ C_12_, or a mixture of these.
(PDF)

S13 Fig QTOF LC-MS and QTOF LC-MS/MS analysis of Lipid X. Chromatograms are provided for Lipid X acyl chain variants from both experimental samples and authentic standards. The specific Extracted Ion Chromatogram (EIC) being monitored as described in S3 Table is noted. Retention times are annotated. Peaks are labeled with (MS/MS) if product ion spectra were obtained for the specific peak. QTOF MS/MS spectra are displayed with peaks annotated. Product ion peaks are summarized in S4 Table and putative structural assignments are made in S18 Fig. In cases where a chromatographic peak is observed a proposed structure is provided. Acyl chain positions are for illustrative purposes only, based upon the final Lipid A structure. Our analysis does not clarify whether the species is C_12_ / C_14_, C_14_/ C_12_, or a mixture of these.
(PDF)

S14 Fig QQQ LC-MRM and QQQ LC-MS/MS analysis of DSMP. Chromatograms are provided for DSMP acyl chain variants from both experimental samples and authentic standards. The specific MRM transition being monitored as described in S2 Table is noted. Retention times are annotated. Peaks are labeled with (MS/MS) if product ion spectra were obtained for the specific peak. QQQ MS/MS spectra are displayed with peaks annotated. Product ion peaks are summarized in S4 Table and putative structural assignments are made in S18 Fig. In cases where a chromatographic peak is observed a proposed structure is provided. Acyl chain positions are for illustrative purposes only, based upon the final Lipid A structure. Our analysis does not clarify whether the species is C_12_ / C_14_ / C_12_ / C_14_ or C_12_ / C_12_ / C_14_ / C_14_, or a mixture of these.
(PDF)

S15 Fig QTOF LC-MS and QTOF LC-MS/MS analysis of DSMP. Chromatograms are provided for DSMP acyl chain variants from both experimental samples and authentic standards. The specific Extracted Ion Chromatogram (EIC) being monitored as described in S3 Table is noted. Retention times are annotated. Peaks are labeled with (MS/MS) if product ion spectra were obtained for the specific peak. QTOF MS/MS spectra are displayed with peaks annotated. Product ion peaks are summarized in S4 Table and putative structural assignments are made in S18 Fig. In cases where a chromatographic peak is observed a proposed structure is provided. Acyl chain positions are for illustrative purposes only, based upon the final Lipid A structure. Our analysis does not clarify whether the species is C_12_ / C_14_ / C_12_ / C_14_ or C_12_ / C_12_ / C_14_ / C_14_, or a mixture of these.
(PDF)

S16 Fig QQQ LC-MRM and QQQ LC-MS/MS analysis of lipid IV_A_. Chromatograms are provided for Lipid IVa acyl chain variants from both experimental samples and authentic standards. The specific MRM transition being monitored as described in S2 Table is noted. Retention times are annotated. Peaks are labeled with (MS/MS) if product ion spectra were obtained for the specific peak. QQQ MS/MS spectra are displayed with peaks annotated. Product ion peaks are summarized in S4 Table and putative structural assignments are made in S18 Fig. In cases where a chromatographic peak is observed a proposed structure is provided. Acyl chain positions are for illustrative purposes only, based upon the final Lipid A structure. Our analysis does not clarify whether the species is C_12_ / C_14_ / C_12_ / C_14_ or C_12_ / C_12_ / C_14_ / C_14_, or a mixture of these.
(PDF)

S17 Fig QTOF LC-MS and QTOF LC-MS/MS analysis of lipid IV_A_. Chromatograms are provided for Lipid IVa acyl chain variants from both experimental samples and authentic standards. The specific Extracted Ion Chromatogram (EIC) being monitored as described in S3 Table is noted. Retention times are annotated. Peaks are labeled with (MS/MS) if product ion spectra were obtained for the specific peak. QTOF MS/MS spectra are displayed with peaks annotated. Product ion peaks are summarized in S4 Table and putative structural assignments are made in S18 Fig. In cases where a chromatographic peak is observed a proposed structure is provided. Acyl chain positions are for illustrative purposes only, based upon the final Lipid A structure. Our analysis does not clarify whether the species is C_12_ / C_14_ / C_12_ / C_14_ or C_12_ / C_12_ / C_14_ / C_14_, or a mixture of these.
(PDF)

S18 Fig Proposed MS/MS product ion fragments for LPS intermediates. Proposed species are provided, consistent with reported MS/MS fragmentation for LPS intermediates and observed product ions. Species are drawn as neutral molecules with exact mass and *m/z* in the 1^-^ charge state displayed. Acyl chain positions are for illustrative purposes only, based upon the final Lipid A structure. For example, it is not known if the species is C_12_ / C_14_ / C_12_ / C_14_ or C_12_ / C_12_ / C_14_ / C_14_, or a mixture from our analysis. See S4 Table for ions of interest.
(PDF)

S19 Fig LpxA product, lipid X, and lipid IV_A_ intermediate levels. (A) LCMS-MRM quantification of LpxA product with 1 acyl group 12:0(3-OH) and 1 acyl group 14:0(3-OH) is shown for *A*. *baumannii* ATCC 19606 parent and NB48062-JWK0133 under inducing and non-inducing conditions. (B) LCMS-MRM quantification of lipid X with 1 acyl group 12:0(3-OH) and 1 acyl group 14:0(3-OH) compared with 2 acyl groups 14:0(3-OH) is shown for *A*. *baumannii* ATCC 19606 parent and NB48062-JWK0133 under inducing and non-inducing conditions. (C) Lipid IV_A_ with three acyl groups with 12:0(3-OH) and one acyl group of 14:0(3-OH), compared with two acyl groups 12:0(3-OH) and two acyl groups 14:0(3-OH) for *A*. *baumannii* ATCC 19606 parent and NB48062-JWK0133 under inducing and non-inducing conditions. Experiments were performed in triplicate and bars show the mean value and SD) between NB46082-JWK0133 in the presence or absence of IPTG. Data shown was normalized to an internal standard (IS) as previously described [40].
(PDF)

S20 Fig CHIR-090 inhibits synthesis of LPS in *A*. *baumannii* ATCC 19606. Lane 1, *E*. *coli* LPS standard; Lane 2, *A*. *baumannii* ATCC 19606 parent; Lane 3, *A*. *baumannii* ATCC 19606 *+* 8 μg/ml CHIR-090. Experiments were performed as previously described [40].
(PDF)

S1 File Supplementary Methods. (DOCX)

S1 Table Oligonucleotide primers used in this study. (PDF)

S2 Table Multiple Reaction Monitoring (MRM) transition table for lipid A intermediates. Lipid A pathway intermediates are described with acyl chain variants. Pathway enzymes producing each intermediate are also listed. Charge of the targeted transition, precursor ions, product ions, and collision energies are noted.
(PDF)

S3 Table LC analysis of lipid A intermediates from authentic standards and experimental samples. Lipid A pathway intermediates are described with acyl chain variants. Retention times by QQQ MRM analysis are noted, with standard peaks within 0.2 minutes of experimental peaks. Differences may be due to matrix effects for the poorly behaved detergent like molecules. QTOF theoretical and experimental monoisotopic m/z values are reported with mass errors in parts per million (ppm). Mass errors are <5 ppm for all species detected in the QTOF. Retention times by QTOF LC-MS are noted, with standard peaks within 0.2 minutes of experimental peaks. Differences may be due to matrix effects for the poorly behaved detergent like molecules. Peak times differ between QTOF and QQQ systems due to substantially larger dead-volume in the QTOF system. The relatively poor behavior observed for lipid IV_A_ in the QTOF system may also relate to interaction with the plastic surfaces in our Agilent system. Our AB Sciex system uses all glass and metal surfaces post-column.
(PDF)

S4 Table MS/MS analysis of lipid A intermediates from authentic standards and experimental samples. Lipid A pathway intermediates are described. QQQ precursor *m/z* or QTOF theoretical *m/z* are provided depending on where the specific spectra was acquired—this is also noted in the reported precision. Product ions of interest are indicated and if they can be assigned to a putative structure as in S18 Fig, a theoretical exact *m/z* is provided. Peaks are also noted if a) shared between acyl chain variants (e.g. UDP fragments of LpxD product) or b) shifted by 28 Da between acyl chain variants (e.g. acyl-GlcN fragments form LpxD product) with the second class in bold. Peaks matching a specific MRM transition are italic underlined as reported in S4 Table. MRM transitions were determined by analysis of authentic standards, however, not all MRM transitions were apparent as product ion spectra were collected at a single collision energy rather than by the varied collision energies used in the initial MRM (S4 Table).
(PDF)
