# Supplementary material for: Toxic Accumulation of LPS Pathway Intermediates Underlies the Requirement of LpxH for Growth of Acinetobacter baumannii ATCC 19606
Source: PLoS One. 2016 Aug 15;11(8):e0160918. doi: 10.1371/journal.pone.0160918 (PMC4985137; doi:10.1371/journal.pone.0160918)
Supplement: S1 Fig — The lacI-Ptac promoter fragment was inserted in front of lpxH by double homologous recombination to enable IPTG controlled expression of lpxH. (PDF) [file pone.0160918.s001.pdf]

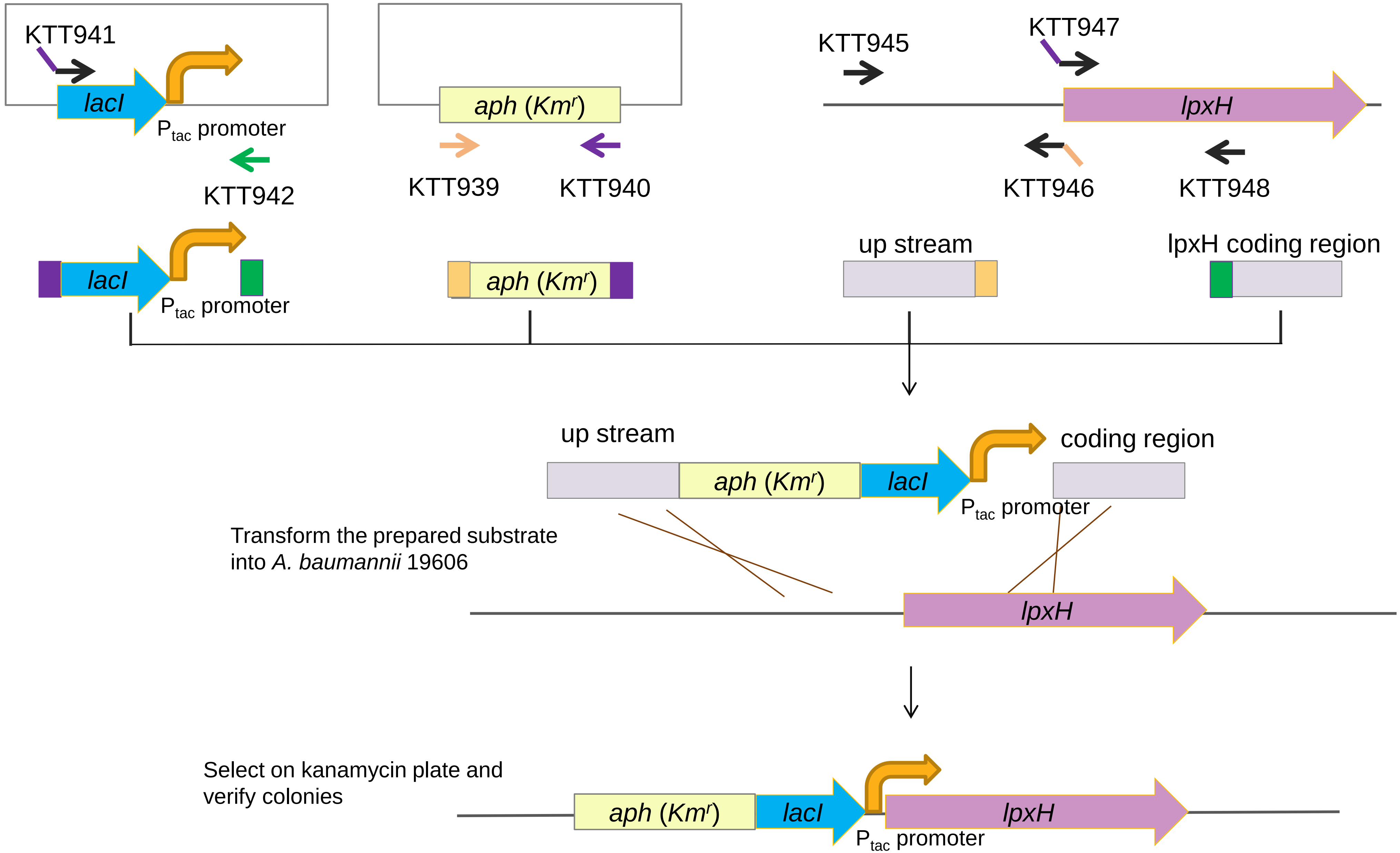

KTT941
lacI
Ptac promoter
KTT942
aph (Km^r)
KTT939
KTT940
KTT945
KTT947
lpxH
KTT946
KTT948
lacI
Ptac promoter
aph (Km^r)
up stream
lpxH coding region
up stream
aph (Km^r)
lacI
Ptac promoter
coding region
Transform the prepared substrate
into A. baumannii 19606
lpxH
Select on kanamycin plate and
verify colonies
aph (Km^r)
lacI
Ptac promoter
lpxH
