# Supplementary figures and images for: Toxic Accumulation of LPS Pathway Intermediates Underlies the Requirement of LpxH for Growth of Acinetobacter baumannii ATCC 19606
Source: PLoS One. 2016 Aug 15;11(8):e0160918. doi: 10.1371/journal.pone.0160918 (PMC4985137; doi:10.1371/journal.pone.0160918)

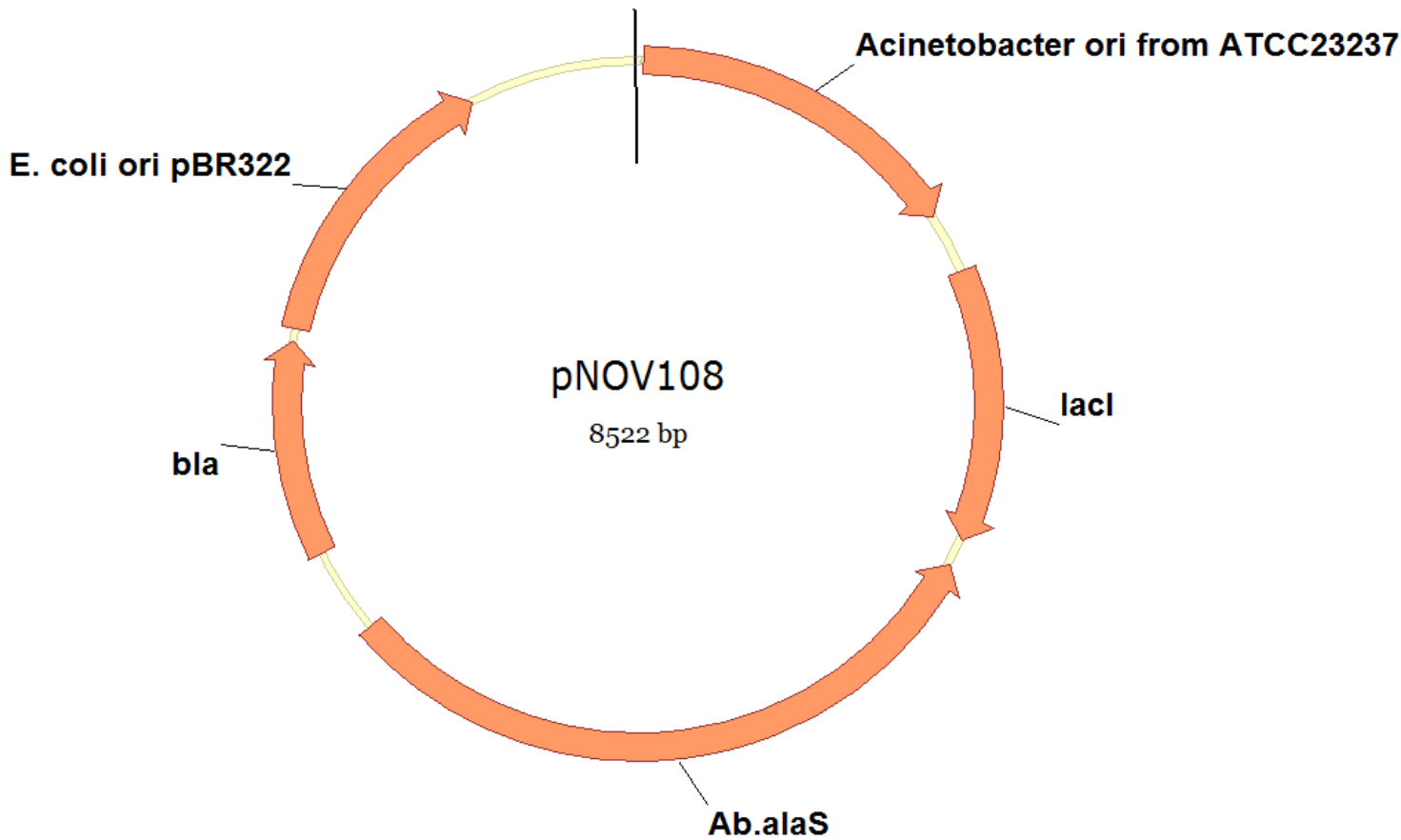

Supplement: S2 Fig — pBR322 ori, pWH1266 PKm::lacI, bla, alaS. (PDF) [file pone.0160918.s002.pdf]

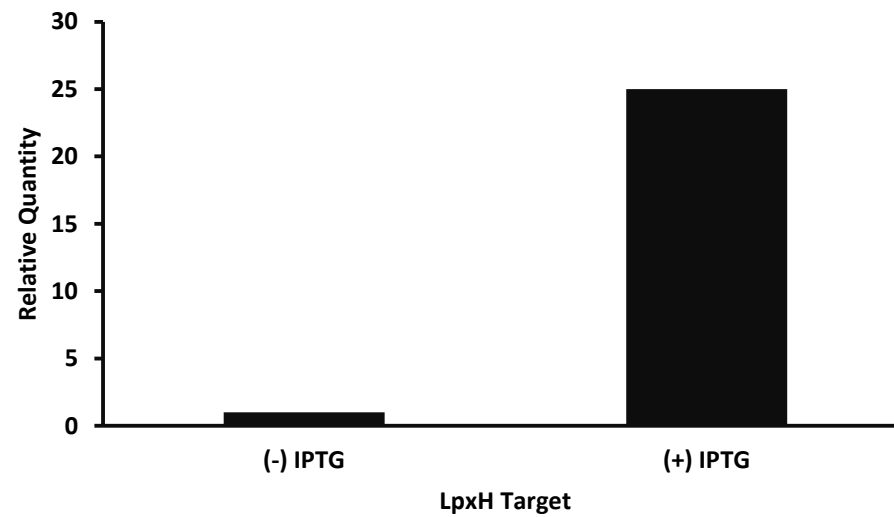

Supplement: S4 Fig — The LpxH depletion experiment was repeated as described in the manuscript. Five milliliter of culture at 0.5 OD600 was placed in a 15-mL conical and centrifuged at 6000 x g (4000 rpm, Sorvall) for 10 minutes to pellet cells. RNA isolation was performed using the RNeasy Mini Kit according to manufacturer’s instructions (Qiagen, 74104) and samples were diluted to a concentration of 20 ng/μL. RT-qPCR reaction were performed according to the qScriptTM XLT One-Step RT-qPCR ToughMix protocol (Quanta Biosciences, 95132–100) utilizing primer/probe set 5'-TGC TTG GAT TGG TGA TGA CTA T-3', 5'-CTC GGT TAC CCA CTT GGA AAT A-3', and 5'-/56-FAM/CGC CTT GGC/ZEN/TCG ACG AAA TTG TC/3IABkFQ/-3' (Integrated DNA Technologies). Briefly, in a 96-well PCR plate (Bio-Rad, HSP9601), 20 μL of the RT mix containing the appropriate primers and probe mixture was combined with 5 μL of the RNA sample with each reaction performed in duplicate. The plate was sealed (Bio-Rad, MSB-1001), centrifuged, and RT-qPCR reaction were monitored using the Bio-Rad CFX96 Real Time system and data analysis with Bio-Rad CFX Manager 3.1. Raw data was reformatted in Excel. (PDF) [file pone.0160918.s004.pdf]

A.

Intensity (arb.)

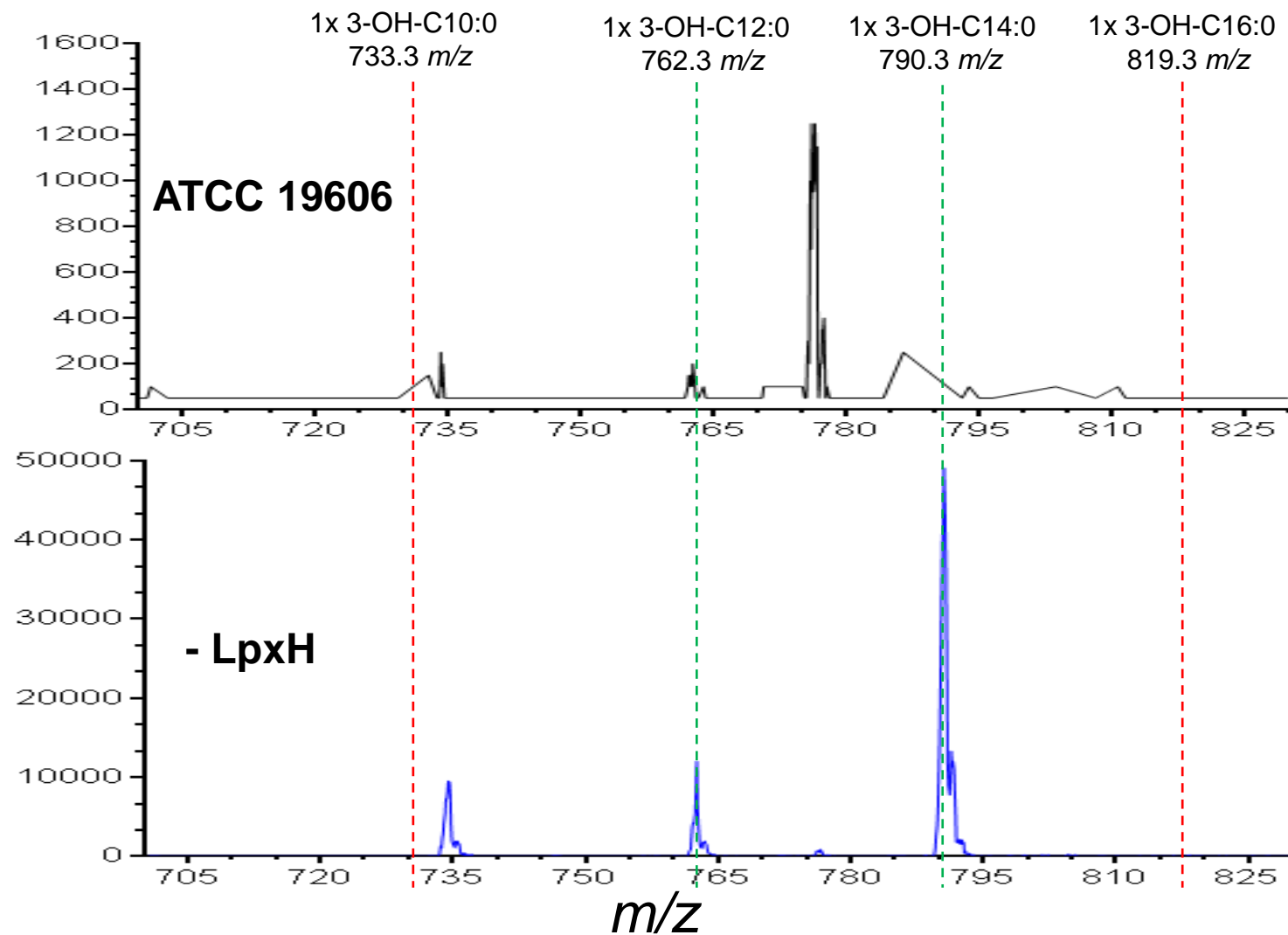

B.

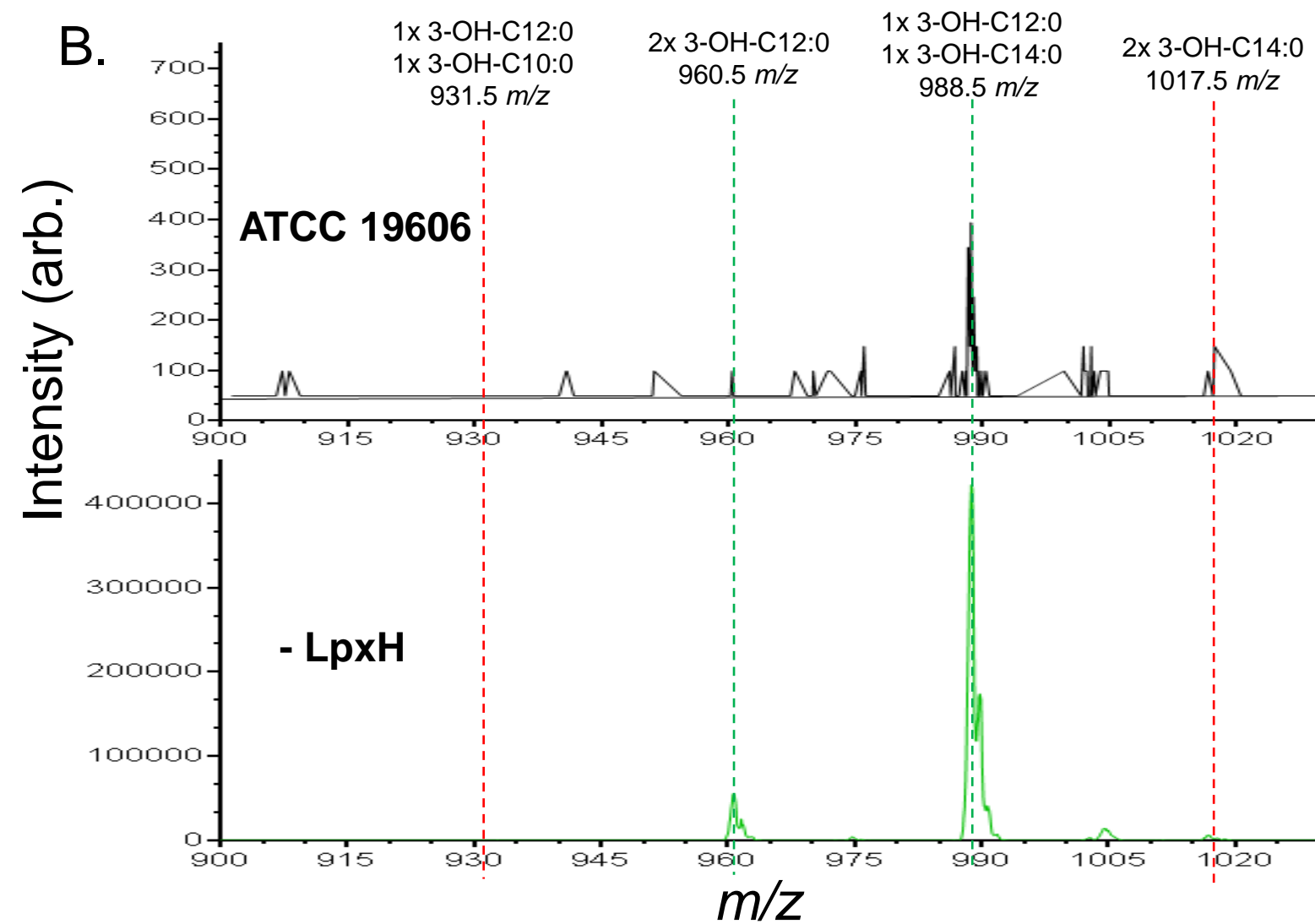

C.

Intensity (arb.)

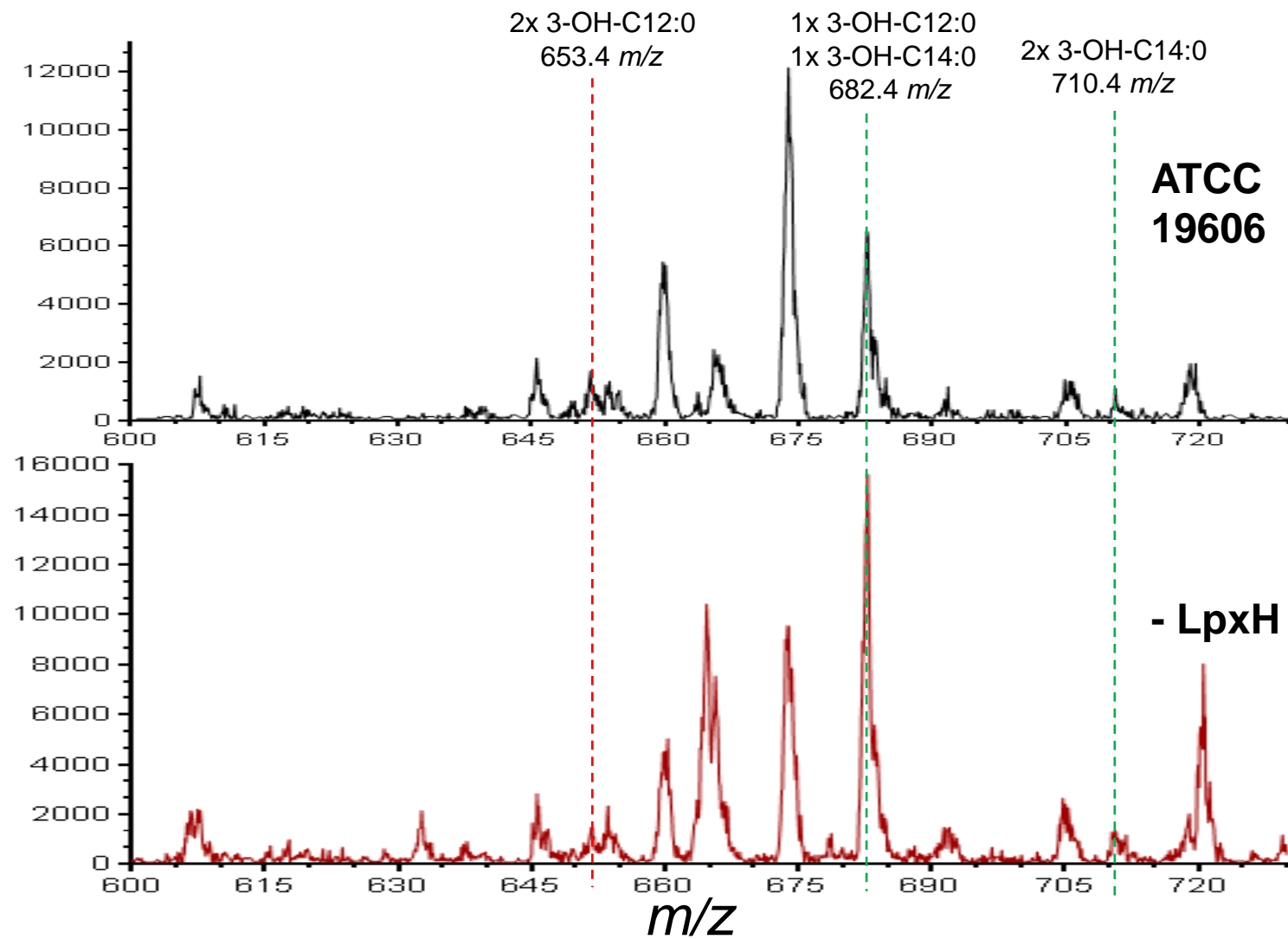

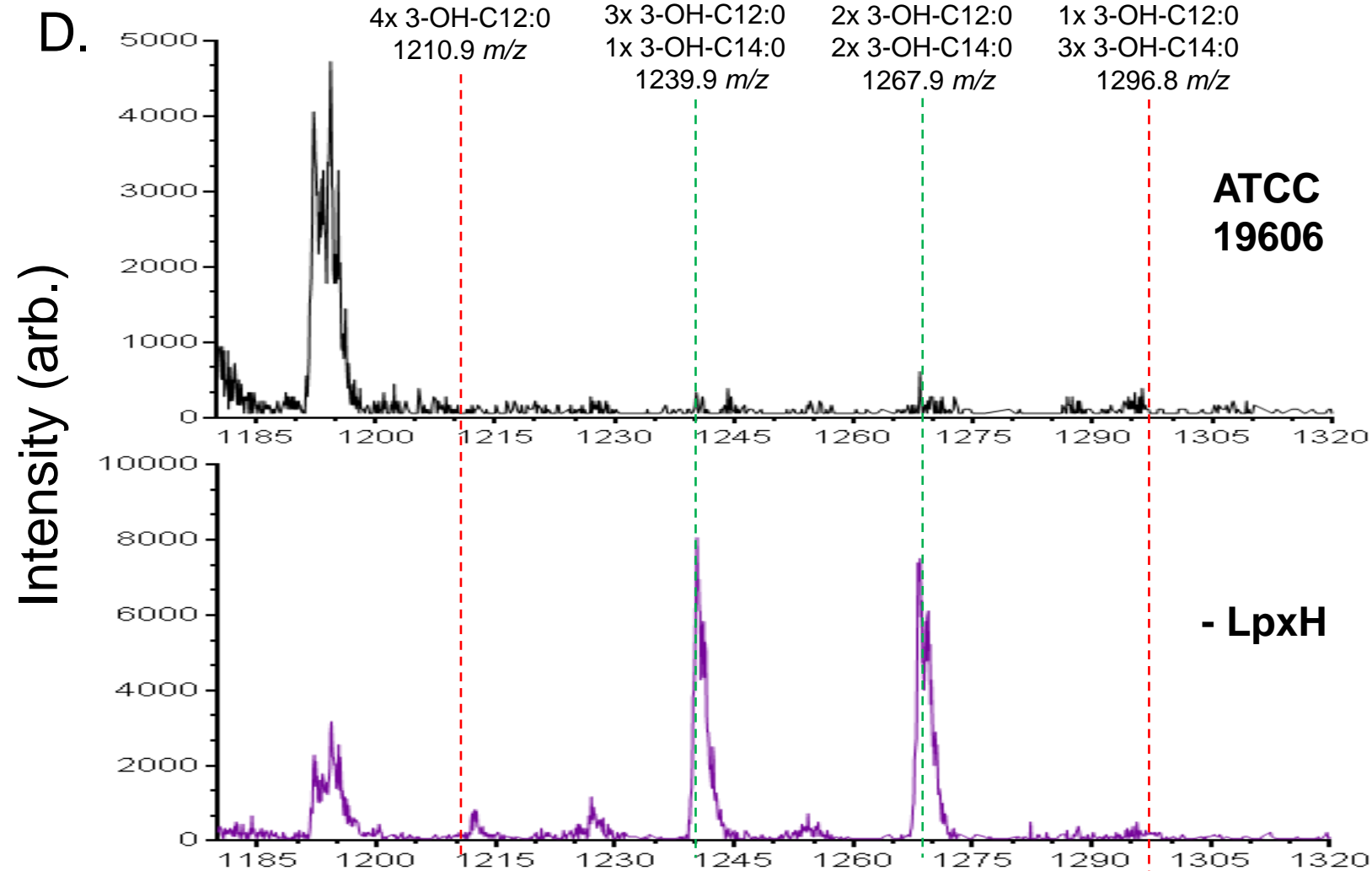

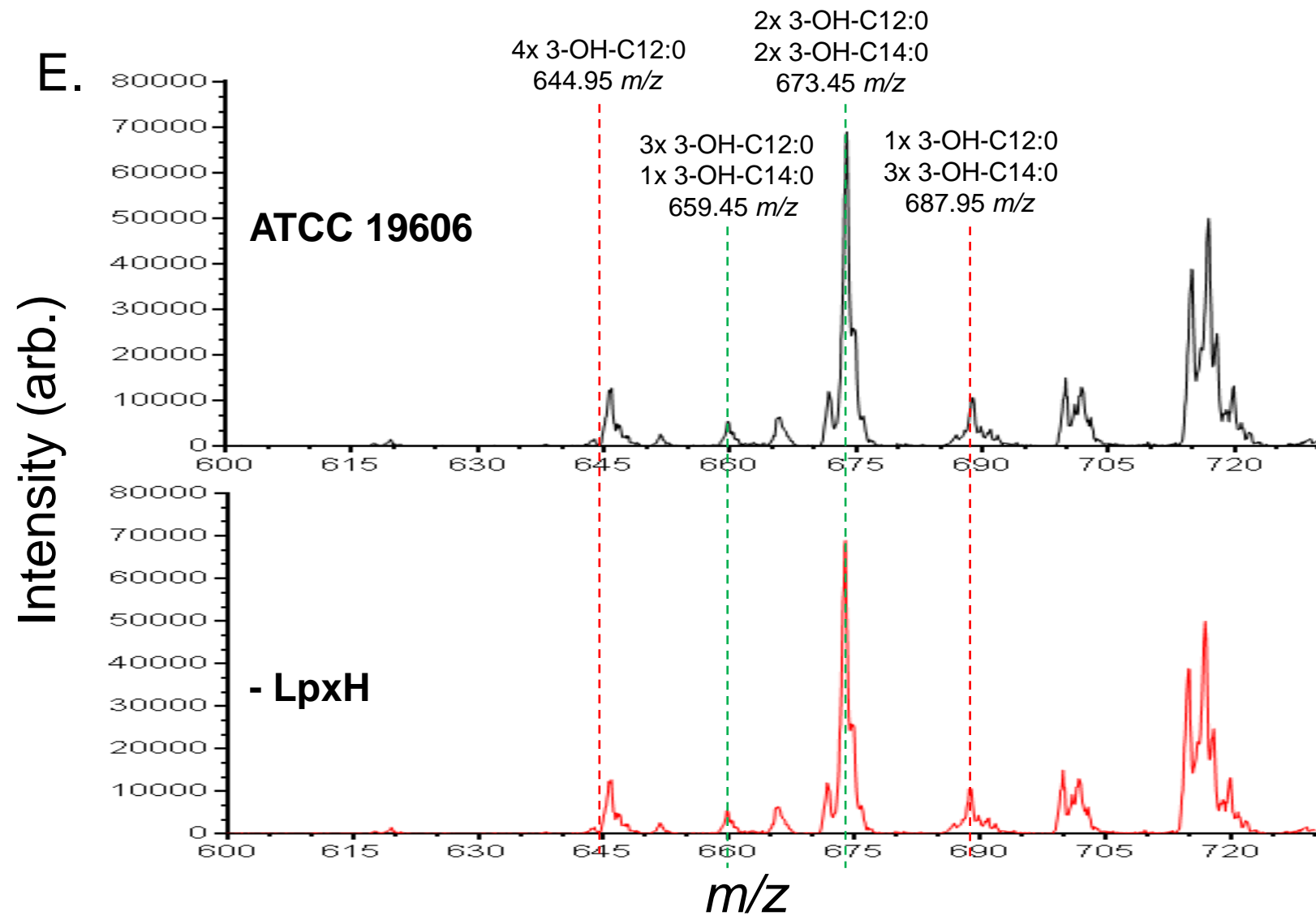

Supplement: S5 Fig — Precursor ion scans were run to determine the abundant acyl-chain variants for LpxC product (S5A Fig), LpxD product (S5B Fig), Lipid X (S5C Fig), DSMP (S5D Fig), and lipid IVA (S5E Fig). Data are shown for both wild-type and LpxH depleted samples. The precursor ion scan mode was not sufficiently sensitive to detect LpxA product variants in this experiment, nor was data used for quantitative purposes. Mass spectra are shown for a 30 second window around the previously reported species [40]. The abundant species for LpxC and LpxD product were determined to be C12:0(3-OH) and C14:0(3-OH) based upon the specific UDP 385 m/z transition—consistent with previous reports [40]. Based upon these findings, all possible C12:0(3-OH) and C14:0(3-OH) variants were investigated in downstream products by monitoring the loss of phosphate using the 79 m/z product ion. For lipid X, the 2x C12:0(3-OH) and 1x C12:0(3-OH) / 1x C14:0(3-OH) variants were selected for analysis. For DSMP and lipid IVA, the 3x C12:0(3-OH) / 1x C14:0(3-OH) fatty acids were selected from previously reported species [22] and the 2x C12:0(3-OH) / 2x C14:0(3-OH) variants were selected by reason of abundance. These transitions matching the previously studied and abundant C12:0(3-OH) and C14:0(3-OH) species for all intermediates were added to the MRM transitions in S2 Table, and then quantitated across all samples [40]. (PDF) [file pone.0160918.s005.pdf]

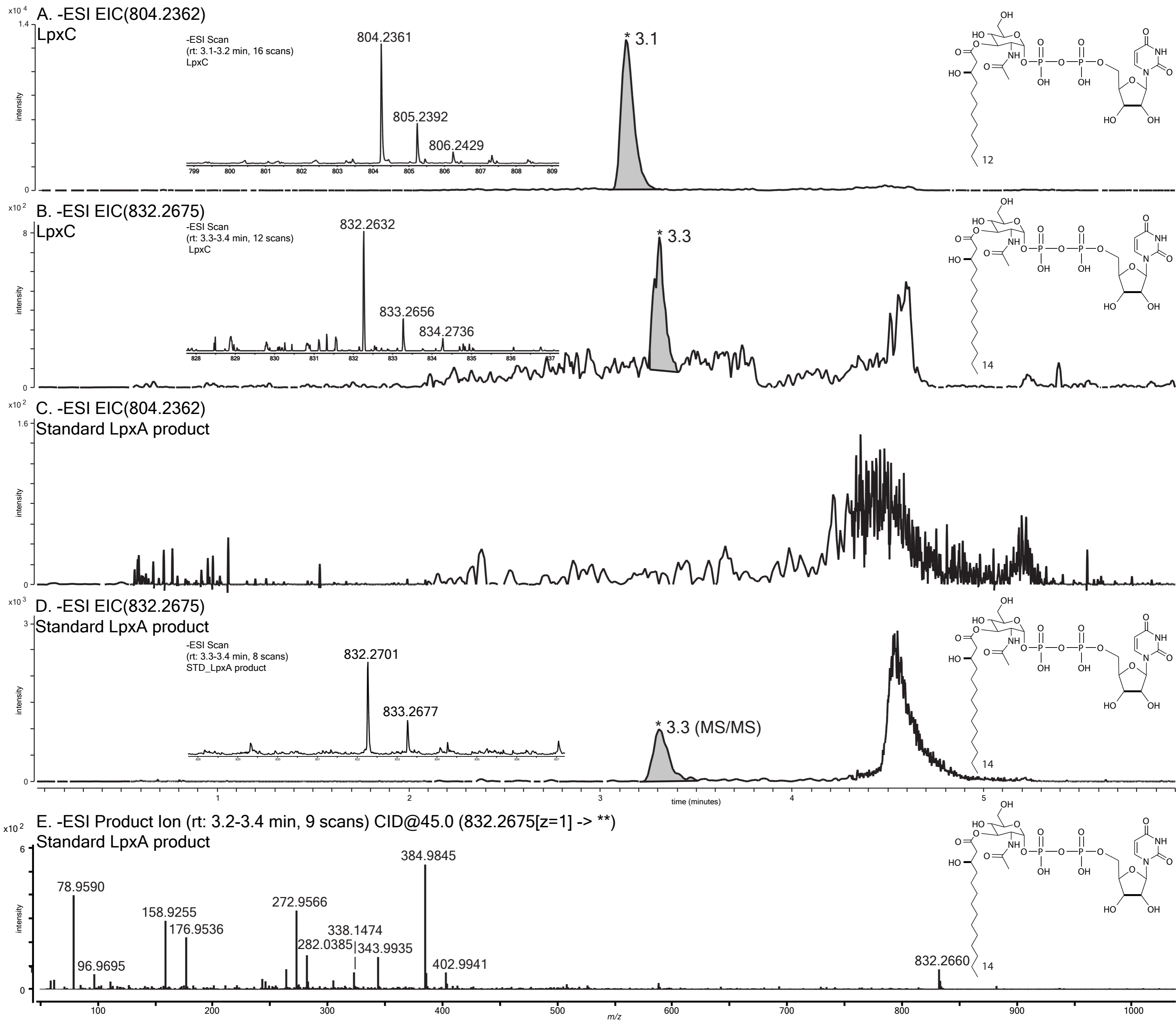

Supplement: S7 Fig — Chromatograms are provided for LpxA acyl chain variants from both experimental samples and authentic standards. The specific Extracted Ion Chromatogram (EIC)being monitored as described in S3 Table is noted. Retention times are annotated. Peaks are labeled with (MS/MS) if product ion spectra were obtained for the specific peak. QTOF MS/MS spectra are displayed with peaks annotated. Product ion peaks are summarized in S4 Table and putative structural assignments are made in S18 Fig. In cases where a chromatographic peak is observed, a proposed structure is provided. (PDF) [file pone.0160918.s007.pdf]

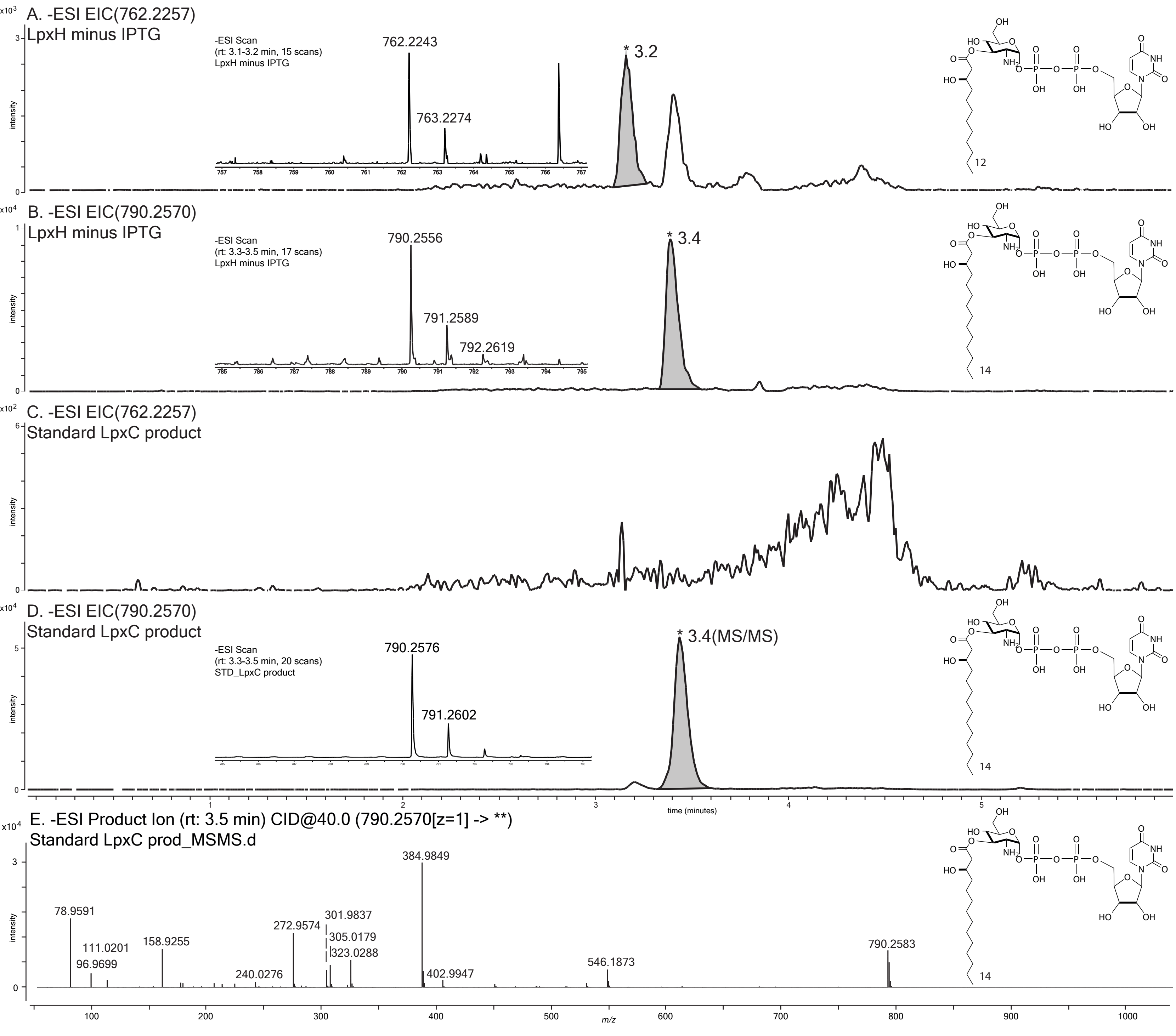

Supplement: S9 Fig — Chromatograms are provided for LpxC acyl chain variants from both experimental samples and authentic standards. The specific Extracted Ion Chromatogram (EIC) being monitored as described in S3 Table is noted. Retention times are annotated. Peaks are labeled with (MS/MS) if product ion spectra were obtained for the specific peak. QTOF MS/MS spectra are displayed with peaks annotated. Product ion peaks are summarized in S4 Table and putative structural assignments are made in S18 Fig. In cases where a chromatographic peak is observed, a proposed structure is provided. (PDF) [file pone.0160918.s009.pdf]

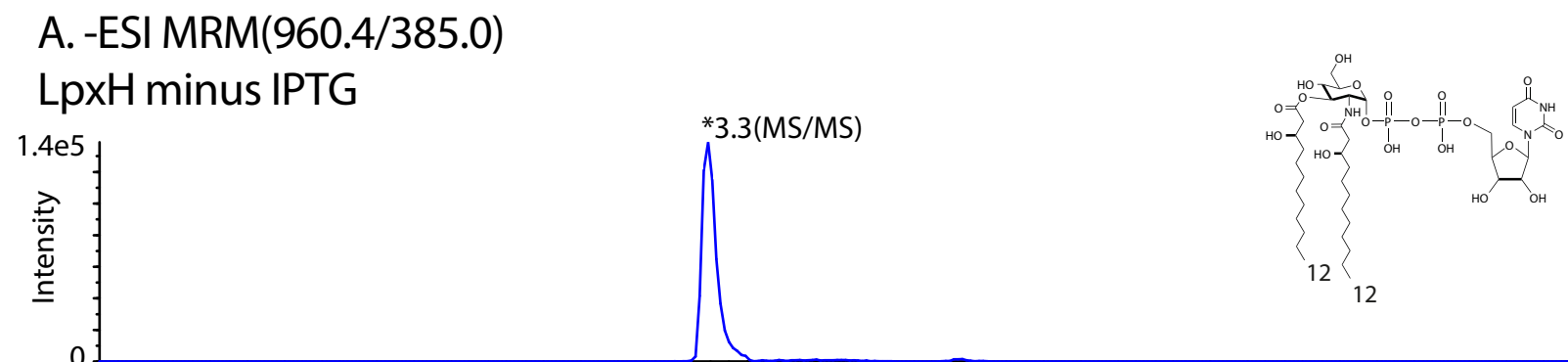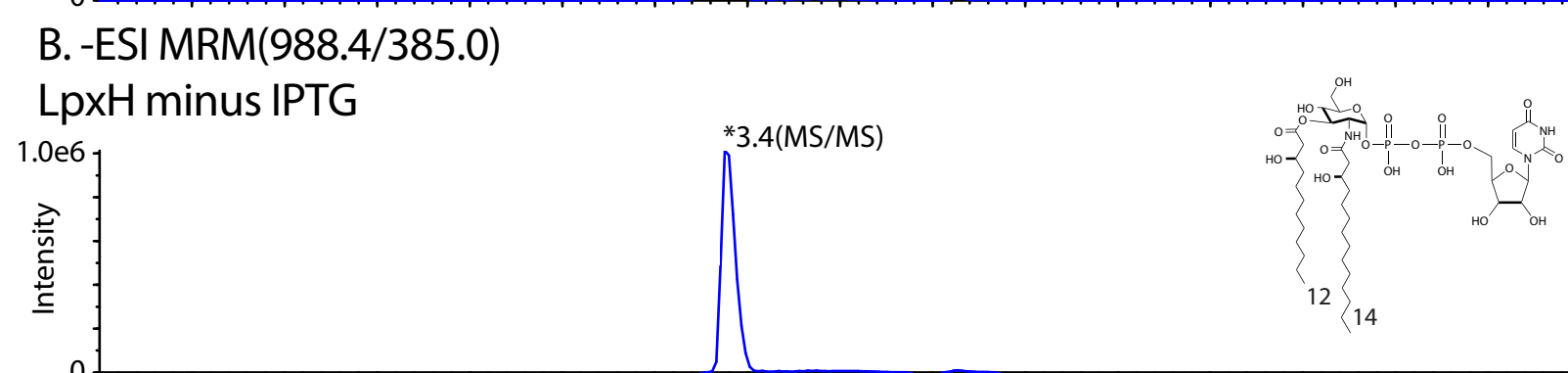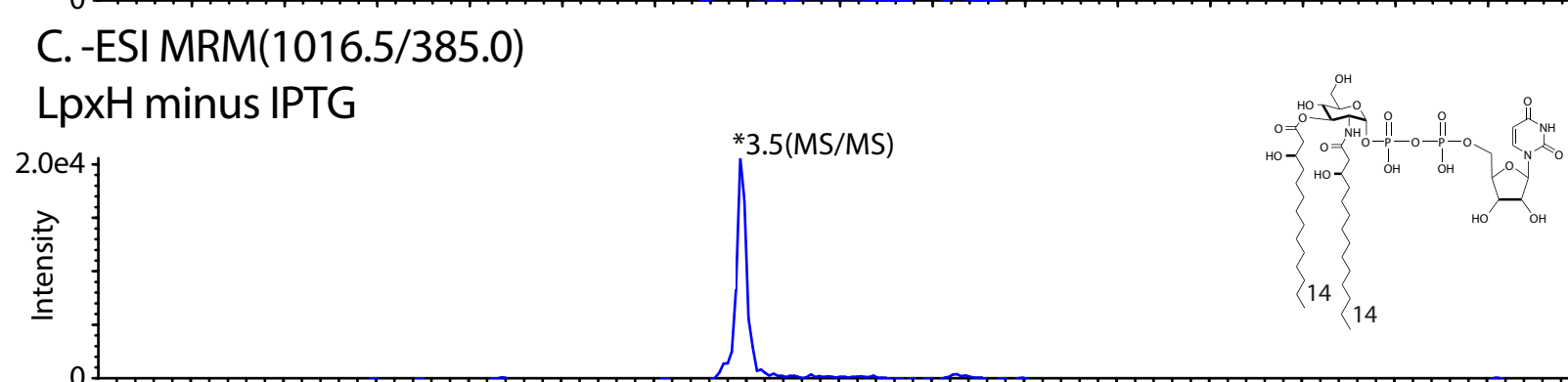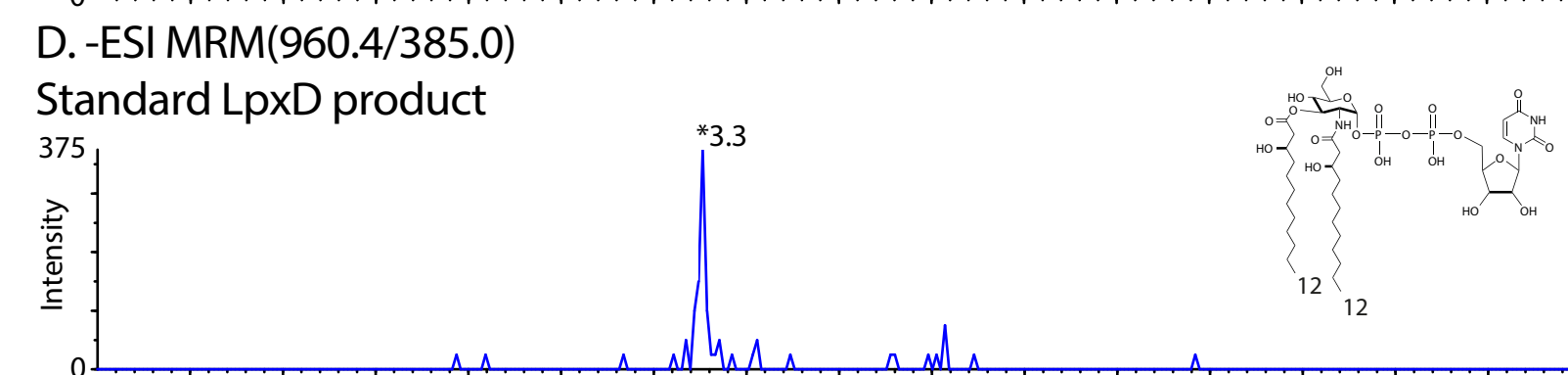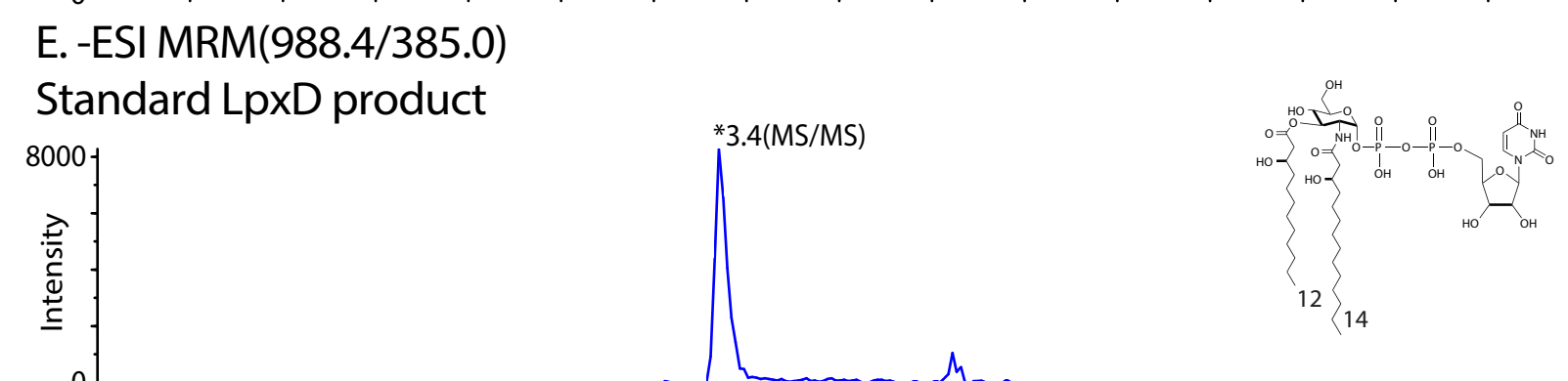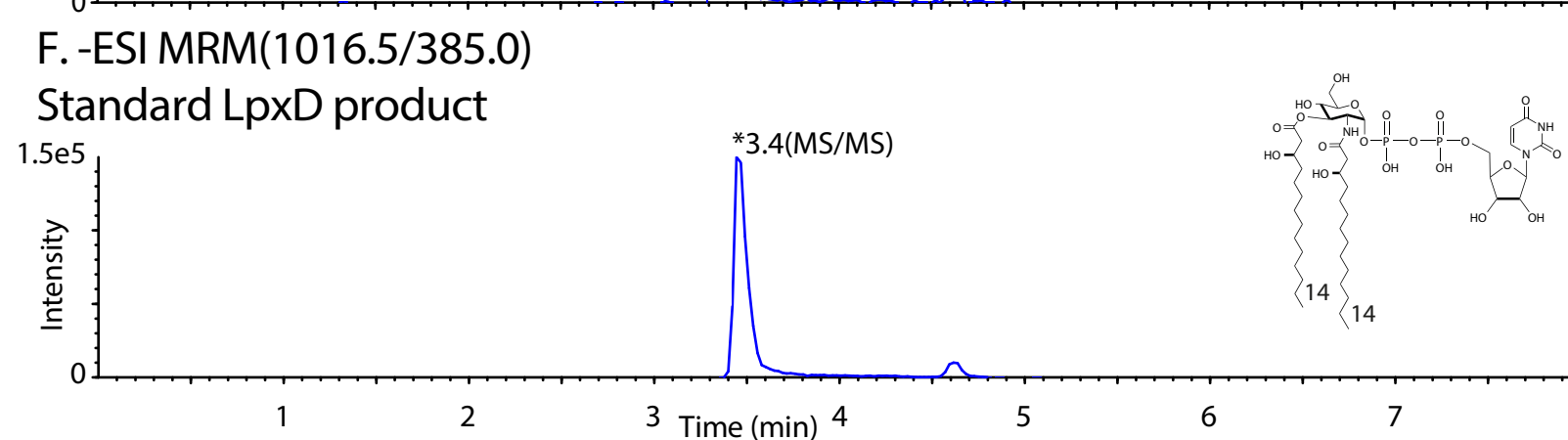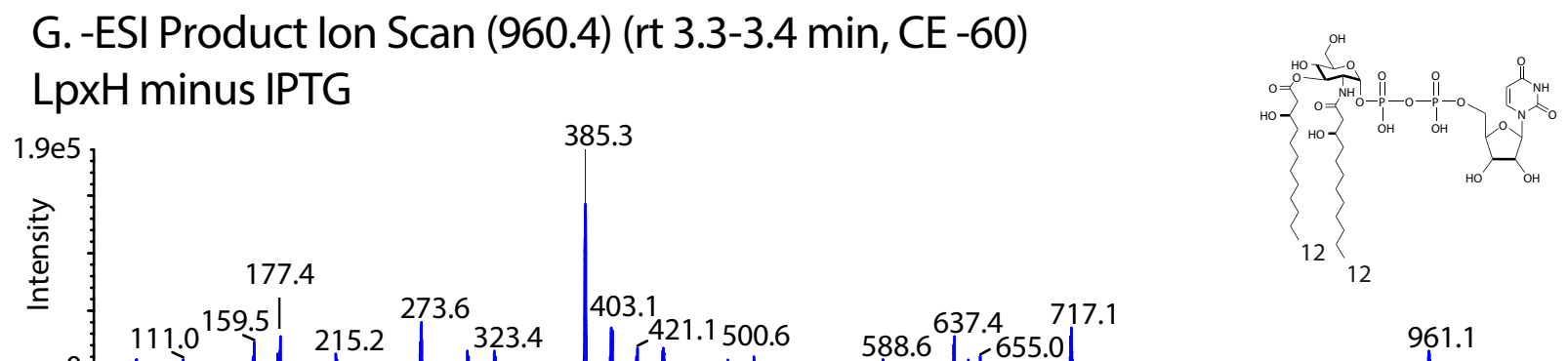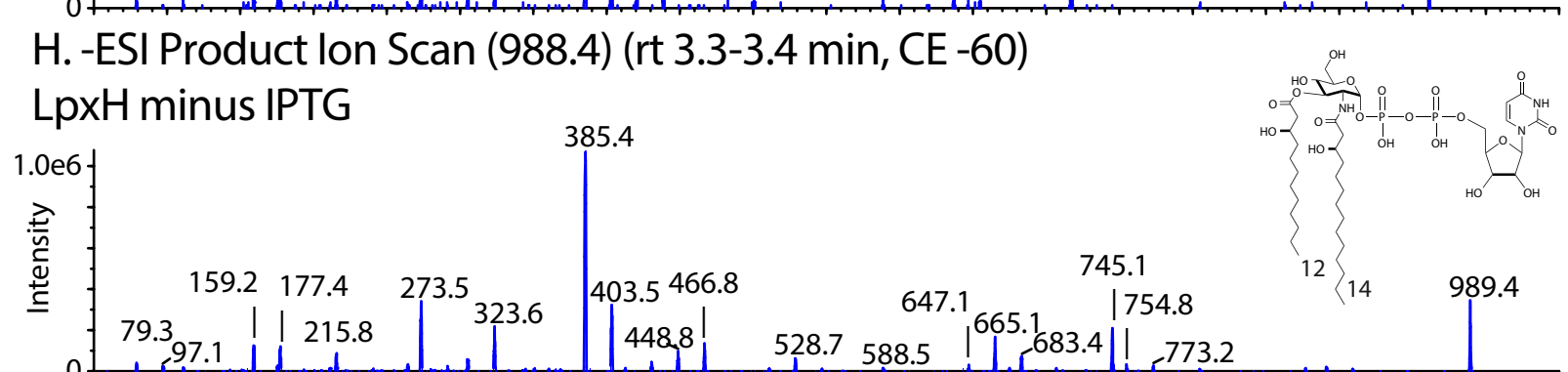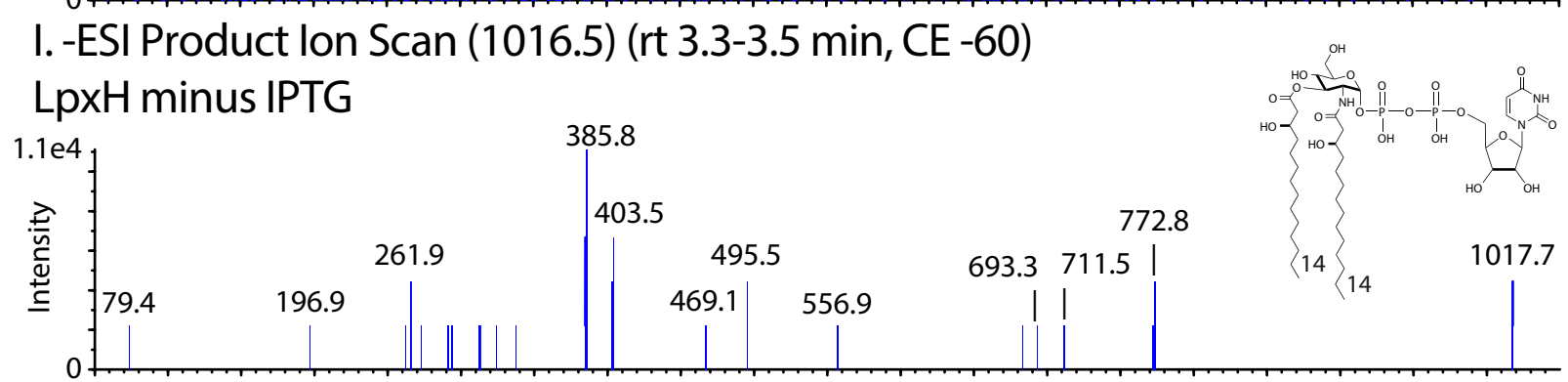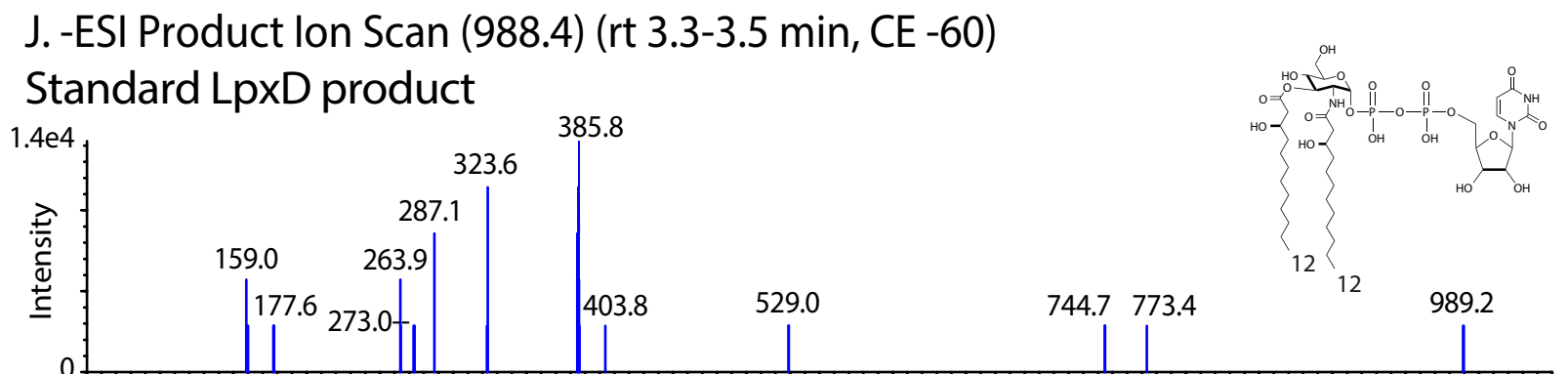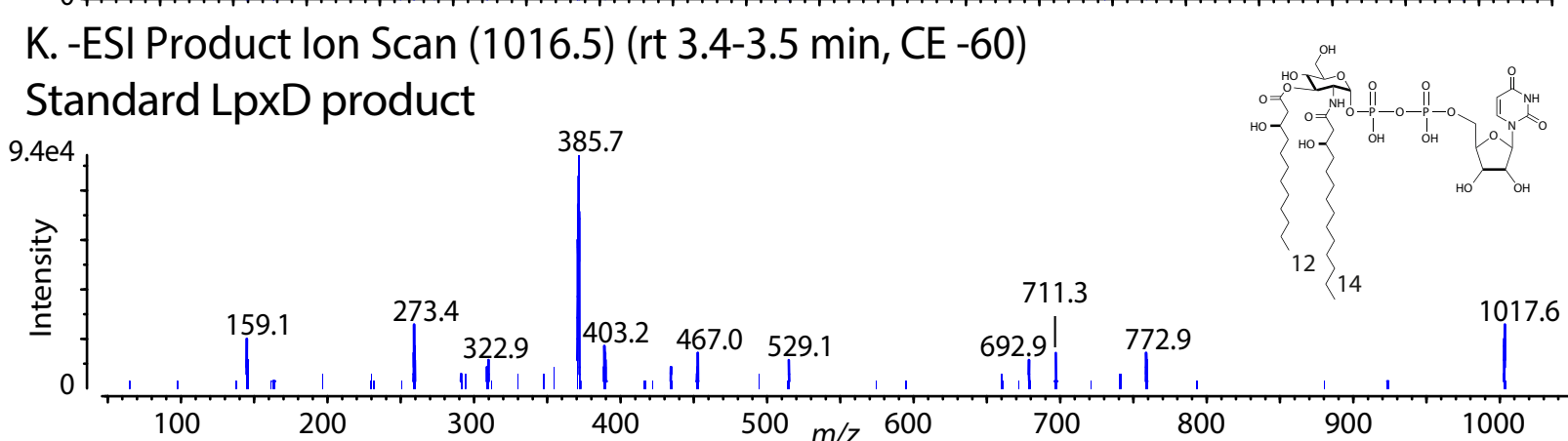

Supplement: S10 Fig — Chromatograms are provided for LpxD acyl chain variants from both experimental samples and authentic standards. The specific MRM transition being monitored as described in S2 Table is noted. Retention times are annotated. Peaks are labeled with (MS/MS) if product ion spectra were obtained for the specific peak. QQQ MS/MS spectra are displayed with peaks annotated. Product ion peaks are summarized in S4 Table and putative structural assignments are made in S18 Fig. In cases where a chromatographic peak is observed a proposed structure is provided. Acyl chain positions are for illustrative purposes only, based upon the final Lipid A structure. Our analysis does not clarify whether the species is C12 / C14, C14/ C12, or a mixture of these. (PDF) [file pone.0160918.s010.pdf]

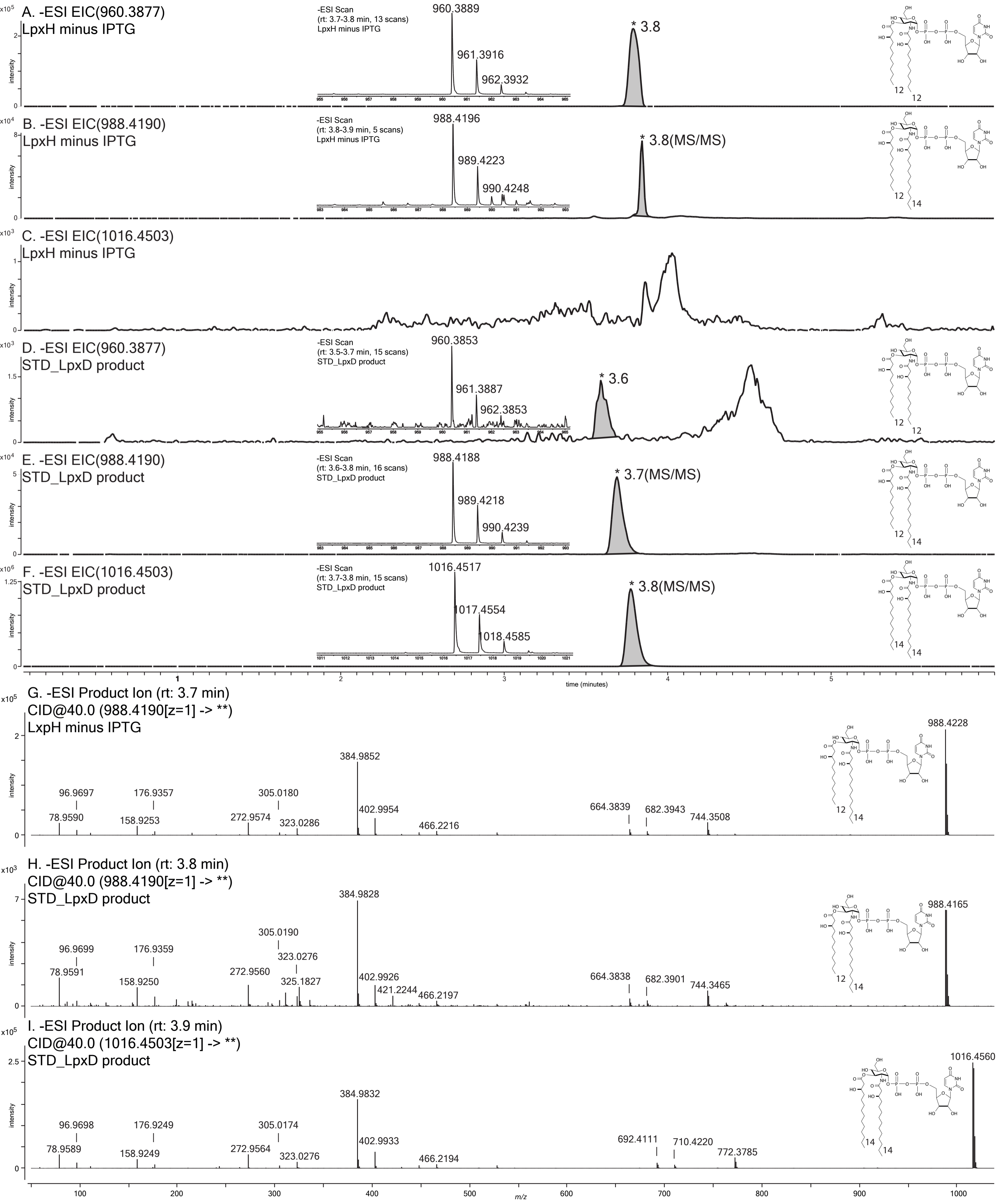

Supplement: S11 Fig — Chromatograms are provided for LpxD acyl chain variants from both experimental samples and authentic standards. The specific Extracted Ion Chromatogram (EIC) being monitored as described in S3 Table is noted. Retention times are annotated. Peaks are labeled with (MS/MS) if product ion spectra were obtained for the specific peak. QTOF MS/MS spectra are displayed with peaks annotated. Product ion peaks are summarized in S4 Table and putative structural assignments are made in S18 Fig. In cases where a chromatographic peak is observed a proposed structure is provided. Acyl chain positions are for illustrative purposes only, based upon the final Lipid A structure. Our analysis does not clarify whether the species is C12 / C14, C14/ C12, or a mixture of these. (PDF) [file pone.0160918.s011.pdf]

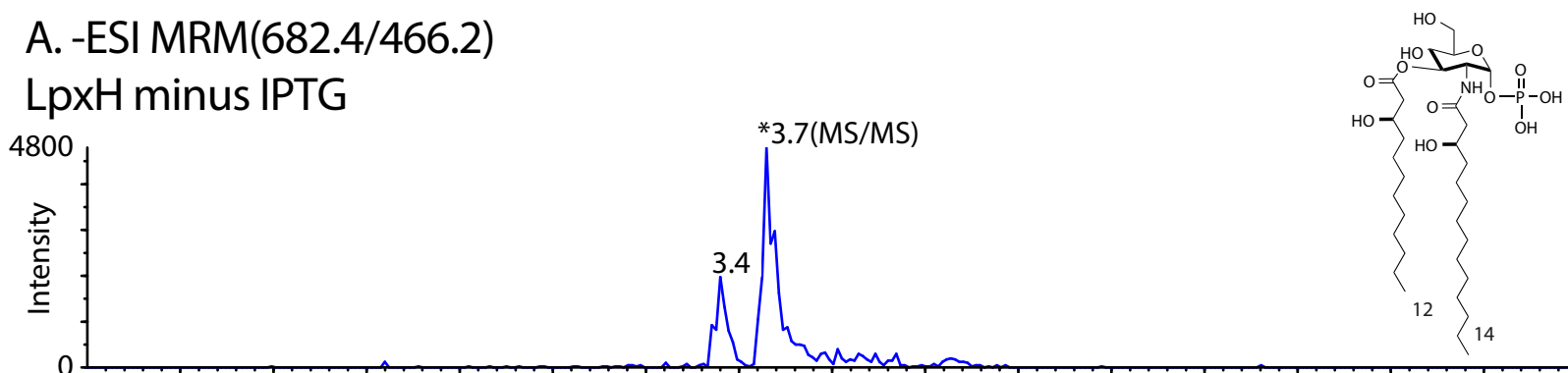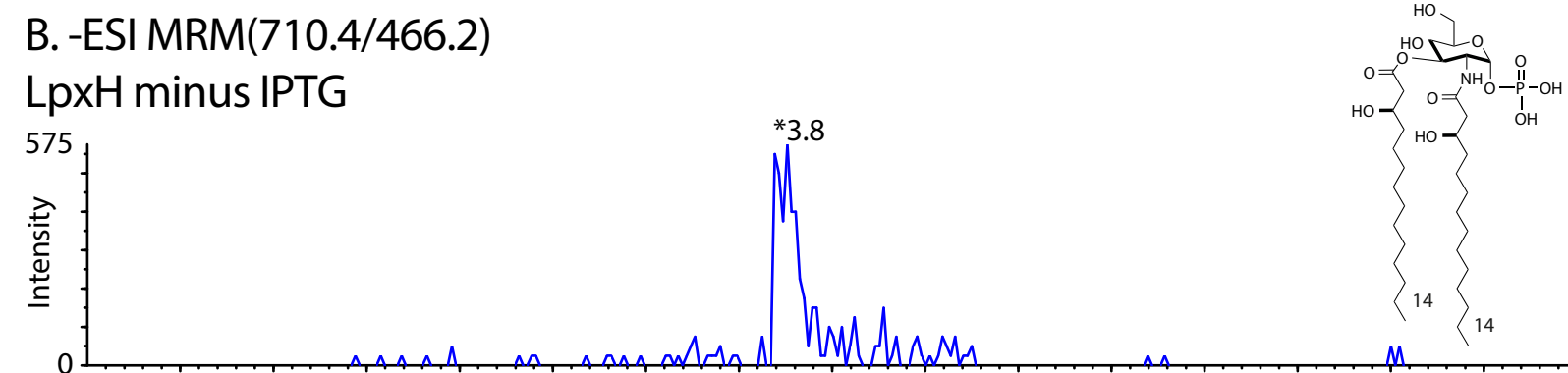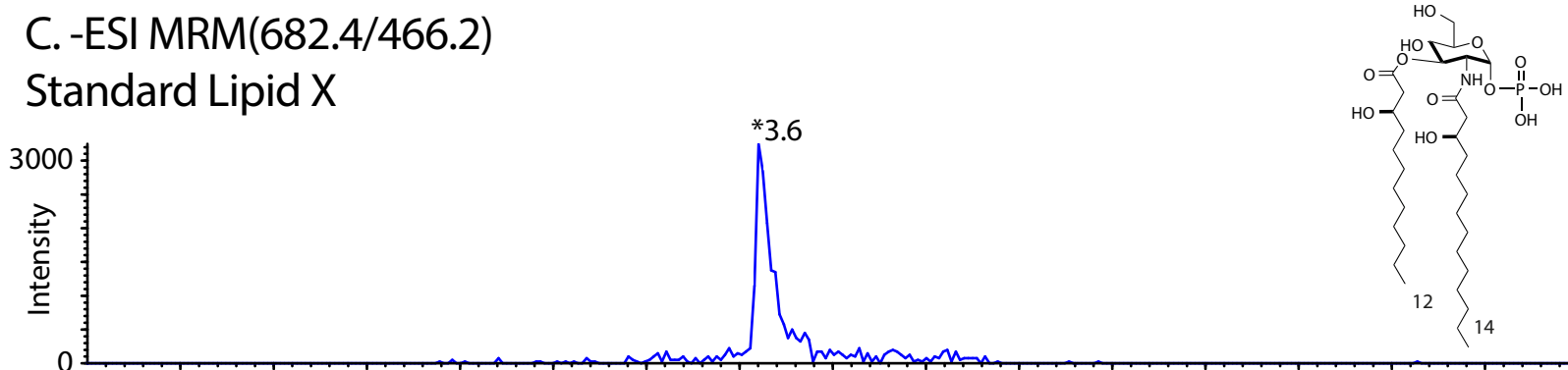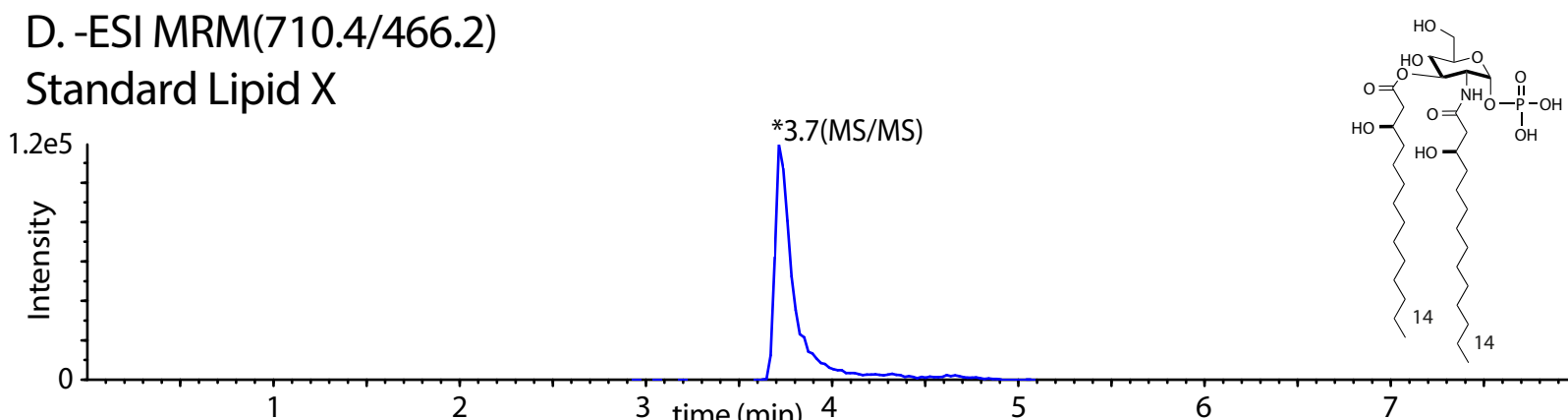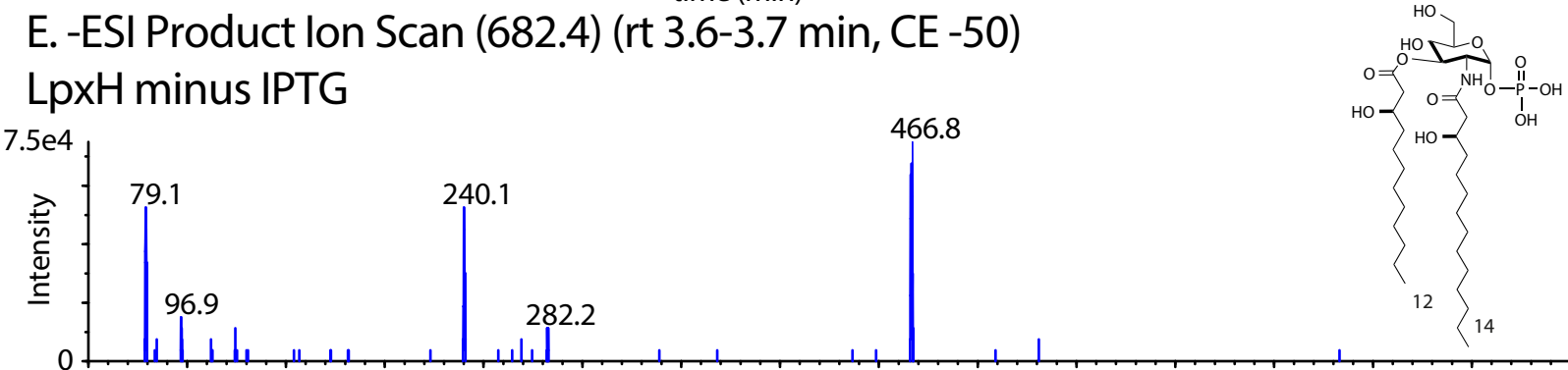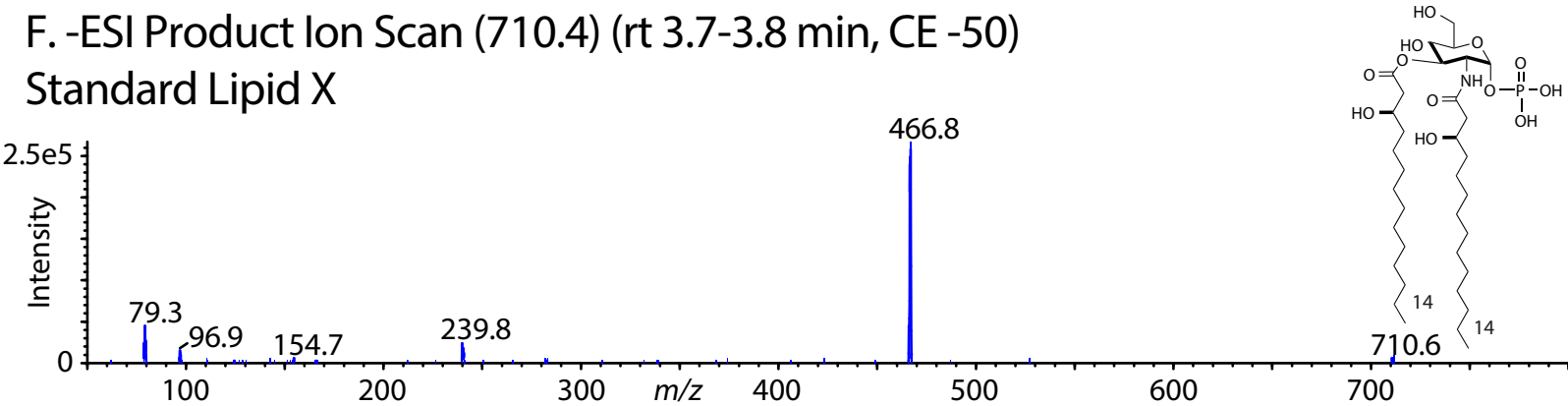

Supplement: S12 Fig — Chromatograms are provided for Lipid X acyl chain variants from both experimental samples and authentic standards. The specific MRM transition being monitored as described in S2 Table is noted. Retention times are annotated. Peaks are labeled with (MS/MS) if product ion spectra were obtained for the specific peak. QQQ MS/MS spectra are displayed with peaks annotated. Product ion peaks are summarized in S4 Table and putative structural assignments are made in S18 Fig. In cases where a chromatographic peak is observed a proposed structure is provided. Acyl chain positions are for illustrative purposes only, based upon the final Lipid A structure. Our analysis does not clarify whether the species is C12 / C14, C14/ C12, or a mixture of these. (PDF) [file pone.0160918.s012.pdf]

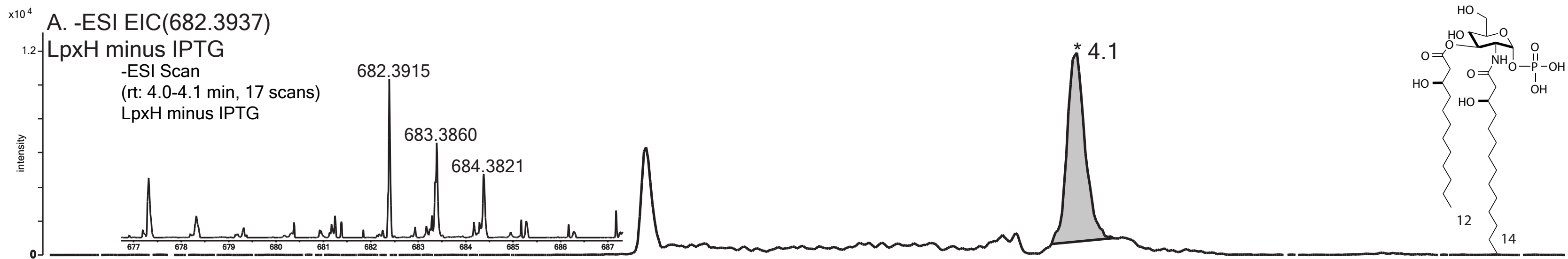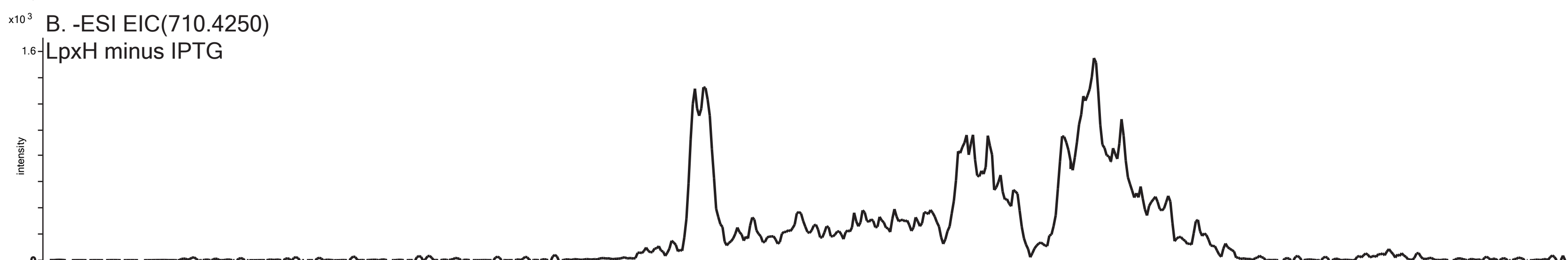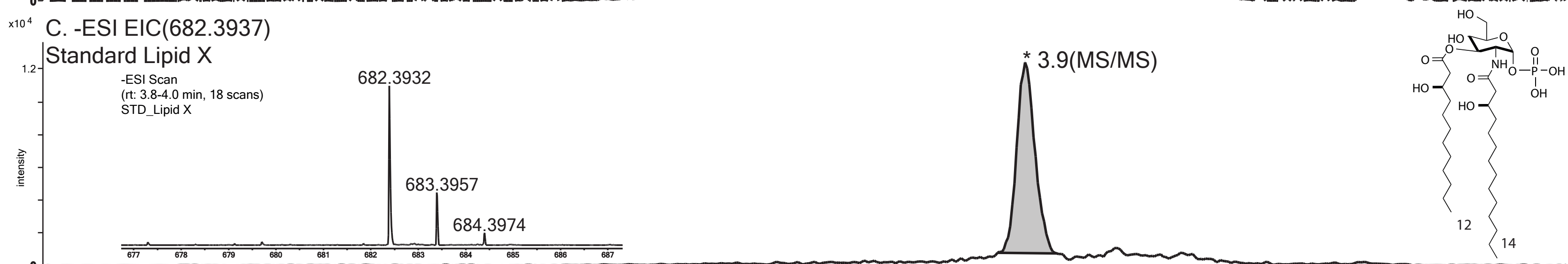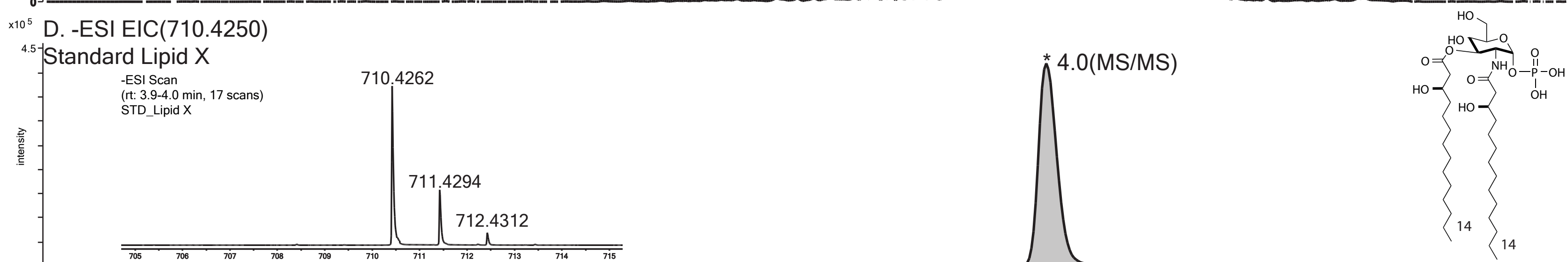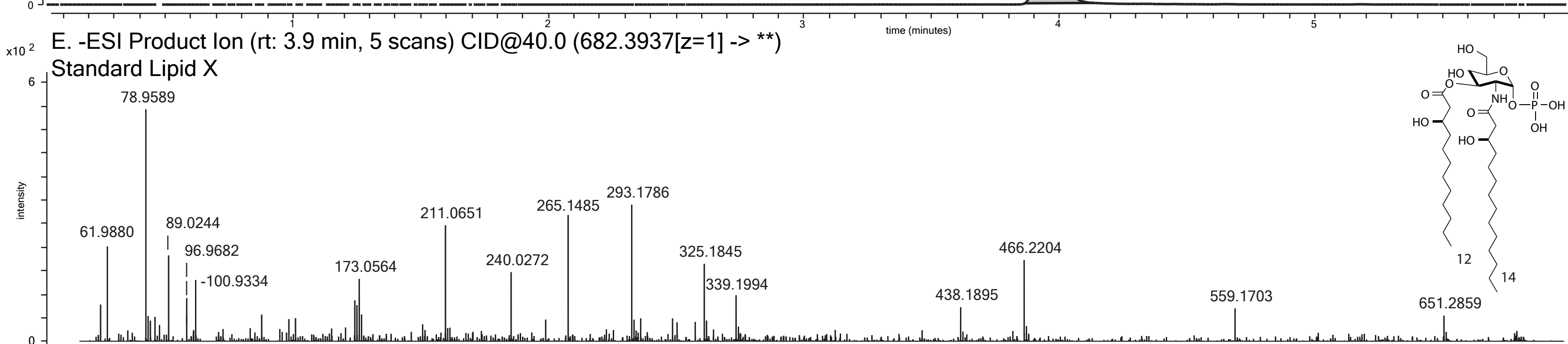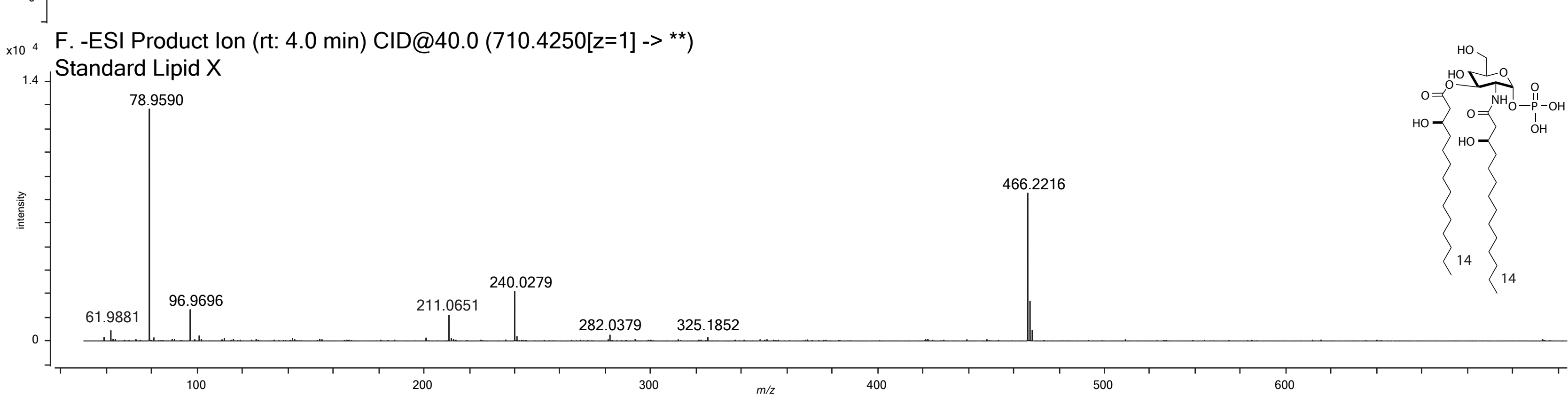

Supplement: S13 Fig — Chromatograms are provided for Lipid X acyl chain variants from both experimental samples and authentic standards. The specific Extracted Ion Chromatogram (EIC) being monitored as described in S3 Table is noted. Retention times are annotated. Peaks are labeled with (MS/MS) if product ion spectra were obtained for the specific peak. QTOF MS/MS spectra are displayed with peaks annotated. Product ion peaks are summarized in S4 Table and putative structural assignments are made in S18 Fig. In cases where a chromatographic peak is observed a proposed structure is provided. Acyl chain positions are for illustrative purposes only, based upon the final Lipid A structure. Our analysis does not clarify whether the species is C12 / C14, C14/ C12, or a mixture of these. (PDF) [file pone.0160918.s013.pdf]

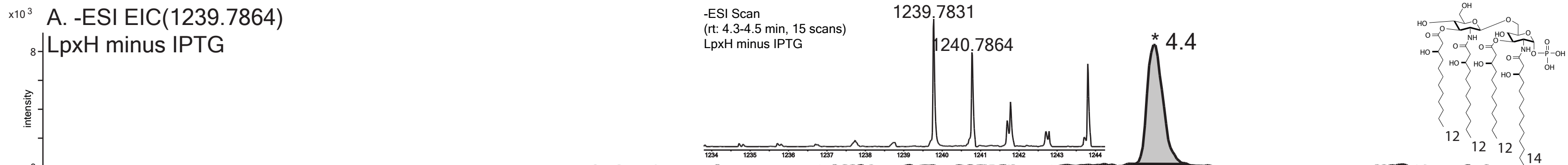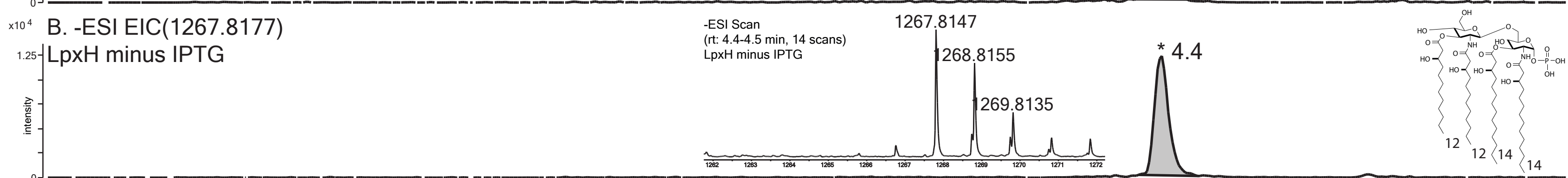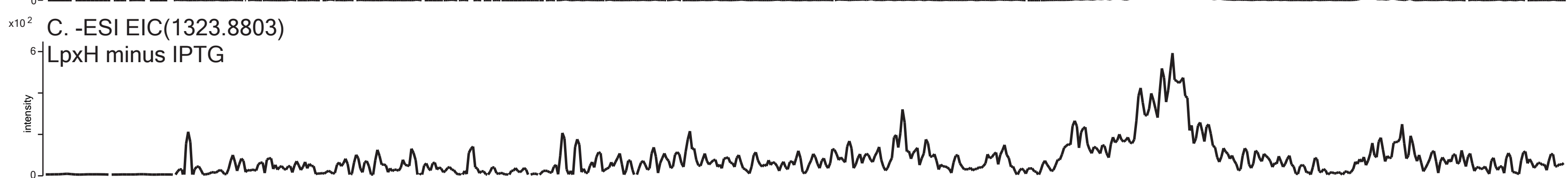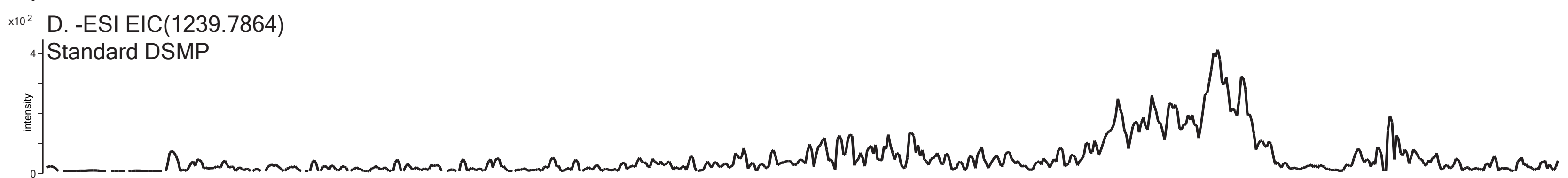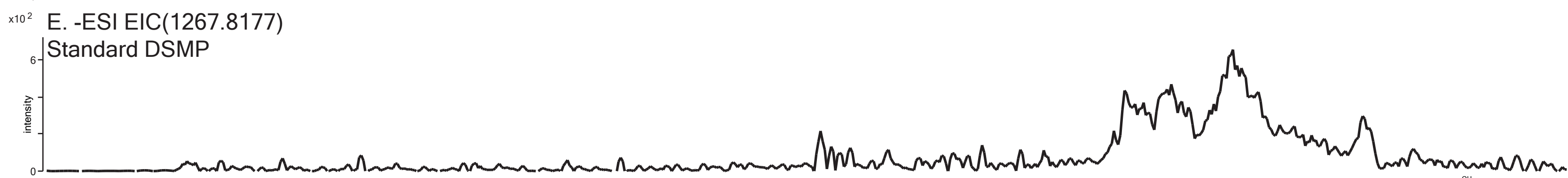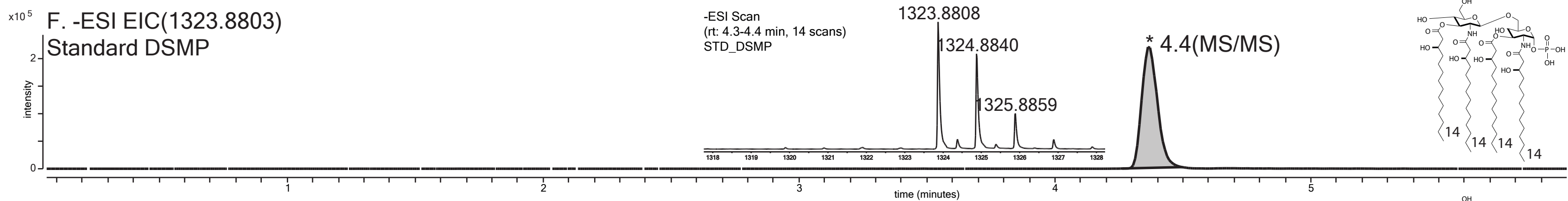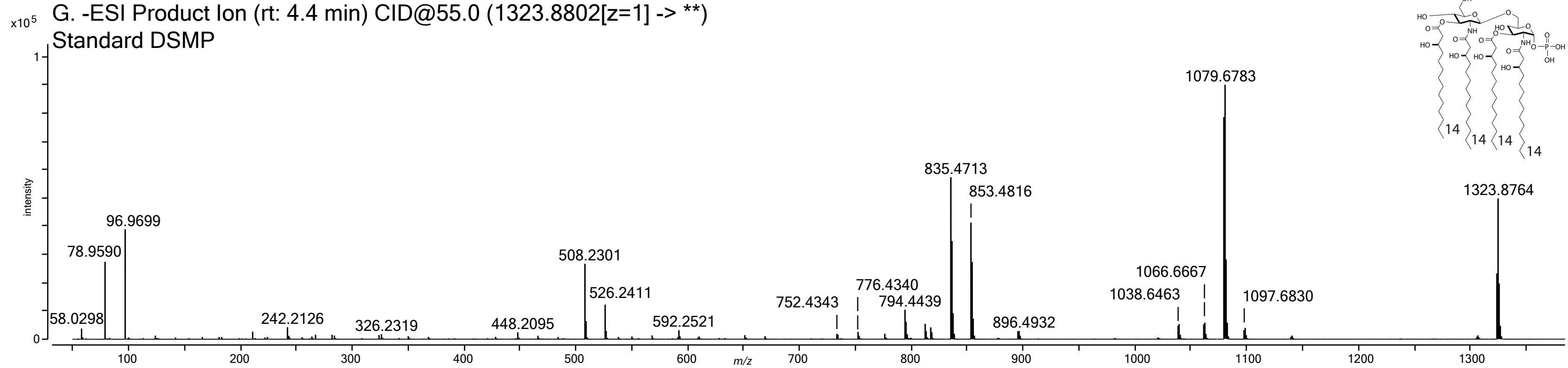

Supplement: S15 Fig — Chromatograms are provided for DSMP acyl chain variants from both experimental samples and authentic standards. The specific Extracted Ion Chromatogram (EIC) being monitored as described in S3 Table is noted. Retention times are annotated. Peaks are labeled with (MS/MS) if product ion spectra were obtained for the specific peak. QTOF MS/MS spectra are displayed with peaks annotated. Product ion peaks are summarized in S4 Table and putative structural assignments are made in S18 Fig. In cases where a chromatographic peak is observed a proposed structure is provided. Acyl chain positions are for illustrative purposes only, based upon the final Lipid A structure. Our analysis does not clarify whether the species is C12 / C14 / C12 / C14 or C12 / C12 / C14 / C14, or a mixture of these. (PDF) [file pone.0160918.s015.pdf]

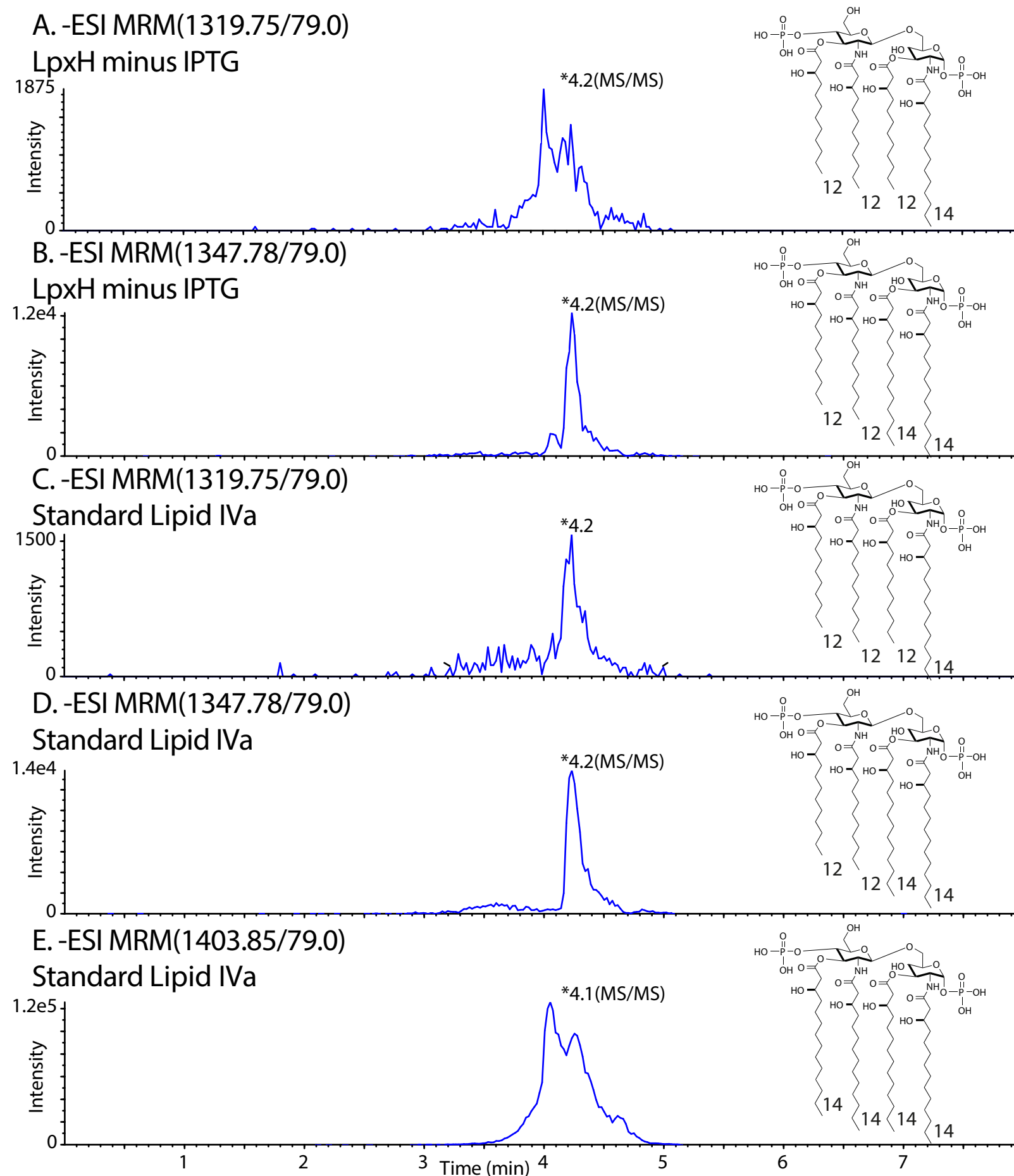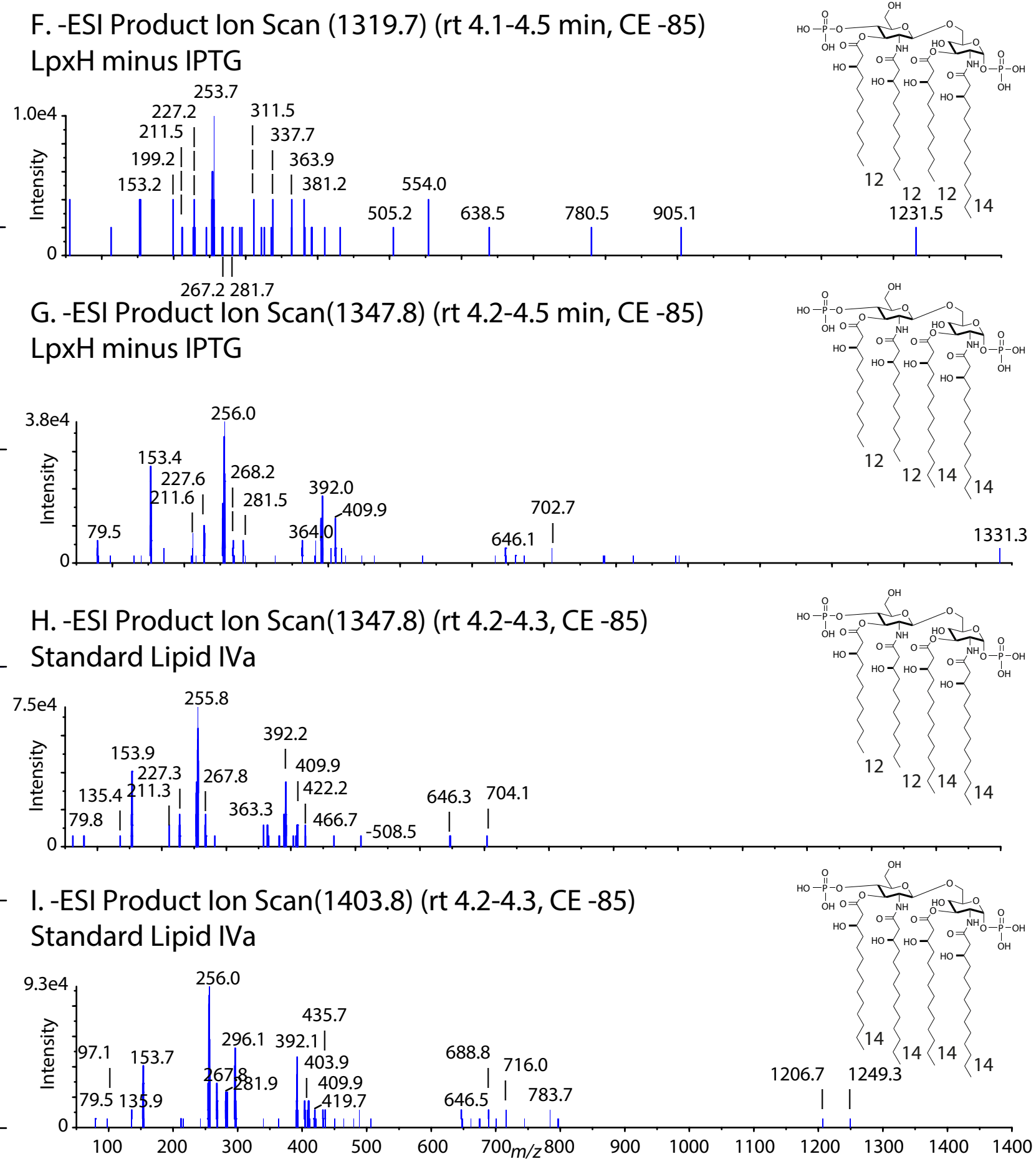

Supplement: S16 Fig — Chromatograms are provided for Lipid IVa acyl chain variants from both experimental samples and authentic standards. The specific MRM transition being monitored as described in S2 Table is noted. Retention times are annotated. Peaks are labeled with (MS/MS) if product ion spectra were obtained for the specific peak. QQQ MS/MS spectra are displayed with peaks annotated. Product ion peaks are summarized in S4 Table and putative structural assignments are made in S18 Fig. In cases where a chromatographic peak is observed a proposed structure is provided. Acyl chain positions are for illustrative purposes only, based upon the final Lipid A structure. Our analysis does not clarify whether the species is C12 / C14 / C12 / C14 or C12 / C12 / C14 / C14, or a mixture of these. (PDF) [file pone.0160918.s016.pdf]

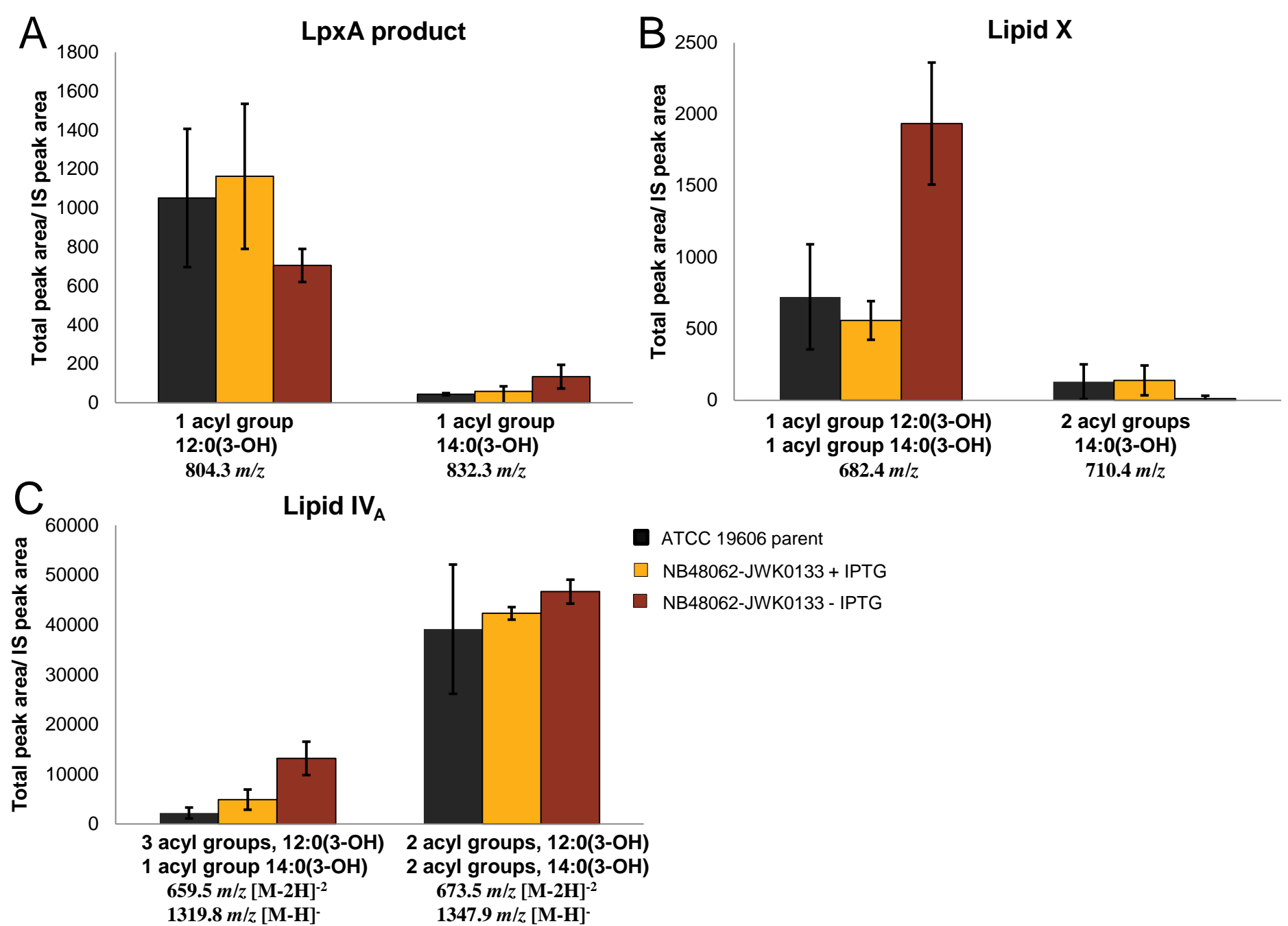

Supplement: S19 Fig — (A) LCMS-MRM quantification of LpxA product with 1 acyl group 12:0(3-OH) and 1 acyl group 14:0(3-OH) is shown for A. baumannii ATCC 19606 parent and NB48062-JWK0133 under inducing and non-inducing conditions. (B) LCMS-MRM quantification of lipid X with 1 acyl group 12:0(3-OH) and 1 acyl group 14:0(3-OH) compared with 2 acyl groups 14:0(3-OH) is shown for A. baumannii ATCC 19606 parent and NB48062-JWK0133 under inducing and non-inducing conditions. (C) Lipid IVA with three acyl groups with 12:0(3-OH) and one acyl group of 14:0(3-OH), compared with two acyl groups 12:0(3-OH) and two acyl groups 14:0(3-OH) for A. baumannii ATCC 19606 parent and NB48062-JWK0133 under inducing and non-inducing conditions. Experiments were performed in triplicate and bars show the mean value and SD) between NB46082-JWK0133 in the presence or absence of IPTG. Data shown was normalized to an internal standard (IS) as previously described [40]. (PDF) [file pone.0160918.s019.pdf]

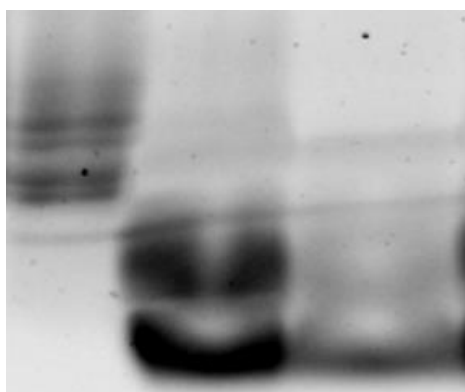

**1**      **2**      **3**

Supplement: S20 Fig — Lane 1, E. coli LPS standard; Lane 2, A. baumannii ATCC 19606 parent; Lane 3, A. baumannii ATCC 19606 + 8 μg/ml CHIR-090. Experiments were performed as previously described [40]. (PDF) [file pone.0160918.s020.pdf]
