# Supplementary material for: Toxic Accumulation of LPS Pathway Intermediates Underlies the Requirement of LpxH for Growth of Acinetobacter baumannii ATCC 19606
Source: PLoS One. 2016 Aug 15;11(8):e0160918. doi: 10.1371/journal.pone.0160918 (PMC4985137; doi:10.1371/journal.pone.0160918)
Supplement: S3 Fig — The alaS gene was replaced with a gentamicin resistance cassette (accC1) using double homologous recombination. (PDF) [file pone.0160918.s003.pdf]

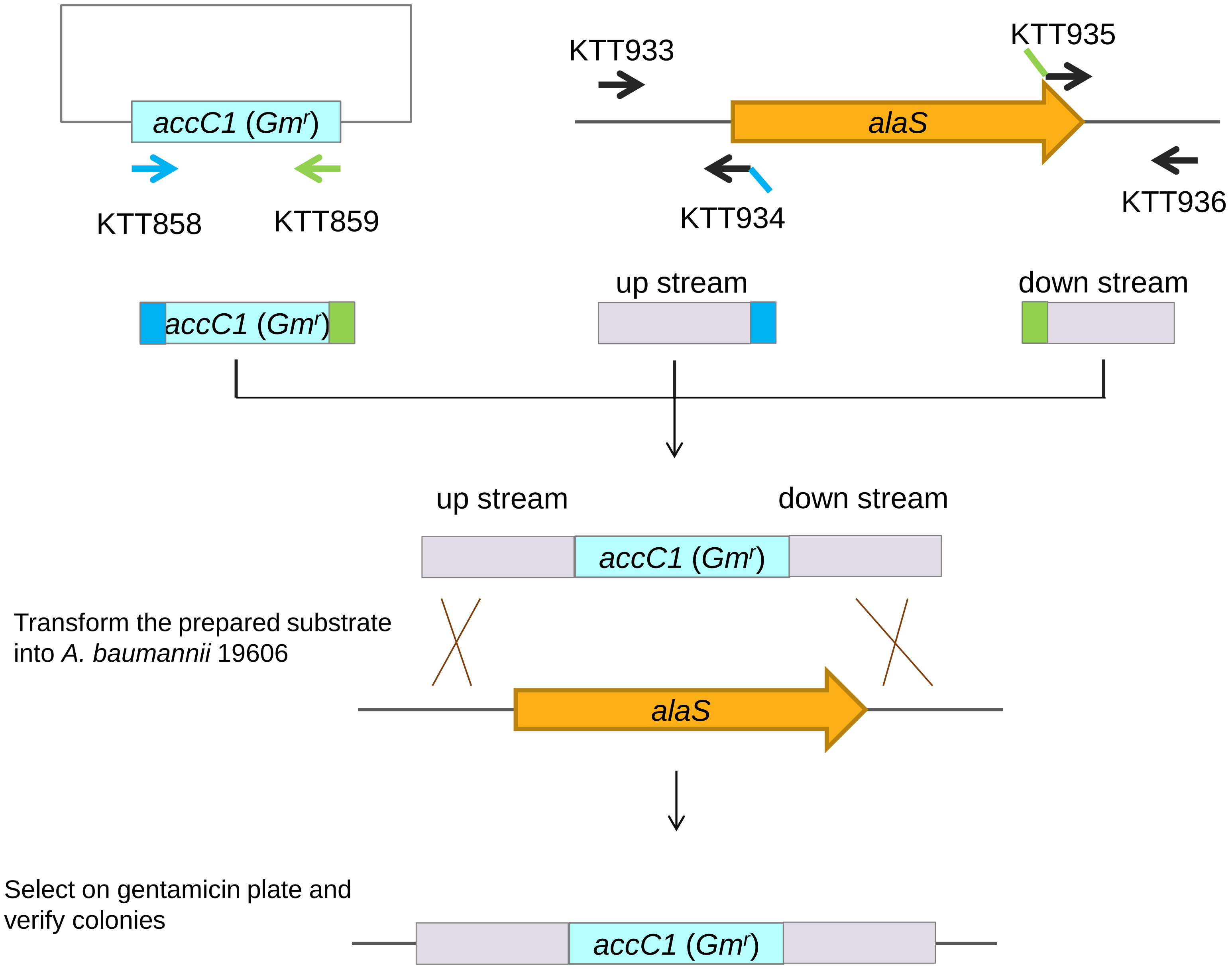

accC1 (Gm^r)
KTT858
KTT859
KTT933
KTT935
alaS
KTT934
KTT936
accC1 (Gm^r)
up stream
down stream
up stream
down stream
accC1 (Gm^r)
Transform the prepared substrate into *A. baumannii* 19606
alaS
Select on gentamicin plate and verify colonies
accC1 (Gm^r)
