# Supplementary material for: Toxic Accumulation of LPS Pathway Intermediates Underlies the Requirement of LpxH for Growth of Acinetobacter baumannii ATCC 19606
Source: PLoS One. 2016 Aug 15;11(8):e0160918. doi: 10.1371/journal.pone.0160918 (PMC4985137; doi:10.1371/journal.pone.0160918)
Supplement: S6 Fig — Chromatograms are provided for LpxA acyl chain variants from both experimental samples and authentic standards. The specific MRM transition being monitored as described in S2 Table is noted. Retention times are annotated. Peaks are labeled with (MS/MS) if product ion spectra were obtained for the specific peak. QQQ MS/MS spectra are displayed with peaks annotated. Product ion peaks are summarized in S4 Table and putative structural assignments are made in S18 Fig. In cases where a chromatographic peak is observed, a proposed structure is provided. (PDF) [file pone.0160918.s006.pdf]

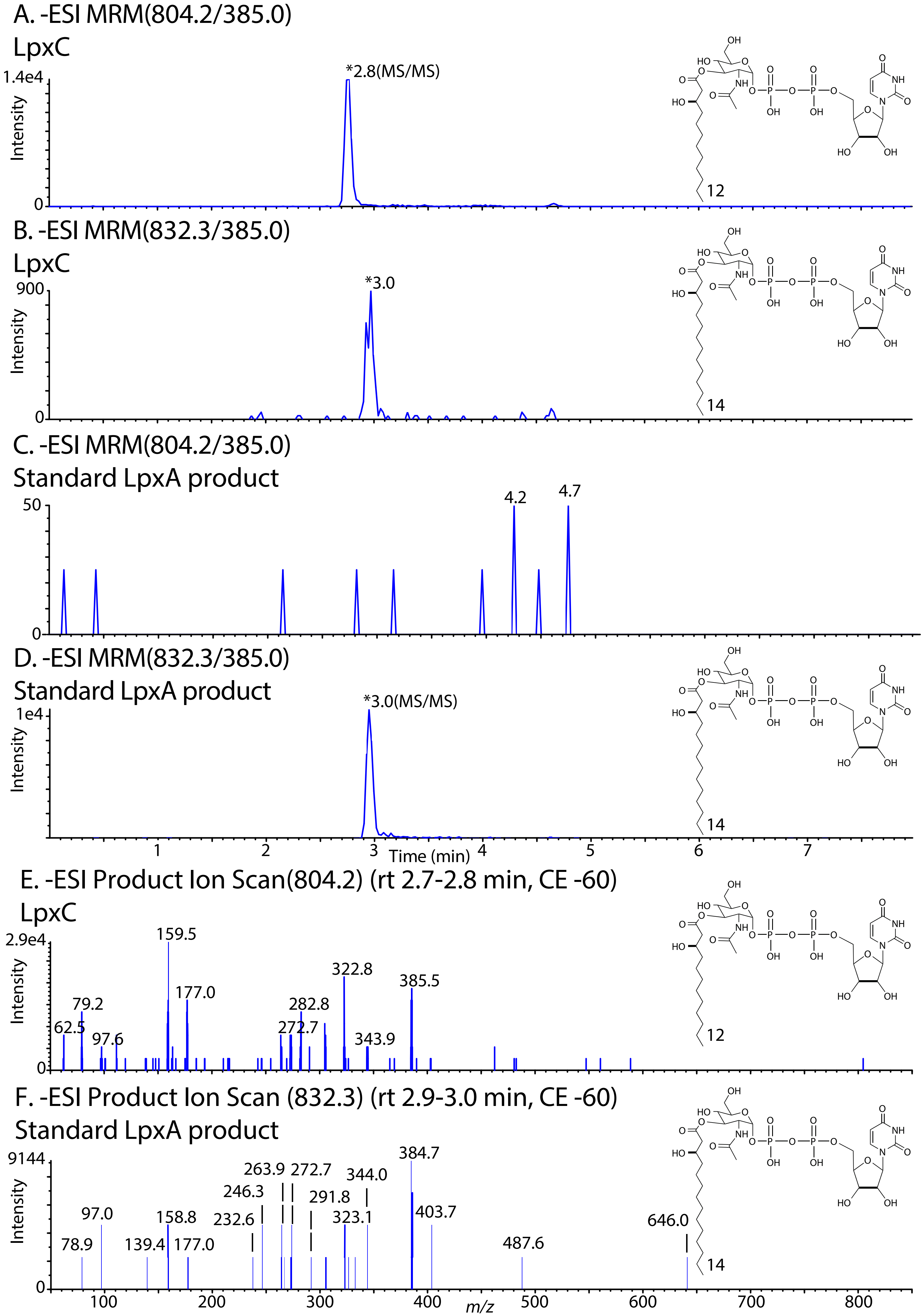

A. -ESI MRM(804.2/385.0)
LpxC
1.4e4
0
Intensity
*2.8(MS/MS)
12
B. -ESI MRM(832.3/385.0)
LpxC
900
0
Intensity
*3.0
14
C. -ESI MRM(804.2/385.0)
Standard LpxA product
50
0
4.2
4.7
D. -ESI MRM(832.3/385.0)
Standard LpxA product
1e4
Intensity
*3.0(MS/MS)
14
1
2
3
4
5
6
7
Time (min)
E. -ESI Product Ion Scan(804.2) (rt 2.7-2.8 min, CE -60)
LpxC
2.9e4
0
Intensity
159.5
322.8
385.5
177.0
79.2
282.8
62.5
97.6
272.7
343.9
12
F. -ESI Product Ion Scan (832.3) (rt 2.9-3.0 min, CE -60)
Standard LpxA product
9144
0
Intensity
384.7
263.9
272.7
344.0
246.3
291.8
97.0
158.8
232.6
323.1
403.7
646.0
78.9
139.4
177.0
487.6
14
100
200
300
400
500
600
700
800
m/z
