# Supplementary material for: Toxic Accumulation of LPS Pathway Intermediates Underlies the Requirement of LpxH for Growth of Acinetobacter baumannii ATCC 19606
Source: PLoS One. 2016 Aug 15;11(8):e0160918. doi: 10.1371/journal.pone.0160918 (PMC4985137; doi:10.1371/journal.pone.0160918)
Supplement: S8 Fig — Chromatograms are provided for LpxC acyl chain variants from both experimental samples and authentic standards. The specific MRM transition being monitored as described in S2 Table is noted. Retention times are annotated. Peaks are labeled with (MS/MS) if product ion spectra were obtained for the specific peak. QQQ MS/MS spectra are displayed with peaks annotated. Product ion peaks are summarized in S4 Table and putative structural assignments are made in S18 Fig. In cases where a chromatographic peak is observed, a proposed structure is provided. (PDF) [file pone.0160918.s008.pdf]

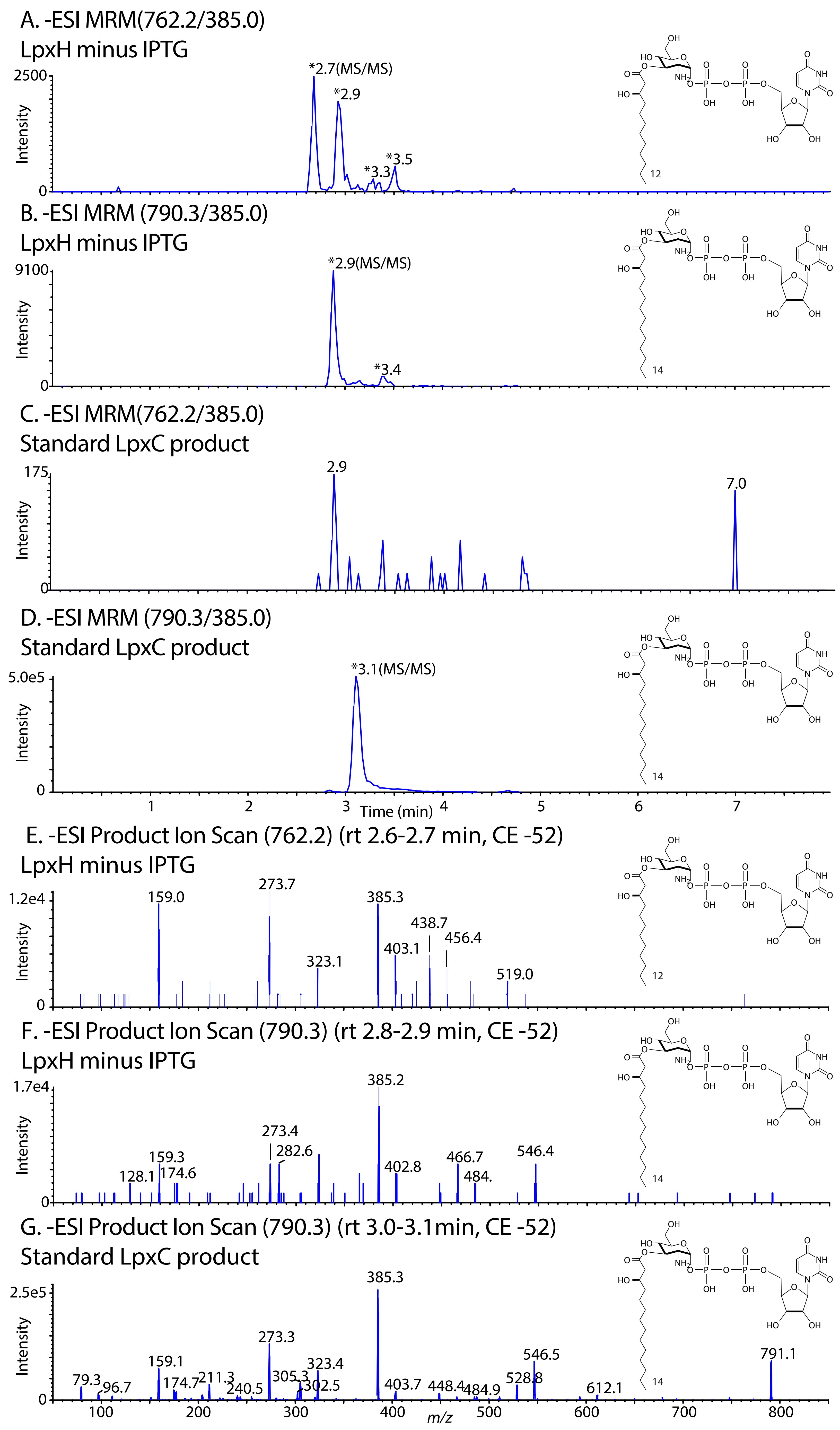
A. -ESI MRM(762.2/385.0)
LpxH minus IPTG
2500
0
Intensity
*2.7(MS/MS)
*2.9
*3.3
*3.5
12
B. -ESI MRM (790.3/385.0)
LpxH minus IPTG
9100
0
Intensity
*2.9(MS/MS)
*3.4
14
C. -ESI MRM(762.2/385.0)
Standard LpxC product
175
0
Intensity
2.9
7.0
D. -ESI MRM (790.3/385.0)
Standard LpxC product
5.0e5
0
Intensity
*3.1(MS/MS)
14
1 2 3 4 5 6 7
Time (min)
E. -ESI Product Ion Scan (762.2) (rt 2.6-2.7 min, CE -52)
LpxH minus IPTG
1.2e4
0
Intensity
159.0
273.7
385.3
323.1
403.1
438.7
456.4
519.0
12
F. -ESI Product Ion Scan (790.3) (rt 2.8-2.9 min, CE -52)
LpxH minus IPTG
1.7e4
0
Intensity
385.2
273.4
282.6
159.3
128.1
174.6
402.8
466.7
484.
546.4
14
G. -ESI Product Ion Scan (790.3) (rt 3.0-3.1min, CE -52)
Standard LpxC product
2.5e5
0
Intensity
385.3
273.3
159.1
79.3
96.7
174.7
211.3
240.5
305.3
302.5
323.4
403.7
448.4
484.9
528.8
546.5
612.1
791.1
14
100 200 300 400 500 600 700 800
m/z
HO
OH
O
NH2
P
OH
NH
N
O
HO
