# Supplementary material for: Toxic Accumulation of LPS Pathway Intermediates Underlies the Requirement of LpxH for Growth of Acinetobacter baumannii ATCC 19606
Source: PLoS One. 2016 Aug 15;11(8):e0160918. doi: 10.1371/journal.pone.0160918 (PMC4985137; doi:10.1371/journal.pone.0160918)
Supplement: S14 Fig — Chromatograms are provided for DSMP acyl chain variants from both experimental samples and authentic standards. The specific MRM transition being monitored as described in S2 Table is noted. Retention times are annotated. Peaks are labeled with (MS/MS) if product ion spectra were obtained for the specific peak. QQQ MS/MS spectra are displayed with peaks annotated. Product ion peaks are summarized in S4 Table and putative structural assignments are made in S18 Fig. In cases where a chromatographic peak is observed a proposed structure is provided. Acyl chain positions are for illustrative purposes only, based upon the final Lipid A structure. Our analysis does not clarify whether the species is C12 / C14 / C12 / C14 or C12 / C12 / C14 / C14, or a mixture of these. (PDF) [file pone.0160918.s014.pdf]

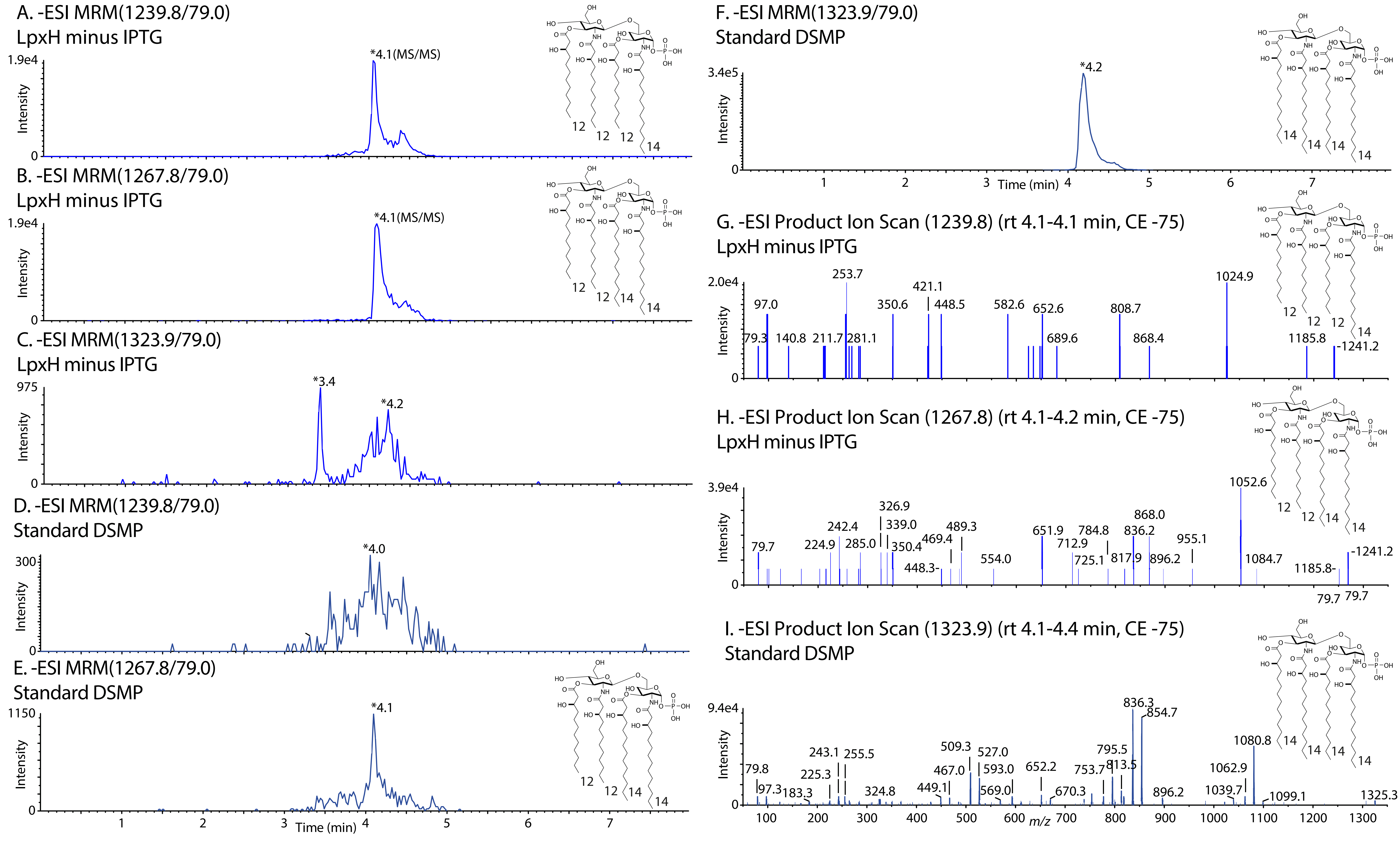

A. -ESI MRM(1239.8/79.0)
LpxH minus IPTG
*4.1(MS/MS)
B. -ESI MRM(1267.8/79.0)
LpxH minus IPTG
*4.1(MS/MS)
C. -ESI MRM(1323.9/79.0)
LpxH minus IPTG
*3.4
*4.2
D. -ESI MRM(1239.8/79.0)
Standard DSMP
*4.0
E. -ESI MRM(1267.8/79.0)
Standard DSMP
*4.1
Time (min)
F. -ESI MRM(1323.9/79.0)
Standard DSMP
*4.2
Time (min)
G. -ESI Product Ion Scan (1239.8) (rt 4.1-4.1 min, CE -75)
LpxH minus IPTG
H. -ESI Product Ion Scan (1267.8) (rt 4.1-4.2 min, CE -75)
LpxH minus IPTG
I. -ESI Product Ion Scan (1323.9) (rt 4.1-4.4 min, CE -75)
Standard DSMP
Intensity
m/z
