# Supplementary material for: Toxic Accumulation of LPS Pathway Intermediates Underlies the Requirement of LpxH for Growth of Acinetobacter baumannii ATCC 19606
Source: PLoS One. 2016 Aug 15;11(8):e0160918. doi: 10.1371/journal.pone.0160918 (PMC4985137; doi:10.1371/journal.pone.0160918)

A: Proposed MS/MS Fragmentation pathways for LpxA product

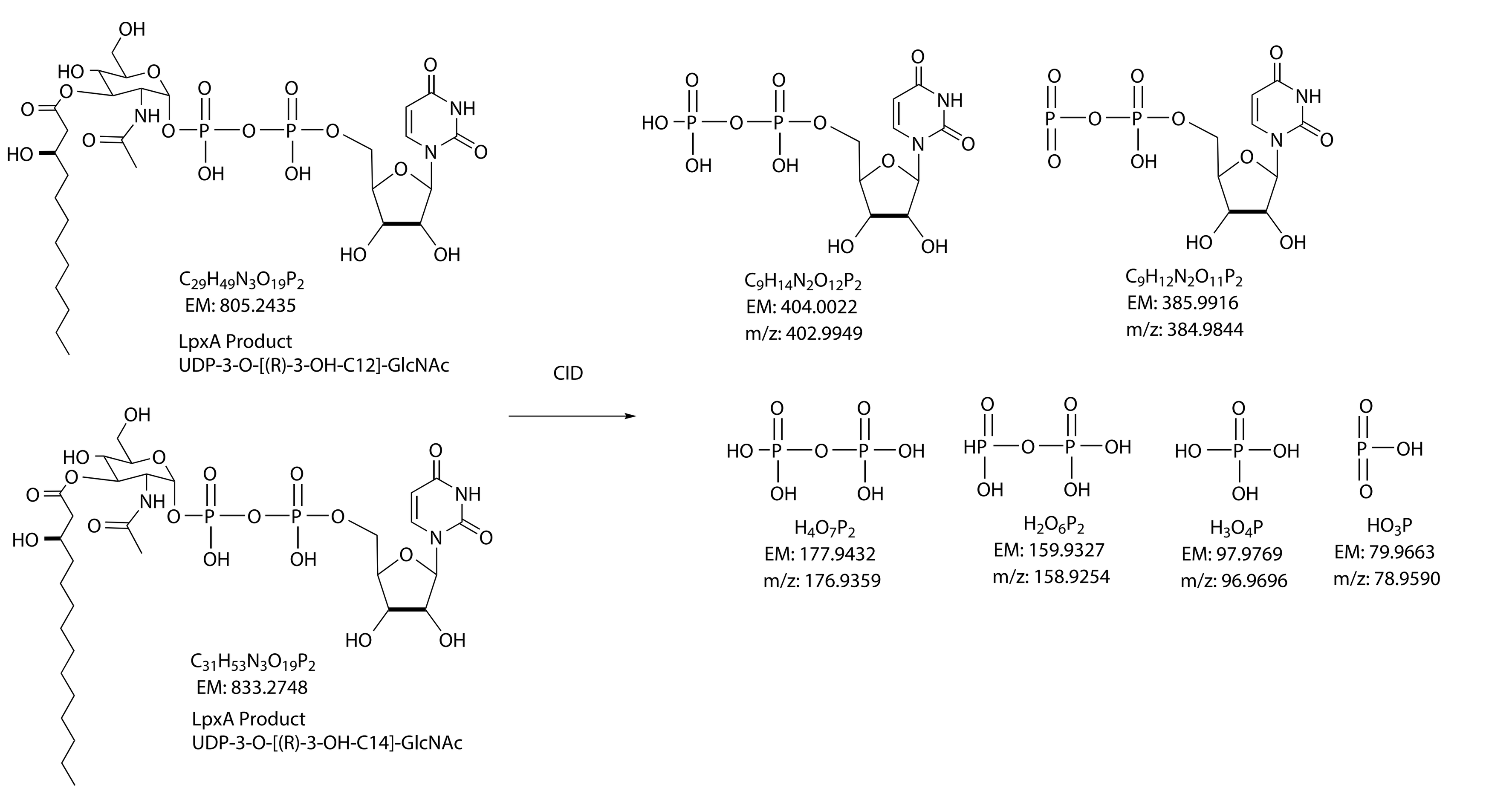

C: Proposed MS/MS fragmentation pathways for LpxD product

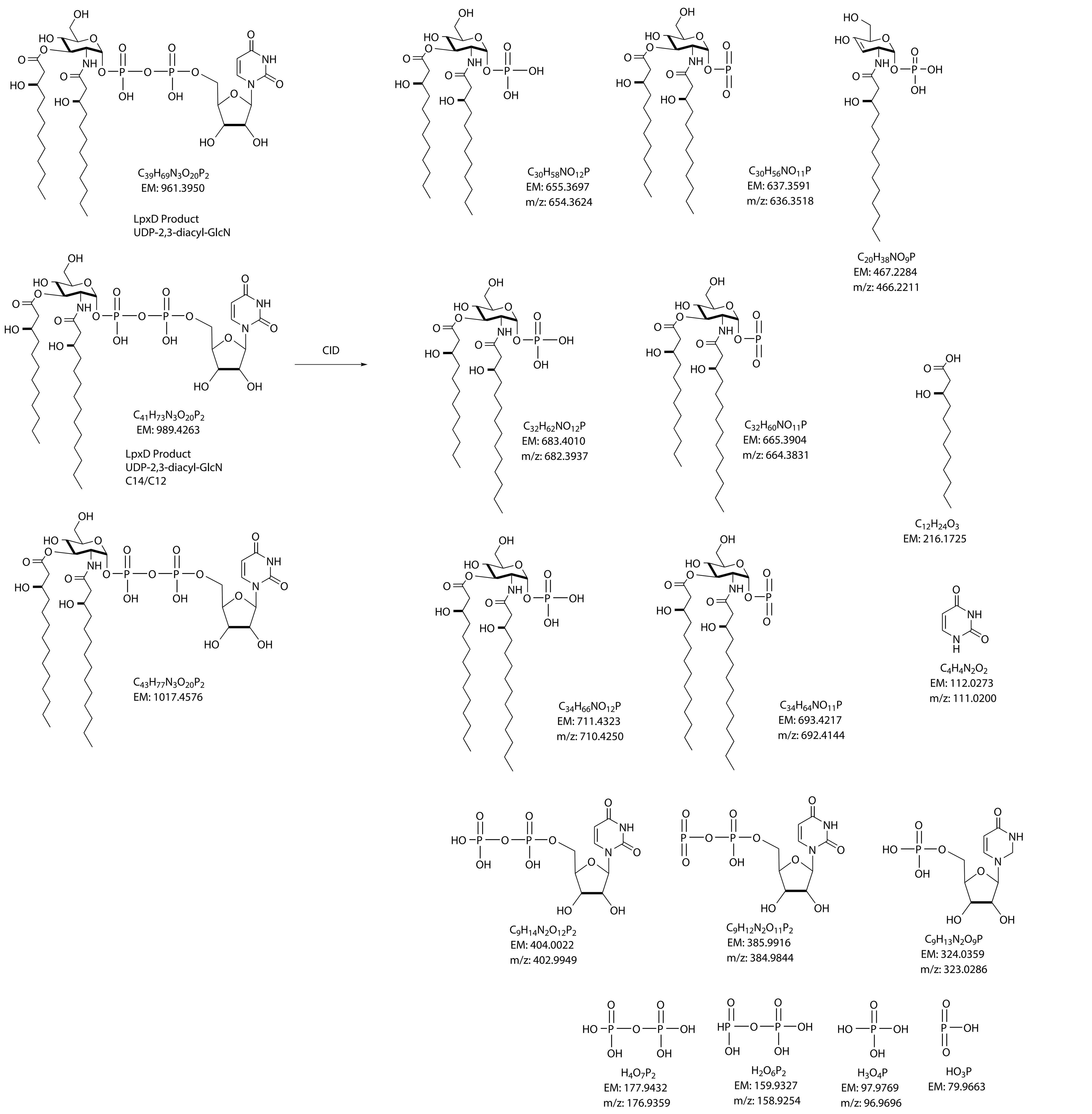

B: Proposed MS/MS fragmentation pathways for LpxC product

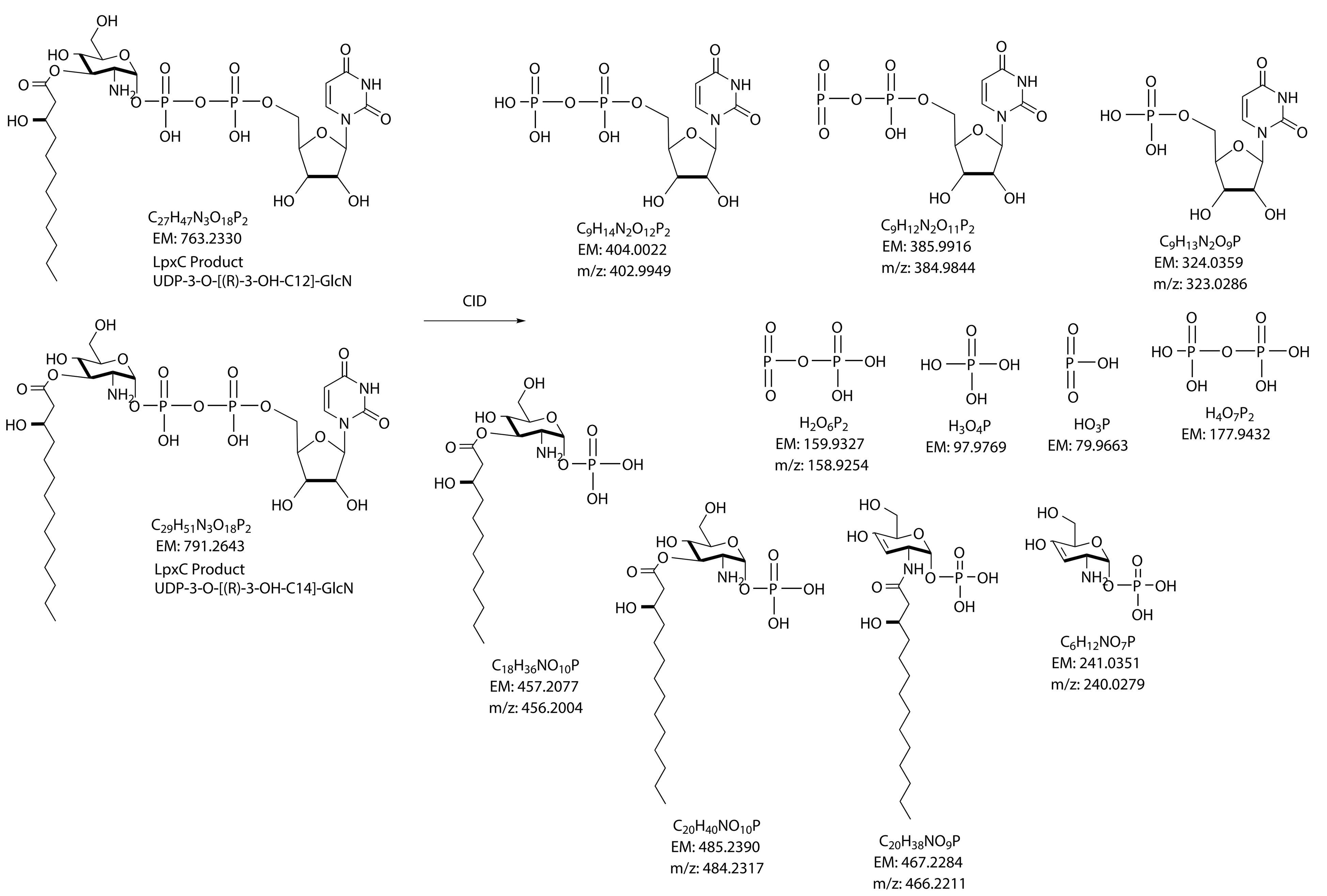

D: Proposed MS/MS fragmentation pathways for Lipid X

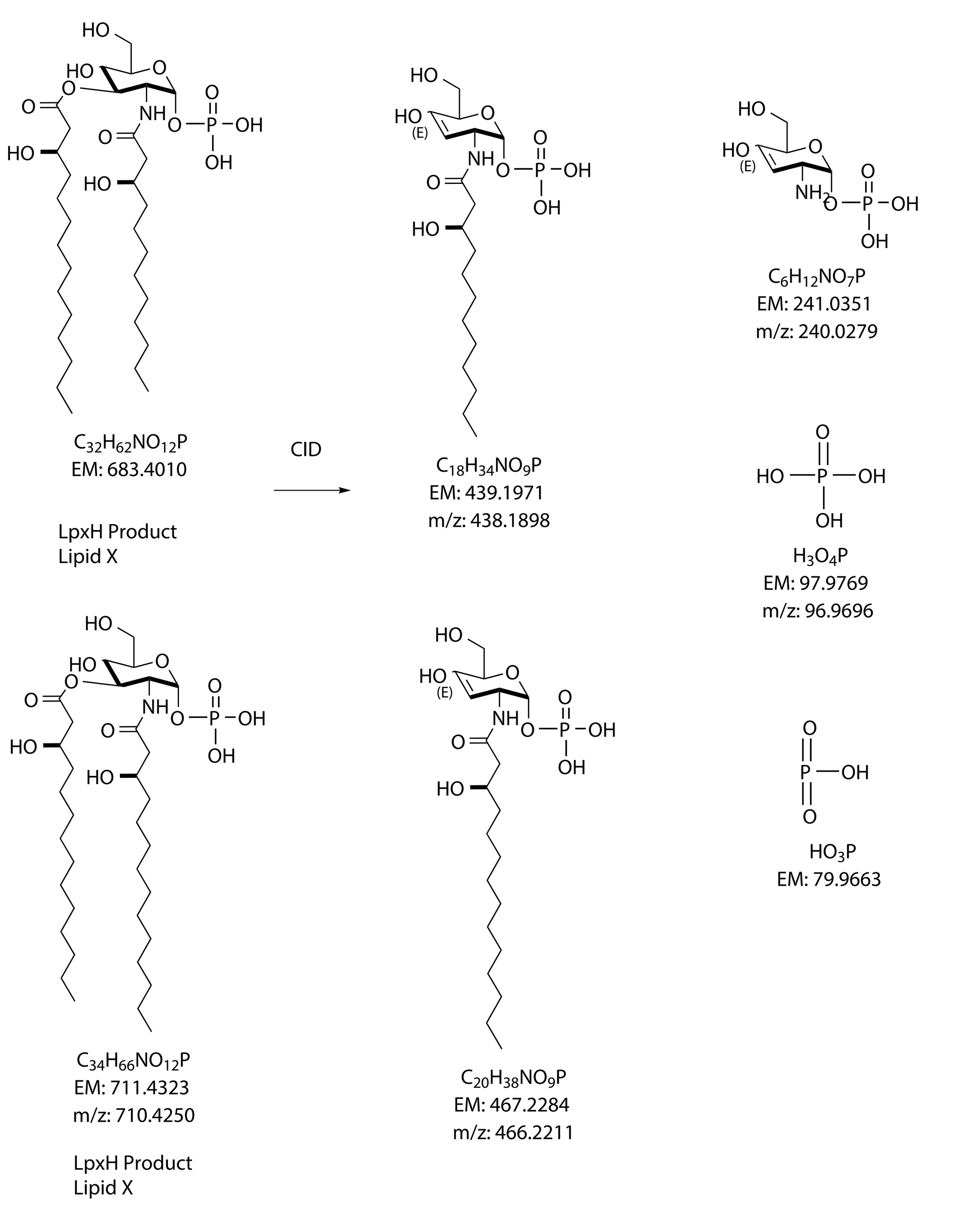

E: Proposed MS/MS fragmentation pathways for DSMP

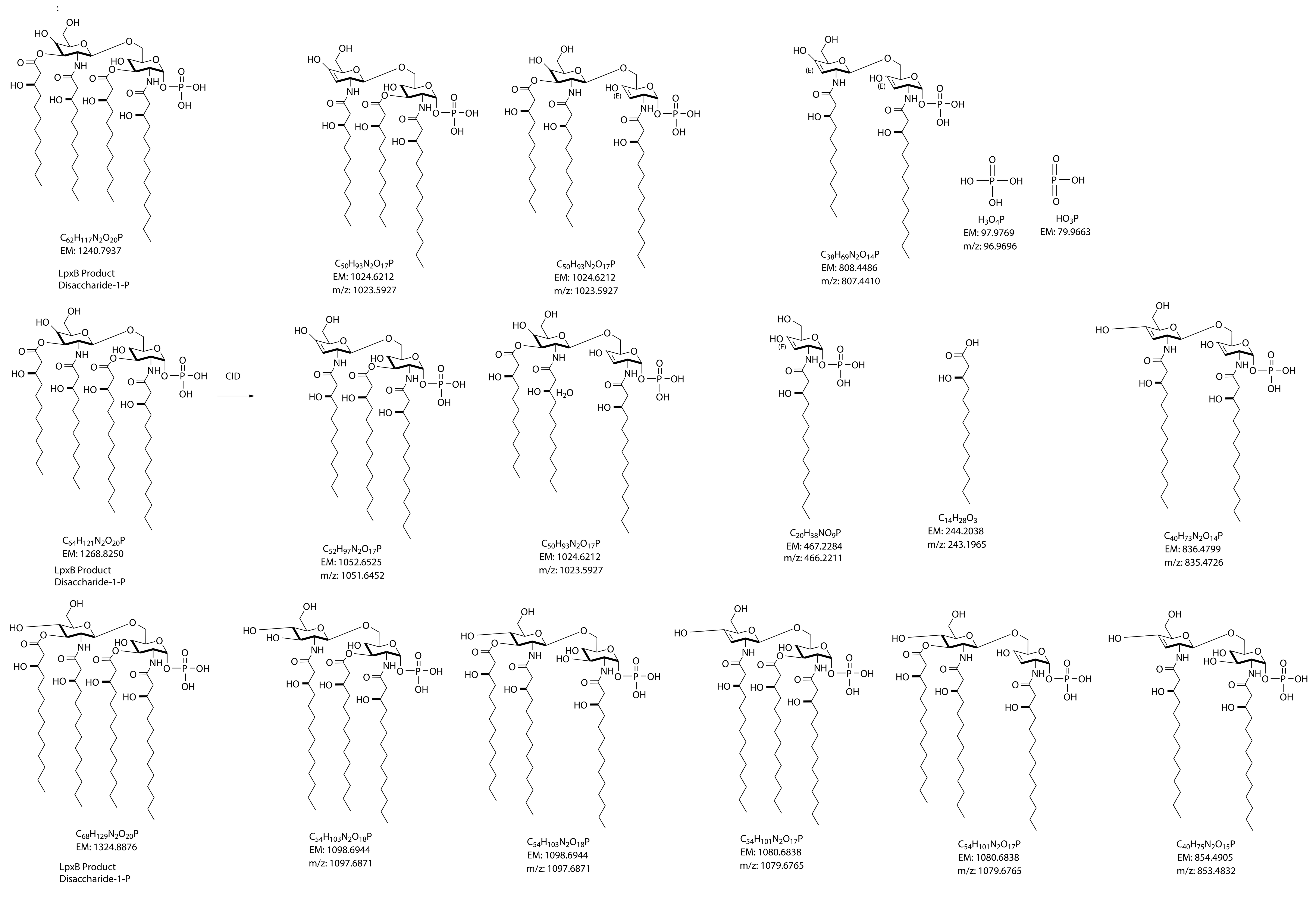

Supplement: S18 Fig — Proposed species are provided, consistent with reported MS/MS fragmentation for LPS intermediates and observed product ions. Species are drawn as neutral molecules with exact mass and m/z in the 1- charge state displayed. Acyl chain positions are for illustrative purposes only, based upon the final Lipid A structure. For example, it is not known if the species is C12 / C14 / C12 / C14 or C12 / C12 / C14 / C14, or a mixture from our analysis. See S4 Table for ions of interest. (PDF) [file pone.0160918.s018.pdf]
