# Supplementary material for: Toxic Accumulation of LPS Pathway Intermediates Underlies the Requirement of LpxH for Growth of Acinetobacter baumannii ATCC 19606
Source: PLoS One. 2016 Aug 15;11(8):e0160918. doi: 10.1371/journal.pone.0160918 (PMC4985137; doi:10.1371/journal.pone.0160918)
Supplement: S1 File — (DOCX) [file pone.0160918.s021.docx]

**Identification of LPS intermediates**

**Introduction**

The Raetz Pathway intermediates LpxA product (UDP-3-O-acyl-GlcNAc) through Lipid IV_A_ have been thoroughly described in the literature, however much of this information, such as MS/MS spectra, have been quite dispersed (1). For our studies, we have included the following for each pathway intermediate discussed in the manuscript: references spectra from the literature (2-8), QQQ LC-MRM chromatograms (Figures S6A-D, S8A-D, S10A-F, S12A-D, S14A-F, S16A-E), QQQ LC-MS/MS spectra (Figures S6E-F, S8E-G, S10G-K, S12E-F, S14G-I, S16F-I), QTOF LC-MS chromatograms (Figures S7 A-D, S9A-D, S11A-F, S13A-D, S15A-F, S17A-F), and QTOF LC-MS/MS spectra (Figures S7E, S9E, S11 G-I, S13E-F, S15G, S17G). These data are summarized in Table S3. Proposed product ions are reported in Figure S18 and Table S4.

Lipid A has been characterized in both colistin sensitive colistin resistant variants of *Acinetobacter baumannii* 19606 (9). Notably, three ions at *m/z* of 1910, 1404, and 1728 were identified as the predominant Lipid A species by negative mode MALDI MS with either 3-OH C_12_/3-OH C_12_/3-OH C_12_/3-OH C_14_ or 3-OH C_12_/3-OH C_14_/3-OH C_12_/3-OH C_14_ acyl chain lengths in the conserved Lipid A core. In another paper the structure of *Acinetobacter baumannii* MAC204, which shares the same three lipid A species, a detailed structural characterization of Lipid A was conducted by Fourier transform ion cyclotron resonance mass spectrometry (10). In their MALDI FTICR-MS/MS analysis, the O- and N- linked acyl chains were reported as 3-OH C_12_/3-OH C_14_/3-OH C_12_/3-OH C_14_. Based upon this analysis, it is reasonable to predict that the predominant LPS biosynthetic intermediates in our study should be acyl-chain variants of either 12 or 14 carbons in length. Although we have drawn LPS intermediates with specific acyl-chain locations, consistent with the described material, it should be noted that position (e.g. 3-OH C_12_/3-OH C_14_ or 3-OH C_14_/3-OH C_12_) was not determined in our analysis.

To determine which potential acyl-chain variants were most abundant in our study, we ran a series of precursor ion scans. We used the resulting data to decide which intermediates to target with our sensitive QQQ LC-MRM methodology. By scanning the first quadrupole in our triple quadrupole instrument with the third constant, we were able to identify the precursor which provided the desired daughter ion of 385 *m/z* for the UDP-containing intermediates, or 79 *m/z* for Lipid X, DSMP, and Lipid IV_A_. Sufficient signal could not be seen for any LpxA product in this scan (data not shown). For the LpxC product, the 3-OH C_12_ and 3-OH C_14_ variants were observed to be the most abundant, although this signal was only seen in the LpxH depletion strain (Figure S5A). For the LpxD product, only the 2x 3-OH C_12_ and 1x 3-OH C_12_ 1x 3-OH-C_14_ species provided significant signal, and these were only observable in the LpxH depletion strain (Figure S5B). For Lipid X and the phosphate product ion scan, a strong signal was seen, corresponding to 1 x 3-OH C_12_ 1x 3-OH C_14_ (Figure S5C). Very weak signals, potentially corresponding to 2x 3-OH C_12_ and 2x 3-OH C_14_ were observed, however, the 2x 3-OH-C_12_ species did not produce acceptable data in MRM mode (data not shown). Product ion scan peaks corresponding to DSMP acyl-chain variants of 2x 3-OH C_12_ / 2x 3-OH C_14_ and 3x 3-OH C_12_ / 1x 3-OH-C_14_ provided very robust signals in the LpxH depletion strain (Figure S5D). For Lipid IV_A_, product ion scan peaks corresponding to acyl-chain variants of 2x 3-OH C_12_ / 2x 3-OH C_14_ and 3x 3-OH C_12_ / 1x 3-OH C_14_ provided robust signals (Figure S5D). A third peak potentially corresponding to 1x 3-OH C_12_ / 3x 3-OH C_14_ did not provide acceptable data in MRM mode (data not shown).

Together, these product ion scan data suggest that the most abundant LPS biosynthetic intermediates in the *A. baumannii* lipid A biosynthetic pathway are a mixture of 3-OH C_12_ and 3-OH C_14_ variants. It is not the goal of our study to exhaustively characterize all biosynthetic intermediates, but rather to follow the most abundant, and to observe whether and how these abundant lipid A precursors’ relative levels correlate to biologically important changes. Indeed, within the greater field of lipid chemistry, most biologically active species are found in a distribution of acyl-chain lengths, with the specific variation in acyl-chain length (e.g. +/- a methylene) often having minor effects on physiochemical properties and biological activity.

Our experimental survey of the abundant biosynthetic species was combined with our literature knowledge of the predominant Lipid A intermediates in *Acinetobacter baumannii* to design our MRM scans as described in Table S2. The identities of the intermediates for which we report data were then characterized through a variety of methods as described in the following sections.

**Identification of LpxA products UDP-3-O-[(R)-3-OH-C_14_]-GlcNAc and UDP-3-O-[(R)-3-OH-C_12_]-GlcNAc:**

The 3-OH C_14_ LpxA product (UDP-3-O-[(R)-3-OH-C_14_]-GlcNAc) has been subjected to MS/MS on an AB Sciex 4000 triple quadrupole mass spectrometer as reported in the literature for the synthetic Alberta Research Chemicals material (2). Three of the major fragments in the published spectra are 159 *m/z*, 273 *m/z*, and 385 *m/z*. Indeed these same fragment ions have been described for other UDP-containing metabolites using accurate mass QTOF MS/MS (7). Based upon the characterized final structure of the reported *Acinetobacter baumannii* lipid A (9), while the 3-OH C_12_ LpxA product (UDP-3-O-[(R)-3-OH-C_12_]-GlcNAc) has not been previously characterized, its existence can be inferred. The 159 *m/z*, 273 *m/z* and 385 *m/z* product ions reported for the characterized standard would be expected to be observed from both acyl-chain variants, as they are derived from the conserved UDP-GlcNAc (Figure S18, Table S4). Small differences in acyl-chain lengths do not substantially alter the product ion spectra for LpxA product, as 159 *m/z* was still one of the dominant fragment ions from the m/z 776 parent corresponding to the 3-OH C_10_ *Pseudomonas* LpxA product as determined by analysis of an authentic standard (6).

As observed in the QQQ LC-MRM chromatograms (Figure S6A-B) and the QTOF LC-MS chromatograms (Figures S7A-B) the experimental LpxA product appears to consist of a mixture of the 3-OH C_12_ and 3-OH C_14_ acyl-chain variants, which exhibit similar retention times when compared to the 3-OH C_14_ acyl-chain variant authentic standard (Figures S6C-D, S7C-D). The MS/MS spectra of the samples (Figure S6E) and the standards (Figures S6F, S7E) compare favorably with each other, each containing the dominant UDP derived 159 *m/z*, 273 *m/z*, and 385 *m/z* fragments. Indeed, other UDP-derived fragments could also be assigned (Figure S18, Table S4).

Based upon all of these data, and upon the “Proposed minimum reporting standards for chemical analysis” (11) 3-OH C_14_ LpxA product (UDP-3-O-[(R)-3-OH-C_14_]-GlcNAc) would be identified as a Type I “Identified compound” and 3-OH C_12_ LpxA product (UDP-3-O-[(R)-3-OH-C_12_]-GlcNAc) would be identified as a Type II “Putatively identified compound.”

**Identification of LpxC products UDP-3-O-[(R)-3-OH-C_14_]-GlcN and UDP-3-O-[(R)-3-OH-C_12_]-GlcN:**

The 3-OH C_14_ LpxC product (UDP-3-O-[(R)-3-OH-C_14_]-GlcN) has been subjected to MS/MS on an AB Sciex 4000 triple quadrupole mass spectrometer as reported in the literature for the authentic standard prepared by enzymatic transformation from the synthetic Alberta Research Chemicals material (2). Three of the major fragments in the published spectra are 159 *m/z*, 273 *m/z*, and 385 *m/z*. Indeed, these same fragment ions have been described for other UDP-containing metabolites using QTOF MS/MS (7). Based upon the characterized final structure of the reported *Acinetobacter baumannii* lipid A (9), while the 3-OH C_12_ LpxC product (UDP-3-O-[(R)-3-OH-C_12_]-GlcN) has not been previously characterized, its existence can be inferred. The 159 *m/z*, 273 *m/z* and 385 *m/z* product ions reported for the characterized standard would be expected to be observed for both acyl-chain variants, as they are derived from the conserved UDP-GlcNAc (Figure S18, Table S4). Small differences in acyl-chain lengths do not substantially alter the product ion spectra for LpxC product as 159 *m/z* was still one of the dominant fragment ions from the 734 *m/z* parent, corresponding to the 3-OH C_10_ *Pseudomonas* LpxA product as determined by analysis of an authentic standard (6).

As observed in the QQQ LC-MRM chromatograms (Figure S8A-B) and the QTOF LC-MS chromatograms (Figures S9A-B), the experimental LpxC product appears to consist of a mixture of the 3-OH C_12_ and 3-OH C_14_ acyl-chain variants, which exhibit similar retention times as compared to the 3-OH C_14_ acyl-chain variant authentic standard (Figures S8C-D, S9C-D). The MS/MS spectra of the samples (Figure S8E-F) and the standards (Figures S8G, S9E) compare favorably with each other, each containing the dominant UDP derived 159 *m/z*, 273 *m/z*, and 385 *m/z* fragments. UDP-derived fragments, as well as fragments retaining the acyl-GlcN moiety can be assigned (Figure S18, Table S4). Numerous fragment ions are shared between the acyl chain variants (as expected). Moreover, we observe 28 Da mass shifts due to the C_12_ versus C_14_ acyl chain variants.

Based upon all of these data, and upon the “Proposed minimum reporting standards for chemical analysis” (11) 3-OH-C_14_ LpxC product (UDP-3-O-[(R)-3-OH-C_14_]-GlcN) would be identified as a Type I “Identified compound” and 3-OH-C_12_ LpxA product (UDP-3-O-[(R)-3-OH-C_12_]-GlcN) would be identified as a Type II “Putatively identified compound.”

**Identification of LpxD products (UDP-2,3-diacyl-GlcN):**

The 2x 3-OH C_14_ LpxD product (UDP-2,3-diacyl-GlcN) has been described in the literature on an AB Sciex 4000 triple quadrupole instrument with three dominant fragments present at at 159 *m/z*, 273 *m/z*, and 385 *m/z* (3, 5). Indeed these same fragment ions have been described for other UDP-containing metabolites using QTOF MS/MS (7). Metzger’s reference was obtained by co-purification with catalytically inactive LpxI, of which (UDP-2,3-diacyl-GlcN) is the substrate. Among other methods, the material was characterized by x-ray crystallography bound in the enzyme active site. Based upon the characterized final structure of the reported *Acinetobacter baumannii* lipid A, which includes a mixture of 3-OH C_12_ and 3-OH C_14_ while the 2x 3-OH C_12_ LpxD product (UDP-2,3-diacyl-GlcN) and the 1x 3-OH C_12_ 3-OH C_14_ LpxD product (UDP-2,3-diacyl-GlcN) have not been previously characterized their existence can be inferred. The 159 *m/z*, 273 *m/z* and 385 *m/z* product ions reported for the characterized standard would be expected to be observed from both acyl-chain variants as they are derived from the conserved UDP-GlcNAc (Figure S18, Table S4).

As observed in the QQQ LC-MRM chromatograms (Figure S10A-C) and the QTOF LC-MS chromatograms (Figures S11A-C) the experimental LpxD product appears to consist of predominantly 2x 3-OH-C_12_ and 1x 3-OH-C_12_ 1x 3-OH-C_12_ acyl-chain variants whereas the standard consists of predominantly the 1x 3-OH-C_12_ 1x 3-OH-C_12_ and 2x 3-OH-C_14_ acyl-chain variants each with matching retention times (Figures S10D-F, S11D-F). The MS/MS spectra of the samples (Figure S10G-I, S11G) and the standards (Figures S10J-K, S11H-IE) compare favorably with each other; all but the lowest signal spectra (Figure S10I) containing the dominant UDP derived 159 *m/z*, 273 *m/z*, and 385 *m/z* fragments. Further fragmentation including loss of acyl chains is depicted in Figure S18, Table S4. Notably “Lipid X-like” fragments containing the diacyl-GlcN moiety as well as UDP derived fragments can be assigned. Numerous fragment ions are shared between the acyl chain variants (as expected). Moreover, we observe 28 Da mass shifts due to the C_12_ versus C_14_ acyl chain variants.

Based upon all of these data, and upon the “Proposed minimum reporting standards for chemical analysis” (11) suggest that both experimental LpxD products would be identified as Type I “Identified compounds.”

**Identification of LpxH product (Lipid X):**

The 2x 3-OH C_14_ LpxH product **(**Lipid X) has been described in the literature on an AB Sciex 4000 triple quadrupole instrument with fragments assigned at 79 *m/z*, 97 *m/z*, 240 *m/z*, and 466 *m/z.*^2,3^ Metzger’s reference was obtained by co-purification with catalytically active LpxI, of which (UDP-2,3-diacyl-GlcN) is the product (5). Among other methods, the material was characterized by x-ray crystallography bound in the enzyme active site. Based upon the characterized final structure of the reported *Acinetobacter baumannii* lipid A, which includes a mixture of 3-OH-C_12_ and 3-OH-C_14_ while the 2x 3-OH C_12_ LpxH product (Lipid X) and the 1x 3-OH C_12_ 1x 3-OH C_14_ LpxH product (Lipid X) have not been previously characterized, their existence can be inferred.

As observed in the QQQ LC-MRM chromatograms (Figure S12A-B) and the QTOF LC-MS chromatograms (Figures S13A-B) the experimental Lipid X consists predominantly of the 1x 3-OH-C_12_ 1x 3-OH-C_14_ acyl-chain variant with minor amounts of the 2x 3-OH-C_14_ acyl-chain variants. The 2x 3-OH-C_12_ acyl-chain variant was not detected (data not shown). The standard consists mainly of the 2x 3-OH-C_14_ acyl-chain variant with minor amounts of the 1x 3-OH-C_12_ 1x 3-OH-C_14_ acyl-chain variant with matched retention times (Figures S12C-D, S13C-D). The MS/MS spectra of the samples (Figure S12E) and the standards (Figures S12F, S10E-F) compare favorably, each containing the 79 *m/z*, 97 *m/z*, 240 *m/z*, and 466 *m/z* fragment ions among others (Figure S18, Table S4). It seems likely that the spectra (Figure S13E) may contain a contaminating isobaric species based upon the additional ions present not shared with the other Lipid X species. Numerous fragment ions are shared between the acyl chain variants (as expected). Moreover, we observe 28 Da mass shifts due to the C_12_ versus C_14_ acyl chain variants. Based upon all of these data, and upon the “Proposed minimum reporting standards for chemical analysis” (11) both experimental Lipid X products would be identified as a Type I “Identified compounds.”

**Identification of LpxB products (DSMP):**

A standard of the LpxB product DSMP has not been extensively characterized by mass spectrometric methods in the literature. However, a related penta-acylated DSMP analog, has been analyzed by MS/MS with fragments corresponding to a loss of 78 *m/z* (phosphate) and cleavage of glycosolic bond between the rings reported (Figure S18, Table S4) (8). The MRM analysis of 4x 3-OH C_14_ LpxB product (DSMP) was described by Li (3), with the notably fragment being the loss of an acyl chain (Figure S18, Table S4). Based upon the characterized final structure of the reported *Acinetobacter baumannii* lipid A, which includes a mixture of 3-OH C_12_ and 3-OH C_14_ . The 3x 3-OH C_12_ and 1x 3-OH C_14_ LpxB product (DSMP) and the 2x 3-OH C_12_ and 2x 3-OH C_14_ LpxB products (DSMP) we observe (Figure S5) as the dominant species can be inferred based upon this lipid A structure.

As observed in the QQQ LC-MRM chromatograms (Figure S14A-C) and the QTOF LC-MS chromatograms (Figures S15A-C) the experimental DSMP consists predominantly of the 3x 3-OH C_12_ 1x 3-OH-C_14_ and the 2x 3-OH C_12_ 2x 3-OH C_14_ acyl-chain variants whereas the authentic standard consists of only the 4x 3-OH C_14_ acyl-chain variant (Figure S14D-F, S15D-F). The MS/MS spectra of the samples (Figure S14G-H) and the standards (Figures S14I, S15G) are consistent with the assigned structures. Indeed a series of single and double acyl chain loss species, as well as a glycoside bond cleavage, can be assigned (Figure S18, Table S4). Numerous fragment ions are shared between the acyl chain variants (as expected). Moreover, we observe 28 Da mass shifts due to the C_12_ versus C_14_ acyl chain variants.

Taken together, these data would identify the 3x 3-OH C_12_ 1x 3-OH C_14_ and the 2x 3-OH C_12_ 2x 3-OH C_14_ LpxB products (DSMP) as Type II “Putatively identified compounds.”

**Identification of LpxK product (Lipid IV_A_):**

A 4x 3-OH C_14_ authentic standard of Lipid A has been extensively characterized by Fourier Transform Ion Cyclotron Resonance (FTMS) with various activation methods (4). Based upon the characterized final structure of the reported *Acinetobacter baumannii* lipid A, which includes a mixture of 3-OH C_12_ and 3-OH C_14_ . The 3x 3-OH C_12_ and 1x 3-OH C_14_ LpxB product (DSMP) and the 2x 3-OH C_12_ and 2x 3-OH C_14_ LpxB products (DSMP) we observe (Figure S5) as the dominant species can be inferred based upon this lipid A structure.

As observed in the QQQ LC-MRM chromatograms (Figure S16A-B) and the QTOF LC-MS chromatograms (Figures S17A-C) the experimental Lipid IV_A_ consists predominantly of the 3x 3-OH C_12_ 1x 3-OH C_14_ and the 2x 3-OH C_12_ 2x 3-OH C_14_ acyl-chain variants whereas the authentic standard consists primarily of the 4x 3-OH C_14_ acyl-chain variant (Figure S16C-E, S17D-F). The MS/MS spectra of the samples (Figure S16F-G) and the standards (Figures S16I, S17G) are provided. Three dominant ions previously reported in the literature (4) were observed for our 4x 3-OH C_14_ authentic standard of Lipid A (1305 *m/z*, 1159 *m/z*, 1061 *m/z*) in Table S4. Additionally, the phosphate product ions (97 m/z and 79 *m/z*) are also noted. Unfortunately, our MS/MS spectra were taken at a relatively high collision energy to produce the phosphate product ions, making assignment challenging. Nonetheless, some ions are shared between the Lipid IV_A_ acyl-chain variants whereas others exhibit the 28 Da mass shifts present due to the C_12_ versus C_14_ acyl chain variants. Taken together, these data would identify the 3x 3-OH C_12_ 1x 3-OH C_14_ and the 2x 3-OH C_12_ 2x 3-OH C_14_ LpxK products (IV_A_) as Type II “Putatively identified compounds.”

**Supplemental Methods**

**Preparation of LPS intermediate samples for MS/MS**

Multiple-Reaction-Monitoring (MRM) methodology on triple quadrupole (QQQ) instruments provides some of the most sensitive detection possible for unlabeled non-fluorescent analytes. Unfortunately, our samples as prepared for MRM analysis did not provide sufficient signal for full-scan MS or MS/MS analysis on our QTOF and QQQ instrumentation. To address this, we prepared two concentrated samples for analysis on our less-sensitive non-MRM platforms.

*A. baumannii* NB48062 and NB48062-LMD0007 were grown overnight in MHIIB. The following day, the cells were diluted to 0.01 OD_600_ in 10 ml MHIIB (50 ml conical tube) grown at 37 °C with shaking OD_600_ reached 0.5 done in triplicate for a total of 6 samples (NB48062 plus 16 ug/ml CHIR-090, and NB48062-LMD0007). When the cultures reached 0.5 – 1.0, an aliquot was removed (5 ml), adjusted to an OD_600_ of 0.5, spun down, and the pellet frozen at -80 C.

Two samples were prepared. The first “LpxC” was generated from the NB48062 plus 16 µg/ml CHIR-090 treatment conditions by combining the three replicates. Based upon our previous work, this sample would be expected to show increased amounts of LpxA product (12). The second sample “LpxH minus IPTG” was prepared by growing the NB48062-LMD0007 strain under lpxH depletion conditions as described in the manuscript in the absence of IPTG and then combining the three replicates. The 15 mL total of clarified Solulyse detergent treated cell lysate was then loaded on a Waters Oasis WAX 30 mg SPE cartridge and processed with the standard Waters protocol for strongly acidic compounds. Samples were eluted from the SPE cartridge using a minimal volume of 5% ammonium hydroxide in methanol and then neutralized immediately using 10% formic acid in methanol to prevent base hydrolysis of the analytes. Concentrating the analytes by vacuum centrifuge was avoided as it has been observed to degrade them.

**QQQ LC-MRM and LC-MS/MS of LPS intermediates**

QQQ LC-MRM chromatograms (Figures S6A-D, S8A-D, S10A-F, S12A-D, S14A-F, S16A-E) were acquired using the chromatography and MS settings as described in the main paper text and Table S2 on the LpxC experimental sample, LpxH minus IPTG experimental sample, and all authentic standards.

QQQ LC-MS/MS spectra were acquired using the chromatography and all MS settings as described in our main paper text. Targeted MS/MS product ion scans were obtained for the relevant products in each of our authentic standards. LpxA product QQQ sample MS/MS spectra were acquired from the LpxC experimental sample. All other QQQ sample MS/MS spectra were obtained from the LpxH minus IPTG experimental sample. Full product ion spectra were acquired from 50 *m/z* to at least 50 *m/z* past the calculated 1^-^ parent mass (Figures S6E-F, S8E-G, S10G-K, S12E-F, S14G-I, S16F-I). Collision energies are noted in the figures, along with retention times of the averaged spectra. Average spectra times were determined by comparison with matched QQQ LC-MRM chromatograms of the same samples.

**QTOF LCMS and MS/MS of LPS Intermediates**

QTOF LC-MS chromatograms (Figures S7 A-D, S9A-D, S11A-F, S13A-D, S15A-F, S17A-F) were acquired using the chromatography in the main paper text for the LpxC experimental sample, LpxH minus IPTG experimental sample, and all authentic standards. Our QTOF system has substantially more dead-volume between LC and MS as compared to our QQQ system, resulting <0. 3 minute shifts in retention times. Retention time shifts are consistent for both standards and experimental samples between instruments.

QTOF MS acquisition settings for our Agilent 6550 QTOF included: negative ion mode, dual jet stream source, Gas Temp: 200^o^C, Drying Gas: 14 L/min, Nebulizer 35 PSIG, Sheath Gas Temp 350^o^C, Sheath Gas Flow 11 L/min, V_cap_ 3500 V, Nozzle Voltage 1000 V, Fragmenter 175 V, Oct 1 RF Vpp 750 V, Medium isolation window (4 m/z), Lockmass at 119.0363, MS^1^ Scan 100-3000 m/z at 2 Hz, MS^2^ Scan 50-2500 *m/z* at 3Hz. Displayed chromatograms were extracted at +/-5 ppm mass accuracy. Inset mass spectrum display the spectra at the designated time corresponding to the sample peak. Both the monisotopic peak and isotope peaks are displayed to indicate that the signal monitored is real and that the assigned charge state is correct. The displayed masses were used to calculate exact masses and errors in Table S3. In cases where a distinct chromatographic peak is located with the correct mass at the monoisotopic peak, a putative structural assignment is provided. Please note that acyl chain assignments are for illustrative purposes only, the absolute location of the acyl chains and relative region-isomer ratios were not determined.

QTOF LC-MS/MS spectra were acquired using the chromatography described in our main paper text. Targeted MS/MS product ion scans were obtained for the relevant products in each of our authentic standards. LpxD product sample MS/MS spectra were acquired from the LpxH minus IPTG experimental sample. Full product ion spectra were acquired from 50 *m/z* to 2,500 *m/z* as depicted in Figures (Figures S7E, S9E, S11 G-I, S13E-F, S15G, S17G). Collision energies are noted in the figures, along with retention times of the averaged spectra. Attempts were made to acquire spectra at similar relative collision energies between our Agilent and AB Sciex instrumentation—although this is challenging. Averaged spectra times were determined by comparison with QTOF LC-MS chromatograms of the same samples.

**Preparation of authentic standards for LC-MS and LC-MS/MS analysis:**

The 3-OH-C_14_ LpxA product (UDP-3-O-[(R)-3-OH-C_14_]-GlcNAc) was purchased from Alberta Research Chemicals as previously described (2). The 3-OH-C_14_ LpxC product (UDP-3-O-[(R)-3-OH-C_14_]-GlcN) was prepared from the commercial Alberta Research Chemical material through chemoenzymatic transformation with the *E. coli* LpxC as previously described (2). The 2x 3-OH C_14_ LpxD product (UDP-2,3-diacyl-GlcN) was prepared with Metzger’s procedure as previously described (5). Briefly, UDP-2,3-diacyl-GlcN was co-purified with a protein preparation of catalytically inactive *Caulobacter crescentus* LpxI (having an active site D to A mutation that prevents it from hydrolyzing UDP-2,3-diacyl-GlcN), of which it is the substrate. The LpxI-D225A protein was heterologously over-expressed and purified essentially as described (5), but with the following modification: purification of the protein was halted after the elution of LpxI from an immobilized metal affinity column. UDP-2,3-diacyl-GlcN was then liberated from the partially purified protein by converting the latter into acidic Bligh-Dyer^13^ single-phase, and removing the precipitated protein by centrifugation. The 2x 3-OH C_14_ LpxH product **(**Lipid X) was also prepared by extraction from LpxI, but was isolated from a preparation of the wild-type enzyme (5). Wild-type LpxI was partially purified, as described above, and was then extracted using an acidic two-phase Bligh-Dyer system(13). The lipid X thereby liberated from the LpxI partitioned to the lower (CHCl_3_) phase. DSMP was produced by treating commercially available Lipid IV_A_ with *E. coli* membranes in which LpxF was over-expressed, using assay conditions previously described for this enzyme (8). Commercial Lipid IV_A_ was purchased from Peptides International (CLP-24006-s).

**References**

1. Whitfield C, Trent MS. Biosynthesis and export of bacterial lipopolysaccharides. Annual review of biochemistry. 2014;83:99-128.

2. Langsdorf EF, Malikzay A, Lamarr WA, Daubaras D, Kravec C, Zhang R, et al. Screening for antibacterial inhibitors of the UDP-3-O-(R-3-hydroxymyristoyl)-N-acetylglucosamine deacetylase (LpxC) using a high-throughput mass spectrometry assay. Journal of biomolecular screening. 2010;15(1):52-61.

3. Li C, Guan Z, Liu D, Raetz CR. Pathway for lipid A biosynthesis in Arabidopsis thaliana resembling that of Escherichia coli. Proceedings of the National Academy of Sciences of the United States of America. 2011;108(28):11387-92.

4. Madsen JA, Cullen TW, Trent MS, Brodbelt JS. IR and UV photodissociation as analytical tools for characterizing lipid A structures. Analytical chemistry. 2011;83(13):5107-13.

5. Metzger LEt, Lee JK, Finer-Moore JS, Raetz CR, Stroud RM. LpxI structures reveal how a lipid A precursor is synthesized. Nature structural & molecular biology. 2012;19(11):1132-8.

6. Murphy-Benenato KE, Olivier N, Choy A, Ross PL, Miller MD, Thresher J, et al. Synthesis, Structure, and SAR of Tetrahydropyran-Based LpxC Inhibitors. ACS medicinal chemistry letters. 2014;5(11):1213-8.

7. Vemula H, Bobba S, Putty S, Barbara JE, Gutheil WG. Ion-pairing liquid chromatography-tandem mass spectrometry-based quantification of uridine diphosphate-linked intermediates in the Staphylococcus aureus cell wall biosynthesis pathway. Analytical biochemistry. 2014;465:12-9.

8. Wang X, McGrath SC, Cotter RJ, Raetz CR. Expression cloning and periplasmic orientation of the Francisella novicida lipid A 4'-phosphatase LpxF. The Journal of biological chemistry. 2006;281(14):9321-30.

9. Beceiro A, Llobet E, Aranda J, Bengoechea JA, Doumith M, Hornsey M, et al. Phosphoethanolamine modification of lipid A in colistin-resistant variants of Acinetobacter baumannii mediated by the pmrAB two-component regulatory system. Antimicrobial agents and chemotherapy. 2011;55(7):3370-9.

10. Mark R. Pelletier LGC, Jace W. Jones, Mark D. Adams, Daniel V. Zurawski, Karsten R. O. Hazlett, Yohei Doi, Robert K. Ernsta. Unique Structural Modifications Are Present in the Lipopolysaccharide from Colistin-Resistant Strains of Acinetobacter baumannii. Antimicrobial agents and chemotherapy. 2013;57(10):4831–40.

11. Sumner LW, Amberg A, Barrett D, Beale MH, Beger R, Daykin CA, et al. Proposed minimum reporting standards for chemical analysis Chemical Analysis Working Group (CAWG) Metabolomics Standards Initiative (MSI). Metabolomics : Official journal of the Metabolomic Society. 2007;3(3):211-21.

12. Bojkovic J, Richie DL, Six DA, Rath CM, Sawyer WS, Hu Q, et al. Characterization of an Acinetobacter baumannii lptD Deletion Strain: Permeability Defects and Response to Inhibition of Lipopolysaccharide and Fatty Acid Biosynthesis. Journal of bacteriology. 2016;198(4):731-41.

13. WJ BEaD. A rapid method for total lipid extraction and purification. Canadian Journal of Biochemistry and Physiology. 1959;37(8):911-7.
