# Supplementary material for: Toxic Accumulation of LPS Pathway Intermediates Underlies the Requirement of LpxH for Growth of Acinetobacter baumannii ATCC 19606
Source: PLoS One. 2016 Aug 15;11(8):e0160918. doi: 10.1371/journal.pone.0160918 (PMC4985137; doi:10.1371/journal.pone.0160918)
Supplement: S1 Table — (PDF) [file pone.0160918.s022.pdf]

Table S1. Oligonucleotide primers used in this study

Primer designation	Primer Sequence (5' - 3')	Application
**KTT858**	gcctttttgcgtttctacaaactctgaagttcctattctcttgaaagtatag gaacttctaccgagctcgaattgacataagcctg	PCR amplification of GentR marker with FRT sites for DNA SOEing of *alaS::Gm*^{R} deletion construct
**KTT859**	cctcgattactgcgatgtttagttaagaagttcctatactttcaagagaa taggaacttccgaattgttaggtggcggtacttg	PCR amplification of GentR marker with FRT sites for DNA SOEing of *alaS::Gm*^{R} deletion construct
**KTT933**	acattaatagtaggtacccgaattgag	PCR amplification of the upstream region of *alaS* for DNA SOEing of *alaS::Gm*^{R} deletion construct
**KTT934**	agagtttgtagaaacgcaaaaaggcaaccaagtcactccatattttcg aatttg	PCR amplification of the upstream region of *alaS* for DNA SOEing of alaS::*alaS::Gm*^{R} deletion construct
**KTT935**	ttaactaaacatcgcagtaatcgagggacttcactttttgtgtaactgac cc	PCR amplification of the downstream region of *alaS* for DNA SOEing of *alaS::Gm*^{R} deletion construct
**KTT936**	tacggcagccaaacattaaacatag	PCR amplification of the downstream region of *alaS* for DNA SOEing of *alaS::Gm*^{R} deletion construct
**cPCR Ab.alaS F**	catctgttacagccataatttctgc	Sequencing of *alaS* region on chromosome
**cPCR Ab.alaS R**	tgatttcacgaccagttttcttacc	Sequencing of *alaS* region on chromosome
**KTT927**	cttctcgatttgtagataaggataggtctttgtttatttttctaaatacattc aaatatg	PCR amplification of *bla* gene for generating pNOV108
**KTT928**	ctacagggcgcgtaaatcaatctaaagtcaatctaaagtatatatgag taaacttggtc	PCR amplification of *bla* gene for generating pNOV108
**KTT929**	ctttagattgatttacgcgccctgtag	PCR amplification of *E. coli* ori pBR322, *Acinetobacter* ori**, and *lacI* for generating pNOV108
**KTT930**	ggaacaactatggcattaatcgttccaggcttatgtcaattcgagctcg	PCR amplification of *E. coli* ori pBR322, *Acinetobacter* ori**, and *lacI* for generating pNOV108
**KTT931**	gaacgattaatgccatagttgttcc	PCR amplification of *alaS* for generating pNOV108
**KTT932**	gacctatccttatctacaaatcgagaag	PCR amplification of *alaS* for generating pNOV108
**KTT939**	tcagaagaactcgtcaagaaggc	PCR amplification of KanR marker for DNA SOEing of IPTG inducible *lpxH* construct
**KTT940**	gacagcaagcgaaccggaattgc	PCR amplification of KanR marker for DNA SOEing of IPTG inducible *lpxH* construct
**KTT941**	gcaattccggttcgcttgctgtccctgaattgactctcttccggg	PCR amplification of Ptac promoter for DNA SOEing of IPTG inducible *lpxH* construct
**KTT942**	gaattctgtttcctgtgtgaaattgtt	PCR amplification of Ptac promoter for DNA SOEing of IPTG inducible *lpxH* construct
**KTT945**	catactcttgttcttccgcatccg	PCR amplification of upstream region of *lpxH* for DNA SOEing of IPTG inducible *lpxH* construct
**KTT946**	gccttcttgacgagttcttctgaagctttatcctaaaattattcagaaata attttagc	PCR amplification of upstream region of *lpxH* for DNA SOEing of IPTG inducible *lpxH* construct
**KTT947**	aacaatttcacacaggaaacagaattcgtgacttatctgtttatttcaga tttacac	PCR amplification of *lpxH* CDS for DNA SOEing of IPTG inducible *lpxH* construct
**KTT948**	tctttggcatcatacgcgattttag	PCR amplification of *lpxH* CDS for DNA SOEing of IPTG inducible *lpxH* construct
**cPCR lpxH F**	ataaccggaagcataacgtggttc	Sequencing of *lpxH* region on chromosome
**cPCR lpxH R**	cgtacgctttggtagtgtagcgac	Sequencing of *lpxH* region on chromosome

*SOEing – Splicing by Overlap Extension;
** This origin fragment was amplified from Acinetobacter sp ATCC23237 which is identical to the origin in plasmid pWH1266 and can replicate in A. baumannii.
