# Supplementary material for: Toxic Accumulation of LPS Pathway Intermediates Underlies the Requirement of LpxH for Growth of Acinetobacter baumannii ATCC 19606
Source: PLoS One. 2016 Aug 15;11(8):e0160918. doi: 10.1371/journal.pone.0160918 (PMC4985137; doi:10.1371/journal.pone.0160918)
Supplement: S2 Table — Lipid A pathway intermediates are described with acyl chain variants. Pathway enzymes producing each intermediate are also listed. Charge of the targeted transition, precursor ions, product ions, and collision energies are noted. (PDF) [file pone.0160918.s023.pdf]

Lipid A Pathway Intermediates	Fatty acid chain length	Product of	Charge	Precursor ion	Product ion	Collision energy (volts)
UDP-3-*O*-[(*R*)-3-OH-C_{12}]-GlcNAc	1 acyl group 12:0(3-OH)	LpxA	1-	804.3	158.9	-80
UDP-3-*O*-[(*R*)-3-OH-C_{12}]-GlcNAc	1 acyl group 12:0(3-OH)	LpxA	1-	804.3	273	-70
UDP-3-*O*-[(*R*)-3-OH-C_{12}]-GlcNAc	1 acyl group 12:0(3-OH)	LpxA	1-	804.3	385	-50
UDP-3-*O*-[(*R*)-3-OH-C_{14}]-GlcNAc	1 acyl group 14:0(3-OH)	LpxA	1-	832.3	158.9	-80
UDP-3-*O*-[(*R*)-3-OH-C_{14}]-GlcNAc	1 acyl group 14:0(3-OH)	LpxA	1-	832.3	273	-70
UDP-3-*O*-[(*R*)-3-OH-C_{14}]-GlcNAc	1 acyl group 14:0(3-OH)	LpxA	1-	832.3	385	-50
UDP-3-*O*-[(*R*)-3-OH-C_{12}]-GlcN	1 acyl group 12:0(3-OH)	LpxC	1-	762.3	158.9	-85
UDP-3-*O*-[(*R*)-3-OH-C_{12}]-GlcN	1 acyl group 12:0(3-OH)	LpxC	1-	762.3	273	-65
UDP-3-*O*-[(*R*)-3-OH-C_{12}]-GlcN	1 acyl group 12:0(3-OH)	LpxC	1-	762.3	385	-52
UDP-3-*O*-[(*R*)-3-OH-C_{12}]-GlcN	1 acyl group 12:0(3-OH)	LpxC	1-	762.3	546.2	-44
UDP-3-*O*-[(*R*)-3-OH-C_{14}]-GlcN	1 acyl group 14:0(3-OH)	LpxC	1-	790.3	158.9	-85
UDP-3-*O*-[(*R*)-3-OH-C_{14}]-GlcN	1 acyl group 14:0(3-OH)	LpxC	1-	790.3	273	-65
UDP-3-*O*-[(*R*)-3-OH-C_{14}]-GlcN	1 acyl group 14:0(3-OH)	LpxC	1-	790.3	385	-52
UDP-3-*O*-[(*R*)-3-OH-C_{14}]-GlcN	1 acyl group 14:0(3-OH)	LpxC	1-	790.3	546.2	-44
UDP-2,3-diacyl-GlcN	2 acyl groups, 12:0(3-OH)	LpxD	1-	960.5	158.9	-78
UDP-2,3-diacyl-GlcN	2 acyl groups, 12:0(3-OH)	LpxD	1-	960.5	273	-64
UDP-2,3-diacyl-GlcN	2 acyl groups, 12:0(3-OH)	LpxD	1-	960.5	385	-60
UDP-2,3-diacyl-GlcN	1 acyl group 12:0(3-OH), 1 acyl group 14:0(3-OH)	LpxD	1-	988.5	158.9	-78
UDP-2,3-diacyl-GlcN	1 acyl group 12:0(3-OH), 1 acyl group 14:0(3-OH)	LpxD	1-	988.5	273	-64
UDP-2,3-diacyl-GlcN	1 acyl group 12:0(3-OH), 1 acyl group 14:0(3-OH)	LpxD	1-	988.5	385	-60
Lipid X	1 acyl group 12:0(3-OH), 1 acyl group 14:0(3-OH)	LpxH	1-	682.4	240.1	-130
Lipid X	1 acyl group 12:0(3-OH), 1 acyl group 14:0(3-OH)	LpxH	1-	682.4	438.2	-63
Lipid X	2 acyl groups, 14:0(3-OH)	LpxH	1-	710.4	240.1	-130
Lipid X	2 acyl groups, 14:0(3-OH)	LpxH	1-	710.4	438.2	-63
DSMP (Disaccharide-1-P)	3 acyl groups 12:0(3-OH), 1 acyl group 14:0(3-OH)	LpxB	1-	1239.9	79	-88
DSMP (Disaccharide-1-P)	3 acyl groups 12:0(3-OH), 1 acyl group 14:0(3-OH)	LpxB	1-	1239.9	807.5	-45
DSMP (Disaccharide-1-P)	3 acyl groups 12:0(3-OH), 1 acyl group 14:0(3-OH)	LpxB	1-	1239.9	1023.7	90
DSMP (Disaccharide-1-P)	2 acyl groups 12:0(3-OH), 2 acyl group 14:0(3-OH)	LpxB	1-	1267.9	79	-88
DSMP (Disaccharide-1-P)	2 acyl groups 12:0(3-OH), 2 acyl group 14:0(3-OH)	LpxB	1-	1267.9	807.5	-45
DSMP (Disaccharide-1-P)	2 acyl groups 12:0(3-OH), 2 acyl group 14:0(3-OH)	LpxB	1-	1267.9	835	-45
DSMP (Disaccharide-1-P)	2 acyl groups 12:0(3-OH), 2 acyl group 14:0(3-OH)	LpxB	1-	1267.9	1023.7	-90
DSMP (Disaccharide-1-P)	2 acyl groups 12:0(3-OH), 2 acyl group 14:0(3-OH)	LpxB	1-	1267.9	1052.7	-90
Lipid IV_A	3 acyl groups 12:0(3-OH), 1 acyl group 14:0(3-OH)	LpxK	1-	1319.9	79	-130
Lipid IV_A	3 acyl groups 12:0(3-OH), 1 acyl group 14:0(3-OH)	LpxK	2-	659.45	79	-45
Lipid IV_A	3 acyl groups 12:0(3-OH), 1 acyl group 14:0(3-OH)	LpxK	2-	659.45	214.2	-50
Lipid IV_A	3 acyl groups 12:0(3-OH), 1 acyl group 14:0(3-OH)	LpxK	2-	659.45	242.2	-35
Lipid IV_A	3 acyl groups 12:0(3-OH), 1 acyl group 14:0(3-OH)	LpxK	2-	659.45	438.2	-35
Lipid IV_A	3 acyl groups 12:0(3-OH), 1 acyl group 14:0(3-OH)	LpxK	2-	659.45	1023.6	-40
Lipid IV_A	2 acyl groups 12:0(3-OH), 2 acyl groups 14:0(3-OH)	LpxK	1-	1347.9	79	-130
Lipid IV_A	2 acyl groups 12:0(3-OH), 2 acyl groups 14:0(3-OH)	LpxK	2-	673.45	79	-45
Lipid IV_A	2 acyl groups 12:0(3-OH), 2 acyl groups 14:0(3-OH)	LpxK	2-	673.45	214.2	-50
Lipid IV_A	2 acyl groups 12:0(3-OH), 2 acyl groups 14:0(3-OH)	LpxK	2-	673.45	242.2	-35
Lipid IV_A	2 acyl groups 12:0(3-OH), 2 acyl groups 14:0(3-OH)	LpxK	2-	673.45	438.2	-35
Lipid IV_A	2 acyl groups 12:0(3-OH), 2 acyl groups 14:0(3-OH)	LpxK	2-	673.45	1023.6	-40
