# Supplementary material for: Toxic Accumulation of LPS Pathway Intermediates Underlies the Requirement of LpxH for Growth of Acinetobacter baumannii ATCC 19606
Source: PLoS One. 2016 Aug 15;11(8):e0160918. doi: 10.1371/journal.pone.0160918 (PMC4985137; doi:10.1371/journal.pone.0160918)
Supplement: S3 Table — Lipid A pathway intermediates are described with acyl chain variants. Retention times by QQQ MRM analysis are noted, with standard peaks within 0.2 minutes of experimental peaks. Differences may be due to matrix effects for the poorly behaved detergent like molecules. QTOF theoretical and experimental monoisotopic m/z values are reported with mass errors in parts per million (ppm). Mass errors are <5 ppm for all species detected in the QTOF. Retention times by QTOF LC-MS are noted, with standard peaks within 0.2 minutes of experimental peaks. Differences may be due to matrix effects for the poorly behaved detergent like molecules. Peak times differ between QTOF and QQQ systems due to substantially larger dead-volume in the QTOF system. The relatively poor behavior observed for lipid IVA in the QTOF system may also relate to interaction with the plastic surfaces in our Agilent system. Our AB Sciex system uses all glass and metal surfaces post-column. (PDF) [file pone.0160918.s024.pdf]

**Lipid A Pathway Intermediates**	**Fatty acid chain length**	**Product of**	**Charge**	**QQQ precursor ion (*m/z*)**	**QQQ tret experimental (min)**	**QQQ tret standard (min)**	**QTOF *m/z* theoretical**	**QTOF *m/z* experimental**	**QTOF experimental mass error (ppm)**	**QTOF *m/z* standard**	**QTOF standard mass error (ppm)**	**QTOF tret experimental (min)**	**QTOF tret standard (min)**
UDP-3-*O*-[(*R*)-3-OH-C_{12}]-GlcNAc	1 acyl group 12:0(3-OH)	LpxA	1-	804.3	2.8	-	804.2362	804.2361	0.1	-	-	3.1	-
UDP-3-*O*-[(*R*)-3-OH-C_{14}]-GlcNAc	1 acyl group 14:0(3-OH)	LpxA	1-	832.3	3.0	3.0	832.2675	832.2632	5.2	832.2701	-3.1	3.3	3.3
UDP-3-*O*-[(*R*)-3-OH-C_{12}]-GlcN	1 acyl group 12:0(3-OH)	LpxC	1-	762.3	2.7	2.9	762.2257	762.2243	1.8	-	-	3.2	-
UDP-3-*O*-[(*R*)-3-OH-C_{14}]-GlcN	1 acyl group 14:0(3-OH)	LpxC	1-	790.3	2.9	3.1	790.2570	790.2556	1.8	790.2576	-0.8	3.4	3.4
UDP-2,3-diacyl-GlcN	2 acyl groups, 12:0(3-OH)	LpxD	1-	960.5	3.3	3.3	960.3877	960.3889	-1.2	960.3853	2.5	3.8	3.6
UDP-2,3-diacyl-GlcN	1 acyl group 12:0(3-OH), 1 acyl group 14:0(3-OH)	LpxD	1-	988.5	3.4	3.4	988.4190	988.4196	-0.6	988.4188	0.2	3.8	3.7
UDP-2,3-diacyl-GlcN	2 acyl groups, 14:0(3-OH)	LpxD	1-	1016.5	3.5	3.5	1,016.4503	-	-	1,016.4517	-1.4	-	3.8
Lipid X	1 acyl group 12:0(3-OH), 1 acyl group 14:0(3-OH)	LpxH	1-	682.4	3.7	3.6	682.3937	682.3915	3.2	682.3932	0.7	4.1	3.9
Lipid X	2 acyl groups, 14:0(3-OH)	LpxH	1-	710.4	3.8	3.7	710.4250	-	-	710.4262	-1.7	-	4.0
DSMP (Disaccharide-1-P)	3 acyl groups 12:0(3-OH), 1 acyl group 14:0(3-OH)	LpxB	1-	1239.9	4.1	-	1,239.7864	1,239.7831	2.7	-	-	4.4	-
DSMP (Disaccharide-1-P)	2 acyl groups 12:0(3-OH), 2 acyl group 14:0(3-OH)	LpxB	1-	1267.9	4.1	4.1	1,267.8177	1,267.8147	2.4	-	-	4.4	-
DSMP (Disaccharide-1-P)	4 acyl group 14:0(3-OH)	LpxB	1-	1323.9	-	4.2	1,323.8803	-	-	1323.8808	-0.4	-	4.4
Lipid IV_A	3 acyl groups 12:0(3-OH), 1 acyl group 14:0(3-OH)	LpxK	1-	1319.9	4.2	4.2	1,319.7528	-	-	-	-	-	-
Lipid IV_A	3 acyl groups 12:0(3-OH), 1 acyl group 14:0(3-OH)	LpxK	2-	659.45	4.2	4.2	659.3728	-	-	-	-	-	-
Lipid IV_A	2 acyl groups 12:0(3-OH), 2 acyl groups 14:0(3-OH)	LpxK	1-	1347.9	4.2	4.2	1,347.7841	-	-	-	-	-	-
Lipid IV_A	2 acyl groups 12:0(3-OH), 2 acyl groups 14:0(3-OH)	LpxK	2-	673.45	4.2	4.2	673.3884	-	-	-	-	-	-
Lipid IV_A	4 acyl groups 14:0(3-OH)	LpxK	1-	1403.9	-	4.1	1,403.8467	-	-	1403.8464	0.2	-	4.3
Lipid IV_A	4 acyl groups 14:0(3-OH)	LpxK	2-	701.4	-	4.1	701.4197	-	-	701.4211	-2.0	-	4.3
