# Supplementary material for: Toxic Accumulation of LPS Pathway Intermediates Underlies the Requirement of LpxH for Growth of Acinetobacter baumannii ATCC 19606
Source: PLoS One. 2016 Aug 15;11(8):e0160918. doi: 10.1371/journal.pone.0160918 (PMC4985137; doi:10.1371/journal.pone.0160918)
Supplement: S4 Table — Lipid A pathway intermediates are described. QQQ precursor m/z or QTOF theoretical m/z are provided depending on where the specific spectra was acquired—this is also noted in the reported precision. Product ions of interest are indicated and if they can be assigned to a putative structure as in S18 Fig, a theoretical exact m/z is provided. Peaks are also noted if a) shared between acyl chain variants (e.g. UDP fragments of LpxD product) or b) shifted by 28 Da between acyl chain variants (e.g. acyl-GlcN fragments form LpxD product) with the second class in bold. Peaks matching a specific MRM transition are italic underlined as reported in S4 Table. MRM transitions were determined by analysis of authentic standards, however, not all MRM transitions were apparent as product ion spectra were collected at a single collision energy rather than by the varied collision energies used in the initial MRM (S4 Table). (PDF) [file pone.0160918.s025.pdf]

Lipid A Pathway Intermediates	Fatty acid chain length	Product of	Charge	QQQ precursor ion (*m/z*)	QTOF *m/z* theoretical	Spectra																						
UDP-3-*O*-[(*R*)-3-OH-C_{12}]-GlcNAc	1 acyl group 12:0(3-OH)	LpxA	1-	804.3	-	Experimental	403.7	*385.5*	343.9	322.8	*272.7*	177.0	*159.5*	97.0	79.2													
UDP-3-*O*-[(*R*)-3-OH-C_{14}]-GlcNAc	1 acyl group 14:0(3-OH)	LpxA	1-	832.3	-	Standard	403.6	*384.7*	344.0	322.1	*272.7*	177.0	*158.8*	97.6	78.9													
UDP-3-*O*-[(*R*)-3-OH-C_{14}]-GlcNAc	1 acyl group 14:0(3-OH)	LpxA	1-	-	832.2675	Standard	402.9941	*384.9845*	343.9935	-	*272.9566*	176.9536	*158.9255*	96.9695	78.9590													
				-	-	Theoretical	402.9949	384.9844	-	-	-	176.9359	158.9254	96.9696	78.9590													
UDP-3-*O*-[(*R*)-3-OH-C_{12}]-GlcN	1 acyl group 12:0(3-OH)	LpxC	1-	762.3	-	Experimental	-	-	-	**456.4**	403.1	*385.3*	323.1	*273.7*	-	*159.0*	-	-										
UDP-3-*O*-[(*R*)-3-OH-C_{14}]-GlcN	1 acyl group 14:0(3-OH)	LpxC	1-	790.3	-	Experimental	*546.4*	**484.0**	-	-	402.8	*385.2*	-	*273.4*	-	*159.3*	-	-										
UDP-3-*O*-[(*R*)-3-OH-C_{14}]-GlcN	1 acyl group 14:0(3-OH)	LpxC	1-	790.3	-	Standard	*546.5*	**484.9**	**466.7**	-	403.7	*385.3*	323.4	*273.3*	240.5	*159.1*	96.7	79.3										
UDP-3-*O*-[(*R*)-3-OH-C_{14}]-GlcN	1 acyl group 14:0(3-OH)	LpxC	1-	790.3	790.2570	Standard	*546.1873*	-	-	-	402.9947	*384.9849*	323.0288	*272.9574*	240.0276	*158.9255*	96.9699	78.9591										
				-	-	Theoretical	-	484.2317	466.2211	456.2004	402.9949	384.9844	323.0286		240.0279	158.9254	96.9696	78.9590										
UDP-2,3-diacyl-GlcN	2 acyl groups, 12:0(3-OH)	LpxD	1-	960.5		Experimental	-	-	-	-	**655.0**	**637.4**	-	403.1	*385.3*	323.4	*273.6*	215.2	177.4	*159.5*	111.0	-	-						
UDP-2,3-diacyl-GlcN	1 acyl group 12:0(3-OH), 1 acyl group 14:0(3-OH)	LpxD	1-	988.5		Experimental	-	-	**683.4**	**665.1**	-	-	466.8	403.5	*385.4*	323.6	*273.5*	215.8	177.4	-	-	-	-						
UDP-2,3-diacyl-GlcN	1 acyl group 12:0(3-OH), 1 acyl group 14:0(3-OH)	LpxD	1-		988.4190	Experimental	-	-	**682.3943**	**664.3839**	-	-	466.2216	402.9954	*384.9852*	323.0286	*272.9574*	-	176.9357	*158.9253*	-	96.9697	78.9590						
UDP-2,3-diacyl-GlcN	1 acyl group 12:0(3-OH), 1 acyl group 14:0(3-OH)	LpxD	1-	988.5		Standard	-	-	-	-	-	-		403.8	*385.8*	323.6	*273.0*	-	177.6	*159.0*	-								
UDP-2,3-diacyl-GlcN	1 acyl group 12:0(3-OH), 1 acyl group 14:0(3-OH)	LpxD	1-	988.4190		Standard	-	-	**682.4**	**664.4**	-	-	466.2	403.0	*385.0*	323.0	*273.0*	-	176.9	*158.9*	-	97.0	79.0						
UDP-2,3-diacyl-GlcN	2 acyl groups, 14:0(3-OH)	LpxD	1-	1016.5		Experimental	**711.5**	**693.3**	-	-	-	-		403.5	*385.8*	-		-	-	-	-	79.4	-						
UDP-2,3-diacyl-GlcN	2 acyl groups, 14:0(3-OH)	LpxD	1-	1016.5		Standard	**711.3**	**692.9**	-	-	-	-	467.0	403.2	*385.7*	322.9	*273.4*	-	-	*159.1*	-	-	-						
UDP-2,3-diacyl-GlcN	2 acyl groups, 14:0(3-OH)	LpxD	1-		1,016.4503	Standard	**710.4220**	**692.4111**	-	-	-	-	466.2194	402.9933	*384.9832*	323.0276	*272.9594*	-	176.9249	*158.9249*	-	96.9698	78.9589						
				-	-	Theoretical	710.4250	692.4144	682.3937	664.3831	654.3624	636.3518	466.2211	402.9949	384.9844	323.0286		215.1652	176.9359	158.9254	111.0200	96.9696	78.9590						
Lipid X	1 acyl group 12:0(3-OH), 1 acyl group 14:0(3-OH)	LpxH	1-	682.4		Experimental	**466.8**	-	*240.1*	96.9	79.1																	
Lipid X	1 acyl group 12:0(3-OH), 1 acyl group 14:0(3-OH)	LpxH	1-		682.3937	Standard	**466.2204**	***438.1895***	*240.0272*	96.9682	78.9589																	
Lipid X	2 acyl groups, 14:0(3-OH)	LpxH	1-	710.4		Standard	**466.8**	-	*239.8*	96.9	79.3																	
Lipid X	2 acyl groups, 14:0(3-OH)	LpxH	1-		710.4250	Standard	**466.2216**	-	*240.0279*	96.9696	78.9590																	
				-	-	Theoretical	466.2211	438.1898	240.0279	96.9696	79.9663																	
DSMP (Disaccharide-1-P)	3 acyl groups 12:0(3-OH), 1 acyl group 14:0(3-OH)	LpxB	1-	1239.9		Experimental	-	-	-	***1,024.9***	-	-	***808.7***	-	-	-	652.6	-	-	448.5	-	97.0	-						
DSMP (Disaccharide-1-P)	2 acyl groups 12:0(3-OH), 2 acyl group 14:0(3-OH)	LpxB	1-	1267.9		Experimental	-	-	***1,052.6***	-	-	***836.2***	-	-	-	**725.1**	651.9	-	-	448.3	-	-	-						
DSMP (Disaccharide-1-P)	4 acyl group 14:0(3-OH)	LpxB	1-	1323.9		Standard	**1.099.1**	**1.080.8**	-	-	854.7	836.3	-	795.5	**753.7**	-	652.2	593.0	467.0	449.1	-	-	79.8						
DSMP (Disaccharide-1-P)	4 acyl group 14:0(3-OH)	LpxB	1-	1323.9	1,323.8803	Standard	1.097.6830	1.079.6783	-	-	853.4816	835.4713		794.4439	752.4343	-	-	592.2521		448.2095	242.2126	96.9699	78.9590						
						Theoretical	1,097.6871	1,079.6765	1,051.6452	1,023.5927	853.4832	835.4726	807.4410						466.2211		243.1965	96.9696	79.9663						
Lipid IVa	3 acyl groups 12:0(3-OH), 1 acyl group 14:0(3-OH)	LpxK	1-	1319.9		Experimental	-	-	-	-	-	-	-	-	-	-	-	-	**381.2**	363.9	-	-	253.7	227.2	211.5	153.2	-	-
Lipid IVa	2 acyl groups 12:0(3-OH), 2 acyl groups 14:0(3-OH)	LpxK	1-	1347.9		Experimental	-	-	-	-	-	646.1	-	-	**409.9**	-	392.0	364.0	-	-	281.5	268.2	256.0	227.6	211.6	153.4	-	*79.5*
Lipid IVa	2 acyl groups 12:0(3-OH), 2 acyl groups 14:0(3-OH)	LpxK	1-	1347.9		Standard	-	-	-	-	-	646.3	-	466.7	**409.9**	-	392.2	363.3	-	-	-	267.8	255.8	227.3	211.3	153.9	-	*79.8*
Lipid IVa	4 acyl groups 14:0(3-OH)	LpxK	1-	1403.9		Standard	-	-	-	688.8	-	646.5	-	-	**409.9**	403.9	392.1	-	-	-	281.9	-	256.0	-	-	153.7	97.1	-
Lipid IVa	4 acyl groups 14:0(3-OH)	LpxK	1-		1,403.8467	Standard	1,305.8671	1,159.6406	1,061.6656	687.4963	659.4605	-	483.2002	-	-	403.2239	391.2232	-	-	-	281.2471	267.3340	255.2321	-	211.0637	152.9954	-	-
						Ref*/Theo.	1305.92*	1159.67*	1061.75*					466.2211													96.9696	79.9663
