# Supplementary material for: Toxic Accumulation of LPS Pathway Intermediates Underlies the Requirement of LpxH for Growth of Acinetobacter baumannii ATCC 19606
Source: PLoS One. 2016 Aug 15;11(8):e0160918. doi: 10.1371/journal.pone.0160918 (PMC4985137; doi:10.1371/journal.pone.0160918)
Supplement: S17 Fig — Chromatograms are provided for Lipid IVa acyl chain variants from both experimental samples and authentic standards. The specific Extracted Ion Chromatogram (EIC) being monitored as described in S3 Table is noted. Retention times are annotated. Peaks are labeled with (MS/MS) if product ion spectra were obtained for the specific peak. QTOF MS/MS spectra are displayed with peaks annotated. Product ion peaks are summarized in S4 Table and putative structural assignments are made in S18 Fig. In cases where a chromatographic peak is observed a proposed structure is provided. Acyl chain positions are for illustrative purposes only, based upon the final Lipid A structure. Our analysis does not clarify whether the species is C12 / C14 / C12 / C14 or C12 / C12 / C14 / C14, or a mixture of these. (PDF) [file pone.0160918.s017.pdf]

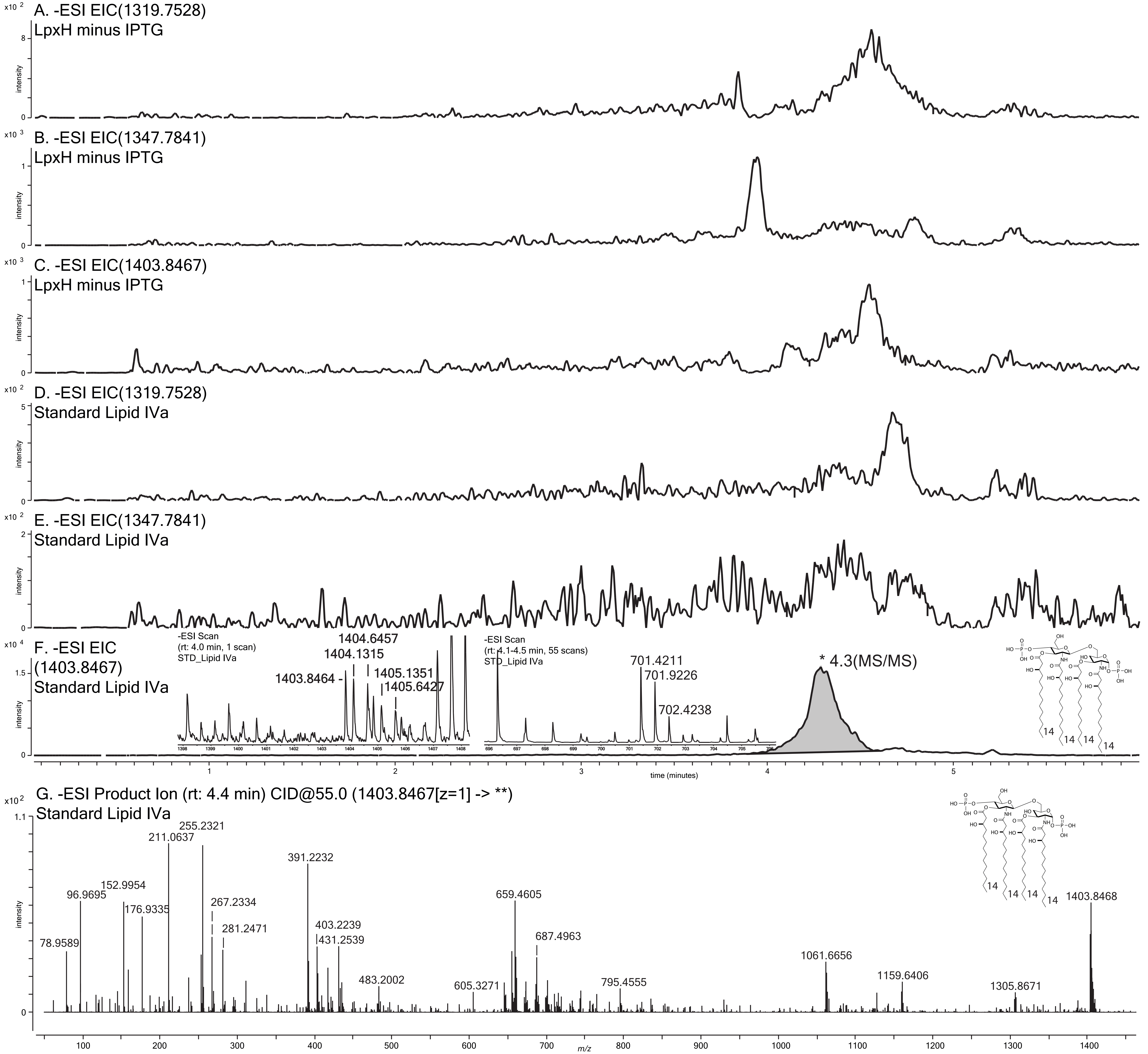

A. -ESI EIC(1319.7528)
LpxH minus IPTG
B. -ESI EIC(1347.7841)
LpxH minus IPTG
C. -ESI EIC(1403.8467)
LpxH minus IPTG
D. -ESI EIC(1319.7528)
Standard Lipid IVa
E. -ESI EIC(1347.7841)
Standard Lipid IVa
F. -ESI EIC (1403.8467) Standard Lipid IVa
-ESI Scan (rt: 4.0 min, 1 scan) STD_Lipid IVa
1403.8464
1404.1315
1404.6457
1405.1351
1405.6427
-ESI Scan (rt: 4.1-4.5 min, 55 scans) STD_Lipid IVa
701.4211
701.9226
702.4238
* 4.3(MS/MS)
time (minutes)
G. -ESI Product Ion (rt: 4.4 min) CID@55.0 (1403.8467[z=1] -> **)
Standard Lipid IVa
78.9589
96.9695
152.9954
176.9335
211.0637
255.2321
267.2334
281.2471
391.2232
403.2239
431.2539
483.2002
605.3271
659.4605
687.4963
795.4555
1061.6656
1159.6406
1305.8671
1403.8468
intensity
m/z
